# Systematic identification and evolutionary features of rhesus monkey small nucleolar RNAs

**DOI:** 10.1186/1471-2164-11-61

**Published:** 2010-01-25

**Authors:** Yong Zhang, Jun Liu, Chunshi Jia, Tingting Li, Rimao Wu, Jie Wang, Ying Chen, Xiaoting Zou, Runsheng Chen, Xiu-Jie Wang, Dahai Zhu

**Affiliations:** 1 National Laboratory of Medical Molecular Biology, Institute of Basic Medical Sciences, Chinese Academy of Medical Sciences, School of Basic Medicine, Peking Union Medical College, Beijing, PR China; 2 State Key Laboratory of Plant Genomics, Institute of Genetics and Developmental Biology, Chinese Academy of Sciences, Beijing, PR China; 3 Department of Medical Informatics, Peking University Health Science Center, Beijing, PR China; 4 Bioinformatics Laboratory, Institute of Biophysics, Chinese Academy of Sciences, Beijing, PR China

## Abstract

**Background:**

Recent studies have demonstrated that non-protein-coding RNAs (npcRNAs/ncRNAs) play important roles during eukaryotic development, species evolution, and in the etiology of disease. Rhesus macaques are the most widely used primate model in both biomedical research and primate evolutionary studies. However, most reports on these animals focus on the functional roles of protein-coding sequences, whereas very little is known about macaque ncRNAs.

**Results:**

In the present study, we performed the first systematic profiling of intermediate-size ncRNAs (50 to 500 nt) from the rhesus monkey by constructing a cDNA library. We identified 117 rhesus monkey ncRNAs, including 80 small nucleolar RNAs (snoRNAs), 29 other types of known RNAs (snRNAs, Y RNA, and others), and eight unclassified ncRNAs. Comparative genomic analysis and northern blot hybridizations demonstrated that some snoRNAs were lineage- or species-specific. Paralogous sequences were found for most rhesus monkey snoRNAs, the expression of which might be attributable to extensive duplication within the rhesus monkey genome. Further investigation of snoRNA flanking sequences showed that some rhesus monkey snoRNAs are retrogenes derived from L1-mediated integration. Finally, phylogenetic analysis demonstrated that birds and primates share some snoRNAs and host genes thereof, suggesting that both the relevant host genes and the snoRNAs contained therein may be inherited from a common ancestor. However, some rhesus monkey snoRNAs hosted by non-ribosome-related genes appeared after the evolutionary divergence between birds and mammals.

**Conclusions:**

We provide the first experimentally-derived catalog of rhesus monkey ncRNAs and uncover some interesting genomic and evolutionary features. These findings provide important information for future functional characterization of snoRNAs during primate evolution.

## Background

It is widely accepted that up to 90% of the human genome is transcribed into various types of RNAs [1-4]. However, only a very small proportion of transcripts (~2-3%) encode proteins. Although there is a possibility that many transcripts are simply noise [5], a considerable number of non-protein-coding RNAs (npcRNAs/ncRNAs) are produced [1-4]. The increasing numbers of ncRNAs found by systematic genome-wide screening have also demonstrated the widespread existence of ncRNAs in nature [6-9]. The ncRNAs can be categorized by length as 19~35 nt small ncRNAs such as miRNAs and piRNAs [10-12]; intermediate-size ncRNAs, ranging between 50 and 500 nt, such as the small nucleolar RNAs (snoRNAs) [13]; and long mRNA-like ncRNAs with sizes larger than 500 nt [14-18].

snoRNAs function mainly as modulators of ribosomal RNAs (rRNAs) [19], and represent the largest group of functional ncRNAs. Based on sequence and structural features, snoRNAs can be classified into two families-box C/D snoRNAs and box H/ACA snoRNAs-which guide site-specific 2'-O-ribose methylation and pseudouridylation of rRNA, respectively [20,21]. The spectrum of snoRNA targets is continuously growing. Some snoRNAs control methylation of tRNAs [22,23]. Small Cajal body RNAs (ScaRNAs), a subset of snoRNAs with box C/D and/or box H/ACA, regulate post-transcriptional modification of RNA polymerase II-transcribed snRNAs [24]. Recent findings have demonstrated that snoRNA can also target mRNA, to guide alternative splicing [25]. Another interesting discovery is that snoRNAs may be precursors of microRNAs and possess microRNA-like functions [26,27]. Together, available evidence suggests that snoRNAs may have broader functions than previously appreciated.

The genomic organization of snoRNA genes displays great diversity in different organisms. Unlike yeast and plants, in which snoRNAs are usually transcribed from independent polymerase II transcription units with dedicated promoters [28], most vertebrate snoRNAs reside in the introns of protein-coding or non-protein-coding genes and are generated by splicing-dependent processing [29,30]. Intron-encoded snoRNAs may also have special promoters to drive snoRNA transcription [31]. Many snoRNA genes have multiple paralogs derived from one or more duplications [32]. In nematodes, the paralogs of intron-encoded snoRNA genes were likely generated by *cis*- and *trans*-duplication mechanisms [23]. Luo and Li demonstrated that most human box H/ACA snoRNAs were retrogenes produced by L1 integration [33]. Weber reported that many mammalian snoRNAs were mobile genetic elements designated as snoRNA/scaRNA retroposons (snoRTs, scaRTs) [34]. Recently, Schmitz and colleagues discovered a platypus-specific snoRNA retroposon with powerful transposable activity that replicated a single snoRNA to form about 40,000 paralogs in the whole genome [35]. It is therefore possible that retroposition of snoRNA genes may have played an important role during evolution of mammalian genomes.

Based on these recent findings, it is likely that ncRNAs have important functions in almost every aspect of eukaryotic growth regulation. However, only a limited number and classes of ncRNAs have been discovered to date. Therefore, systematic identification of ncRNAs from various organisms is a critical primary step in the provision of a road map for functional studies of ncRNAs in various organisms. The rhesus macaque (*Macaca mulatta*) is the most thoroughly studied primate apart from humans. Although phylogenetically separated by more than 70 million years of evolution [36,37], rhesus macaques and humans are closely related and share a common ancestor dating back to about 25 million years ago [36,38]. Therefore, study of rhesus monkeys assists primate evolutionary research and modern biomedical programs [38,39]. A total of 21,905 protein-coding genes and 5,253 non-protein coding genes (including 715 predicted snoRNA loci) have been identified in the rhesus monkey genome by the ENSEMBL genome annotation group [6]. Although the expression pattern and possible functions of many protein-coding genes have been reported, identification of non-protein-coding genes of the rhesus monkey has relied only on computational predictions, by searching for sequences similar to those of known ncRNAs identified in other species. Such an approach is obviously inappropriate for identification of novel ncRNAs. Here, we conducted a systematic experimental identification of rhesus monkey ncRNAs by constructing a cDNA library derived from RNA fragments with sizes of 50 to 500 nt. We identified 117 non-redundant ncRNAs, including 80 snoRNAs and eight unclassified ncRNAs. We found that some of our identified ncRNAs were lineage- or species-specific. Further analysis of the genomic organization of these ncRNAs demonstrated that the majority represented snoRNAs with multiple paralogs in the rhesus monkey genome. Detailed analysis of the flanking sequences of each of the snoRNA paralogs revealed that some snoRNAs were retrogenes generated through L1-mediated integration machinery, suggesting that retroposon-mediated *trans*-duplication may have been a driving force for expansion of novel snoRNAs in the rhesus monkey genome.

## Results

### Systematic identification of rhesus monkey ncRNAs by analysis of a cDNA library

**Figure 1:**
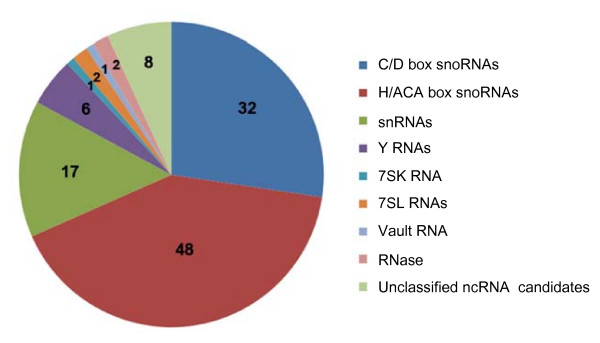
**Classification of 117 rhesus monkey ncRNAs**. The two RNase clones represent ribonuclease P RNA component H1 (RPPH1) and the RNA component of mitochondrial RNA processing endoribonuclease (RMRP).

Full-length intermediate-size ncRNA-enriched libraries (50~500 nt) were constructed using a previously described method [31], with minor modifications. This ensured that the libraries contained a substantial proportion of full-length ncRNA clones with defined 5' and 3' termini. The RNA used in library construction was extracted from the heart and skeletal muscle tissue of rhesus monkey. In total, 4,844 clones from two full-length cDNA libraries were sequenced. After discarding matches to tRNAs, rRNAs, and mRNAs, the remaining 835 sequences were considered to be putative ncRNAs and analyzed further. By merging redundant sequences and comparing the sequences and secondary structures of such putative ncRNAs with known ncRNAs annotated in the ENSEMBL and Rfam databases, the 835 clones were classified into 117 ncRNAs, including 80 snoRNAs (32 C/D box snoRNAs and 48 H/ACA box snoRNAs) representing 64 snoRNA families, 17 snRNAs, one 7SK RNA, six Y RNAs, two 7SL RNAs (SRP-RNA), one vault RNA, one ribonuclease P RNA component H1 (RPPH1), one RNA component of mitochondrial RNA processing endoribonuclease (RMRP), and eight unclassified ncRNA candidates (Figure 1 and Additional File 1).

All rhesus monkey snoRNAs identified in this study have known human homologs. Among 80 rhesus snoRNAs, 68 show perfect matches with the human homologs, whereas the other twelve rhesus snoRNAs are also highly conserved between monkey and human, with conservation scores over 0.96 (Table 1). In addition to showing homology in sequence and/or secondary structure with known human snoRNAs, all of our cloned snoRNAs had the conserved snoRNA motifs. In the 32 C/D box snoRNAs, we identified 52 pairs of the C/C' box with the D/D' box (Additional File 1). An H box and an ACA box were also found in the secondary structures of all H/ACA snoRNAs (Additional File 2). We further searched the sequences of each rhesus monkey snoRNA and the human homolog. The data showed that guide sequences and target sites were highly conserved between rhesus monkey and human (Additional Files 3 and 4).

**Table 1:** Northern data and sequence conservation scores of 117 rhesus monkey ncRNAs.

ncRNA Name	Clone Number	Length	Probe ID	Northern data			Conservation score			Consistance
				Chicken	Mouse	Human	Chicken	Mouse	Human	
**Group 1 **40 ncRNAs detected by 18 ncRNA probes										
7SK	9	332	MT721	yes	yes	yes	0.98	1	1	Y
RNaseP_nuc	1	340	MP733	yes	yes	yes	0.12	0.36	1	Y
SNORA61	1	131	MP2422	yes	yes	yes	0.42	0.36	1	Y
SNORA66	4	131	MP405	yes	yes	yes	0.69	0.83	1	Y
SNORA81	26	179	MP284	yes	yes	yes	0.42	0.99	1	Y
SNORD94	5	135	MP141	yes	yes	yes	0.64	1	1	Y
7SLa	1	299	MP477	yes	yes	yes	0.97	1	1	Y
7SLb	2	300	MP477	yes	yes	yes	0.97	1	1	Y
U1-1	14	164	MT369	yes	yes	yes	1	1	1	Y
U1-2	17	166	MT369	yes	yes	yes	1	1	1	Y
U1-3	23	166	MT369	yes	yes	yes	1	1	1	Y
U12	12	150	MT305	yes	yes	yes	0.87	1	1	Y
U2-1	5	184	MP76	no	yes	yes	0.99	1	1	Y
U2-2	21	188	MP76	no	yes	yes	0.99	1	1	Y
U3a	12	148	MT42	yes	yes	yes	0.70	0.81	1	Y
U3b	9	217	MT42	yes	yes	yes	0.72	0.8	1	Y
U3c	7	218	MT42	yes	yes	yes	0.71	0.82	1	Y
U3d	10	218	MT42	yes	yes	yes	0.70	0.80	1	Y
U3e	8	219	MT42	yes	yes	yes	0.73	0.82	1	Y
U3f	6	221	MT42	yes	yes	yes	0.70	0.81	1	Y
U3g	6	216	MT42	yes	yes	yes	0.71	0.83	1	Y
U3h	9	216	MT42	yes	yes	yes	0.72	0.81	1	Y
U3i	7	216	MT42	yes	yes	yes	0.70	0.80	1	Y
U3j	2	216	MT42	yes	yes	yes	0.73	0.82	1	Y
U3k	20	217	MT42	yes	yes	yes	0.71	0.81	1	Y
U3l	5	217	MT42	yes	yes	yes	0.73	0.80	1	Y
U3m	7	217	MT42	yes	yes	yes	0.72	0.84	1	Y
U3n	6	217	MT42	yes	yes	yes	0.70	0.83	1	Y
U4	10	146	MT365	yes	yes	yes	0.99	0.99	1	Y
U5-1	1	97	MP235	yes	yes	yes	0.70	0.99	0.88	Y
U5-2	6	118	MP235	yes	yes	yes	0.75	0.99	0.88	Y
U5-3	9	121	MP235	yes	yes	yes	0.74	0.99	0.88	Y
U5-4	8	121	MP235	yes	yes	yes	0.73	0.99	0.88	Y
U5-5	6	124	MP235	yes	yes	yes	0.78	0.99	0.88	Y
U6-1	35	89	MT44	yes	yes	yes	1	1	1	Y
U6-2	48	104	MT44	yes	yes	yes	1	1	1	Y
U6atac	43	127	MT65	yes	yes	yes	0.96	0.96	1	Y
unclassified 2	1	51	MT4702	yes	yes	yes	0.94	0.94	0.94	Y
Y5	30	102	MP281	yes	yes	yes	0.62	1	1	Y
Y6	81	112	MP91	yes	yes	yes	1	1	1	Y
**Group 2 **51 ncRNAs detected by 49 ncRNA probes										
RNase_MRP	1	146	MP690	no	yes	yes	0.15	0.39	1	Y
SCARNA11	2	144	MP66	no	yes	yes	0.16	0.85	1	Y
SCARNA15	2	127	MP77	no	yes	yes	0.22	0.99	1	Y
SCARNA25	3	142	MP407	no	yes	yes	0.17	1	0.98	Y
SCARNA4	3	128	MP400	no	yes	yes	0.23	0.98	1	Y
SNORA11	1	115	MP4400	no	yes	yes	0.20	0.51	1	Y
SNORA13	1	135	MP243	no	yes	yes	0.19	0.99	1	Y
SNORA14	2	132	MP227	no	yes	yes	0.20	0.99	1	Y
SNORA15	2	138	MP236	no	yes	yes	0.20	0.46	0.98	Y
SNORA17	2	134	MP695	no	yes	yes	0.16	0.87	1	Y
SNORA18	5	133	MP17	no	yes	yes	0.23	0.98	1	Y
SNORA2	1	137	MP4598	no	yes	yes	0.17	0.99	1	Y
SNORA23	2	189	MP268	no	yes	yes	0.23	0.94	1	Y
SNORA24	2	130	MP252	no	yes	yes	0.41	1	1	Y
SNORA28	3	129	MP306	no	yes	yes	0.31	0.92	1	Y
SNORA36a	3	132	MP207	no	yes	yes	0.42	0.98	1	Y
SNORA36b	2	132	MP207	no	yes	yes	0.45	0.52	1	Y
SNORA4	6	140	MP374	no	yes	yes	0.17	0.44	1	Y
SNORA41	1	131	MP4579	no	yes	yes	0.20	0.56	0.98	Y
SNORA42	1	139	MP4011	no	yes	yes	0.22	0.86	1	Y
SNORA49	2	138	MP427	no	yes	yes	0.20	0.88	1	Y
SNORA5	3	135	MP4812	no	yes	yes	0.45	0.84	1	Y
SNORA53	2	248	MP750	no	yes	yes	0.29	1	1	Y
SNORA54	2	124	MP319	no	yes	yes	0.24	1	1	Y
SNORA58	4	137	MP4121	no	yes	yes	0.21	0.52	1	Y
SNORA62	8	150	MP86	no	yes	yes	0.17	1	0.99	Y
SNORA63	13	135	MP32	no	yes	yes	0.19	0.99	1	Y
SNORA7	1	140	MP4777	no	yes	yes	0.20	0.96	1	Y
SNORA70	1	135	MP4769	no	yes	yes	0.23	0.70	1	Y
SNORA71	3	138	MP241	no	yes	yes	0.19	0.83	1	Y
SNORA72	5	132	MP356	no	yes	yes	0.19	0.96	1	Y
SNORA74a	2	134	MP2697	no	yes	yes	0.12	1	1	Y
SNORA74b	2	203	MP4370	no	yes	yes	0.12	1	1	Y
SNORD13	23	104	MT370	no	yes	yes	0.21	0.74	1	Y
SNORD15	2	142	MP4	no	yes	yes	0.23	1	1	Y
SNORD17a	2	250	MP20	no	yes	yes	0.12	1	0.99	Y
SNORD17b	6	257	MP20	no	yes	yes	0.12	1	0.99	Y
SNORD22	1	126	MP4712	no	yes	yes	0.21	1	1	Y
SNORD46	3	101	MP329	no	yes	yes	0.26	0.96	0.96	Y
SNORD67	1	104	MP4243	no	yes	yes	0.45	0.98	0.98	Y
snoU6-53	3	110	MP277	no	yes	yes	0.24	0.98	1	Y
U11	4	136	MP1393	no	yes	yes	0.29	1	1	Y
U4atac	35	131	MT4	no	yes	yes	0.19	0.98	1	Y
unclassified 6	1	137	MP2665	no	yes	yes	0.18	0.26	0.99	Y
unclassified 7	2	143	MP232	no	yes	yes	0.17	0.99	1	Y
Vault RNA	2	89	MP331	no	yes	yes	0.21	0.25	0.53	Y
SNORD116	3	90	MT4173	no	no	yes	0.28	0.77	1	Y
SNORA31	7	128	MP312	no	yes	yes	0.66	1	1	N
SNORA73	1	119	MP4480	no	yes	yes	0.97	0.97	0.96	N
SNORA8	1	140	MP4084	no	yes	yes	0.69	0.99	1	N
SNORD16	1	94	MT2039	no	yes	yes	0.85	1	1	N
**Group 3 **16 ncRNAs detected by 14 probes										
SNORA25	2	133	MP3076	no	no	yes	0.18	0.56	0.99	Y
SNORA64	1	132	MP4716	no	no	yes	0.18	0.21	1	Y
SNORA68	1	133	MP2580	no	no	yes	0.18	0.32	1	Y
SNORD24	1	71	MT325	no	no	yes	0.32	0.32	1	Y
U8	8	137	MT180	no	no	yes	0.17	0.46	1	Y
unclassified 8	1	153	MP472	no	no	yes	0.16	0.18	1	Y
Y1	1	77	MP727	no	no	yes	0.27	0.51	0.92	Y
Y2	4	90	MP727	no	no	yes	0.26	0.49	0.92	Y
Y3	34	90	MP727	no	no	yes	0.25	0.53	0.92	Y
Y4	25	96	MT274	no	no	yes	0.27	0.27	1	Y
SNORA19	4	132	MP4158	no	no	yes	0.40	0.99	1	N
SNORA27	3	128	MP464	no	no	yes	0.22	0.98	1	N
SNORA40	1	130	MP4587	no	no	yes	0.22	0.77	1	N
SNORA43	1	134	MP2188	no	no	yes	0.16	0.76	1	N
SNORA76	1	135	MP1373	no	no	yes	0.18	0.93	0.99	N
snosnR60_Z15	1	68	MT2333	no	no	yes	1	1	1	N
**Group 4 **8 ncRNAs detected by 8 probes										
SNORA20	2	133	MP4330	no	no	no	0.18	0.18	1	N
SNORD26	1	139	MP2208	no	no	no	0.20	0.97	0.98	N
SNORD27	2	69	MP299	no	no	no	0.36	0.72	1	N
SNORD87	1	77	MP4391	no	no	no	0.31	0.48	1	N
unclassified 1	7	51	MT3898	no	no	no	0.63	1	1	N
unclassified 3	2	52	MT3338	no	no	no	**	**	**	**
unclassified 4	1	67	MP2207	no	no	no	0.32	0.35	0.65	N
unclassified 5	2	75	MP2233	no	no	no	0.33	0.55	0.99	N
**Group 5 **Only one ncRNA belongs to this group										
SNORD45	1	77	MP2242	no	yes	no	0.34	1	1	N
**Group 6 **Only one ncRNA belongs to this group										
SNORA50	3	136	MP2895	yes	no	yes	0.98	1	1	N

** Without genomic loci in the database of the current genomic version.

### Expression patterns of the 117 ncRNAs in tissues of rhesus monkey and other species

**Figure 2:**
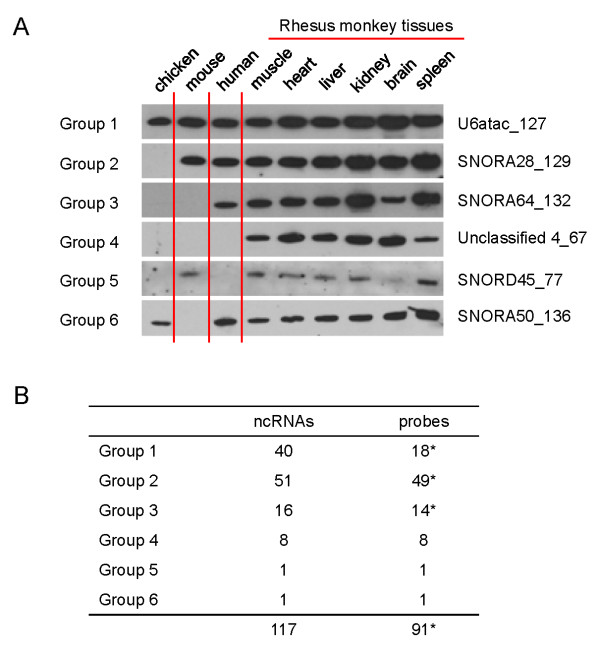
**Six groups of ncRNAs based on expression patterns in human, murine, and chicken tissues**. **A**. Representative ncRNA expression patterns from each of the six groups of ncRNAs. The expression of 117 rhesus monkey ncRNAs was examined by northern blotting using 5 μg aliquots of total RNA from monkey spleen, brain, kidney, liver, heart, and skeletal muscle. Total RNA from human, murine, and chicken skeletal muscle were included in each RNA blot to test expression in different species. Based on northern blot analysis, the expression patterns in various species could be classified into six types. One representative ncRNA from each of the six groups is shown. All ncRNAs are labeled as Name_size on the left side of each northern blot. **B**. Summary of ncRNA numbers in each of the six groups. * The total number of the probes is less than the total number of ncRNAs because several ncRNA members in the same snoRNA family can be recognized by the same probe.

The expression of the 117 ncRNAs was confirmed by northern blotting (Additional File 5). All tested ncRNAs were expressed in the six examined tissues of rhesus monkey (spleen, brain, kidney, liver, heart, and skeletal muscle). Several quantitative differences in the expression abundance of ncRNAs were observed among different tissues, but no tissue-specific expression pattern could be discerned. We also investigated expression of the 117 monkey ncRNAs in the skeletal muscle tissue of human, mouse, and chicken, by northern blotting (Additional File 5). Based on the observed expression patterns, the 117 ncRNAs could be classified into six groups (Figure 2). Group 1 ncRNAs were expressed in chicken, mouse, human, and all examined rhesus monkey tissues; group 2 ncRNAs were detected in monkey, human, and mouse, but not in chicken; group 3 ncRNAs were expressed in monkey and human; and group 4 ncRNAs were detected only in rhesus monkey tissues. Interestingly, SNORD45 was expressed only in mouse and monkey (group 5); SNORD50 was detected in chicken, human, and rhesus monkey, but was absent from the mouse (group 6). To rule out the possibility that the lack of detectable signals in northern blotting was caused by tissue-specific expression of lineage/species-specific ncRNAs, we investigated the synthesis of these materials in nine human and mouse tissues, but no signals were detected (Additional File 5).

Conservation analysis of rhesus ncRNAs using the BLAST algorithm, as well as comparison with human, mouse, and chicken genomic sequences, demonstrated that most (96/117) lineage/species-specific expression patterns were supported by sequence homology, although expression of some highly conserved sequences was not detected (Table 1). The expression patterns of ten ncRNAs in groups 4, 5, and 6 were inconsistent with the conservation scores of their genomic sequences across different species. For example, eight ncRNAs of group 4 showed conserved sequences but no detectable expression in human tissues (Table 1 and Additional File 5). It is possible that the homologs of these ncRNAs are pseudogenes, or are expressed at levels below the threshold of sensitivity of the northern blot. Alternatively, the homologs might be transcriptionally regulated in a spatio-temporal fashion, or by physiological or pathological stimuli/stresses, and would thus not be constitutively expressed under normal conditions.

### Comparative genomic analysis of rhesus monkey snoRNAs

The secondary structures and functional boxes of snoRNAs were found to be highly conserved [21], but the nucleotide sequences outside of the hallmark boxes and the antisense regions of snoRNAs changed during vertebrate evolution. To investigate the sequence conservation of snoRNAs over the course of primate evolution, we plotted the sequences of 64 rhesus monkey snoRNA families against those of eight other primate genomes. As genomic sequences of some species are incomplete, only 25 snoRNA families showed identifiable homologs in all eight primate species examined. Sequence alignment data showed that some snoRNAs sequences diverged even among closely related primates. The sequence alignments of the top five divergent snoRNAs are shown in Additional File 6.

**Figure 3:**
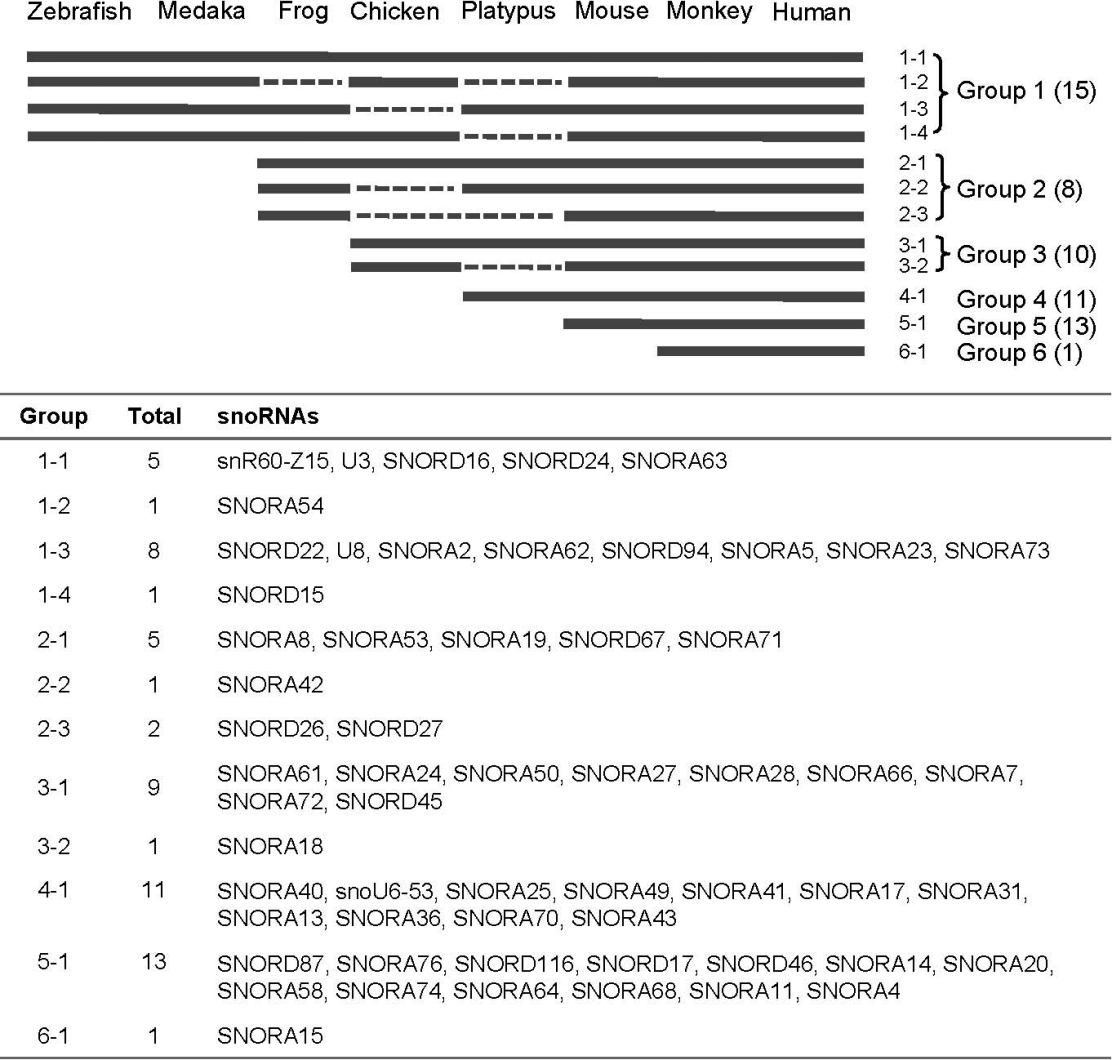
**Presence or absence of rhesus monkey snoRNAs in other vertebrates**. The presence or absence of each snoRNA was analyzed according to ENSEMBL annotations (release 50).

To determine when rhesus monkey snoRNAs appeared during vertebrate evolution, we searched for homologs of 58 rhesus monkey snoRNA families (six families were excluded because of a lack of annotation in either or both of the human and mouse datasets) in seven other representative vertebrates, based on annotations in ENSEMBL Release 50 (Additional File 7). Among the 58 rhesus monkey snoRNA families, 15 shared homologs even in zebrafish and medaka (Figure 3, Group 1), indicating that these snoRNAs appeared early in vertebrate evolution. Eight snoRNA families were detected in reptiles and evolutionarily later species (Figure 3, Group 2), and 10 snoRNA families appeared after the emergence of birds (Figure 3, Group 3). The remaining 25 snoRNA families were present in mammals but clearly absent in birds and other non-mammalian species (Figure 3, Groups 4-6). Thirteen of 25 mammalian snoRNA families did not have homologs in the platypus genome (Group 5), suggesting a later emergence in mammalian evolution. Finally, one snoRNA without a homolog in the mouse may be primate-specific (Group 6).

### SnoRNA expansion during vertebrate evolution

**Figure 4:**
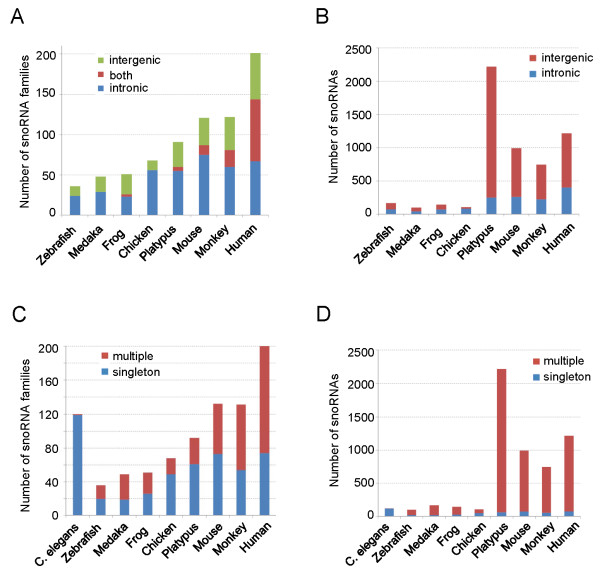
**snoRNA expansion in vertebrates**. The number of snoRNA families (**A**) and the total number of snoRNA copies (**B**), based on the genomic organization, were calculated in each of eight vertebrate species. The number of snoRNA families (**C**) and all snoRNA individuals (**D**), with single or multiple genomic loci in the eight vertebrate species, are also shown.

The total number of snoRNA-encoding genes increased during vertebrate evolution, based on data from the ENSEMBL genome annotation project [6]. We asked whether this increment in snoRNA genes was attributable to the generation of multiple paralogs by duplication mechanisms, or arose *de novo *by accumulation of nucleotide mutations, or was attributable to the action of other driving mechanisms. Of course, these possibilities may be combined. To address this question, we collected all predicted and validated snoRNA sequences from eight representative vertebrate species represented in the ENSEMBL database, including zebrafish, medaka, frog, chicken, platypus, mouse, rhesus monkey, and human, and calculated the total number of snoRNA genes as well as the number of snoRNA families (any snoRNA family could contain a single copy snoRNA or have multiple paralogs in the genome). As shown in Figure 4A, the number of snoRNA families increased during vertebrate evolution, indicating a *de novo *origin of snoRNA genes. In addition, the number of intron-encoded snoRNAs rose significantly in birds and thereafter appeared in mammals, contributing extensively to the expansion of snoRNA families (Figure 4A). The total number of snoRNA-encoded genes increased suddenly in mammals after the divergence from birds. Also, the expansion of mammalian snoRNAs usually involved intergenic-encoded snoRNAs, and the principal contribution to expansion was the production of many members of such snoRNA families (Figure 4B and 4D). The number of predicted snoRNA genes in medaka, zebrafish, frog, and birds is less than 200, but the numbers increased to 2,217 in the platypus, 992 in the mouse, and 744 in the rhesus monkey genome. As shown in Figure 4C, compared to *Caenorhabditis elegans*, in which nearly all snoRNAs exist as single copies (singletons), 30~60% of vertebrate snoRNA families have multiple paralogs, demonstrating that large-scale duplications of particular snoRNA families may have occurred during vertebrate evolution (Figure 4D). Among the 58 identified rhesus monkey snoRNA families with annotated orthologs in human and mouse, 14 are singletons, and the remaining 44 snoRNA families have 315 paralogs in the rhesus monkey genome (Additional File 7).

The expansion mode of snoRNAs differed among the species examined. For example, the rhesus monkey, mouse, and platypus genomes each contain no more than three copies of SNORAU13, but 439 copies may be found in the human genome. However, the SNORA17 family has no more than three copies in the rhesus and human genomes, but 354 members may be found in the mouse genome.

### Duplication mechanisms of rhesus monkey snoRNAs

**Figure 5:**
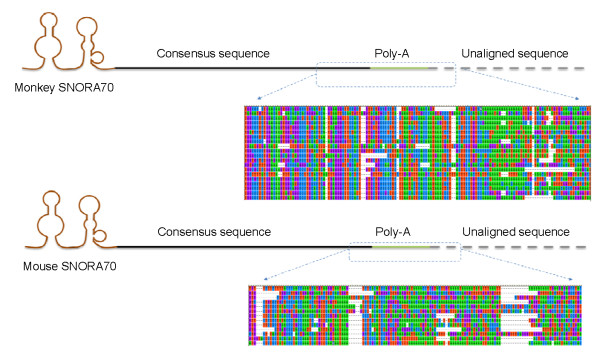
**Structures of SNORA70 paralogs in rhesus monkey and mouse**. Each SNORA70 paralog is composed of a 5' H/ACA box snoRNA following a 3' consensus sequence and a poly(A) structure. The alignments of 20 rhesus monkey SNORA70 and 13 mouse SNORA70 paralogs in the boxed regions are also shown.

**Figure 6:**
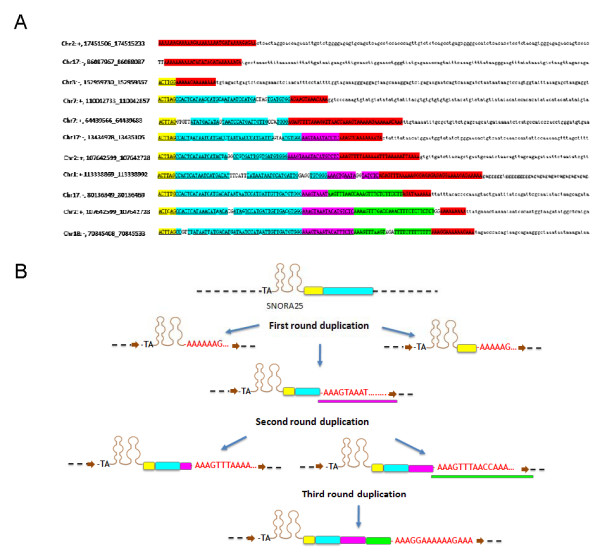
**Proposed model for rhesus monkey SNORA25duplication**. **A**. 3' flanking sequence alignment of eleven rhesus monkey SNORA25 paralogs. The colored sequences represent different consensus motifs. Red, green, and pink blocks are poly(A) structures. Yellow and blue boxes represent two other consensus sequences. **B**. Proposed model for rhesus monkey SNORA25 duplication. The adjacent T and A are the two conserved nucleotides at the immediate 5' end of SNORA25. Different colored blocks represent various consensus motifs as described in A above. Poly(A) sequences are highlighted in red. Target site duplications (TSDs) are shown with brown arrows.

According to ENSEMBL annotations, eight rhesus monkey snoRNA families are predicted to have more than ten paralogs. As shown in Table 2, the majority of high-copy snoRNAs are present in the three examined mammalian species, and most are duplicated in a species-specific fashion. This suggests that most high-copy snoRNAs were replicated in recent evolutionary times, after the speciation of mammals. To explore driving forces for the high duplication rate of snoRNAs in mammalian species, we analyzed the flanking sequences of each paralog within individual snoRNA families to search for putative transposable elements mediating snoRNA expansion. We found that the paralogs of SNORA70 in the rhesus monkey and mouse genomes shared a ~490 bp consensus sequence in the 3' flanking regions (Figure 5). To investigate whether a particular transposable element (TE) mediated the duplication of SNORA70 in monkey and mouse genomes, we first searched for known TEs in the flanking sequences of SNORA70 using RepeatMasker [40]. However, no known transposable element was identified in the flanking sequences. A genomic BLAST search of the consensus sequence did not show a high copy-number in either the rhesus monkey or mouse genome, suggesting that a novel TE did not exist in the consensus sequence. Thus, the duplication of SNORA70 paralogs most likely occurred *via *a non-TE mediated mechanism. The SNORA25 family includes 16 paralogs with apparently random distribution in the rhesus monkey genome. Each duplication unit possesses typical SINE-like retroposon structural features characterized by a poly(A) end and a target site duplication (TSD) [41]. The 3'-flanking sequences of eleven SNORA25 paralogs of the rhesus monkey are shown in Figure 6A. Interestingly, six SNORA25 paralogs have multiple poly(A) sequences (Figure 6A), suggesting that some rhesus monkey SNORA25 sequences might have undergone several rounds of duplication, to create the variant paralogs (Figure 6B).

**Table 2:** High-copy snoRNAs with more than ten paralogs*.

snoRNA	zebrafish	medaka	xenopus	chicken	platypus	mouse	monkey
SNORA7	0	0	0	1	1979	3	6
SNORD116	0	0	0	0	0	27	32
SNORA25	0	0	0	0	2	7	16
SNORA70	0	0	0	0	2	30	29
SNORA17	0	0	0	0	1	354	3
SNORA71	0	0	2	1	1	15	9
U3 snoRNA	40	38	7	1	4	15	50
U8 snoRNA	8	0	5	0	2	2	13

* The total number of genomic loci for each snoRNA family is based on ENSEMBL genome annotation data (Release 50).

**Figure 7:**
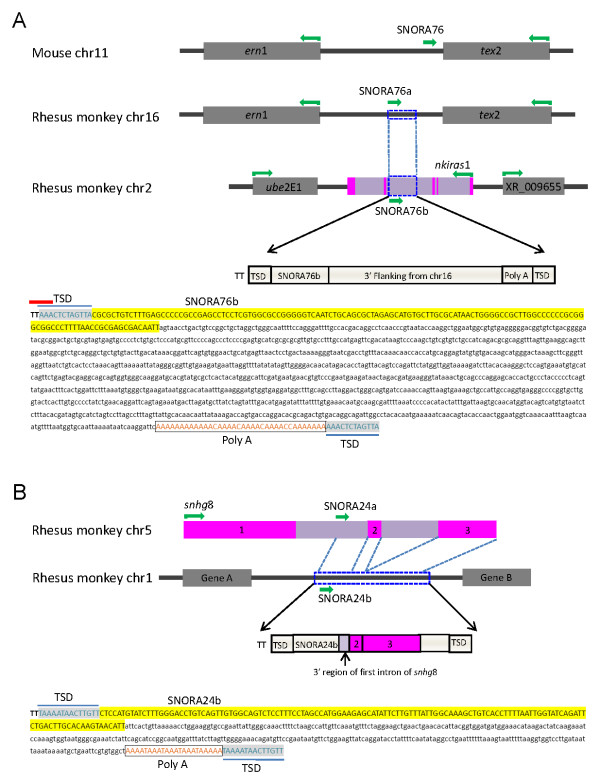
**Trans-duplication of SNORA76 (A) and SNORA24 (B)**. Gray boxes represent gene regions and black lines intergenic regions. Green arrows show transcriptional orientation. Pink boxes represent exons and purple boxes introns. Open blue boxes show trans-duplicated regions. The nucleotide sequence of each trans-duplicated region is shown. SnoRNA sequences are colored yellow. Retroposed nucleotides, with the corresponding snoRNAs, are shown in lower case. Poly(A) sequences are shown as open boxes. TSD sequences are shaded. L1 consensus recognition sites (for T2A4 derivatives) are indicated as red bars above 5' sequences.

Two paralogs of rhesus monkey SNORA76 were also examined. One (designated as SNORA76a) is located in an intergenic region on chromosome 16, the other (designated SNORA76b) is located on chromosome 2 within the intron of NF-kappa-B inhibitor-interacting Ras-like protein 1 (*nkiras*1). There is one copy of SNORA76 in the mouse genome. Based on syntenic region analysis between mouse and rhesus monkey, SNORA76a is likely to be the parental copy in the rhesus monkey genome. The SNORA76b paralog is probably a novel progeny copy that possibly arose after the divergence of rodents and primates. This paralog seems to be rhesus monkey-specific, as SNORA76b is absent in the syntenic region of the marmoset, orangutan, chimpanzee, and human. The 3'-flanking sequences of SNORA76b and SNORA76a share about 1,200 nt, suggesting that SNORA76a was translocated together with the 3' flanking sequence, from chromosome 16 to chromosome 2, to create the novel SNORA76b paralog (Figure 7A).

SINE-like expansion was also observed among some snoRNA families. The flanking sequences of SNORA76b contain a terminal poly(A), a TSD, and T2A4 derivatives preferably recognized by the L1 nicking endonuclease, all of which are features of SINE family transposons. Therefore, we hypothesize that SNORA76b may be a SINE-like retrogene generated using the L1 integration machinery. Figure 7B shows another example of snoRNA *trans*-duplication in the rhesus monkey genome. There are six copies of the SNORA24 gene in this genome. One copy of SNORA24 (SNORA24a) on chromosome 5 is located in the first intron of a gene termed the human *snhg*8 homolog (small nucleolar RNA host gene 8; *snhg*8). SNORA24b on chromosome 1 possesses characteristics typical of a SINE-like retrogene (with a TSD and a polyA structure) and the immediate downstream region of rhesus SNORA24b is composed of three segments that could be aligned to the 3' region of the first intron, and the entire sequences of exon 2 and exon 3, of the human *snhg*8 gene, respectively. The genomic composition of the flanking region of rhesus monkey SNORA24b is evidence that this snoRNA locus was generated in an RNA-mediated retro-transposition event and that the transposed unit originated from a partially processed hnRNA of *snhg8*. As a result, SNORA24 together with the 3' segment of the *sngh8 *transcript and the polyA end thereof retroposed to a new locus on chromosome 1, the SNOR24b locus (Figure 7B). Apart from these two examples, we also identified another 22 potential rhesus monkey snoRNA retrogenes (Additional File 8). In summary, our data suggest that SINE-like retroposon-mediated retroposition might represent a driving force for rhesus monkey snoRNA expansion.

### Analysis of snoRNA host genes

**Figure 8:**
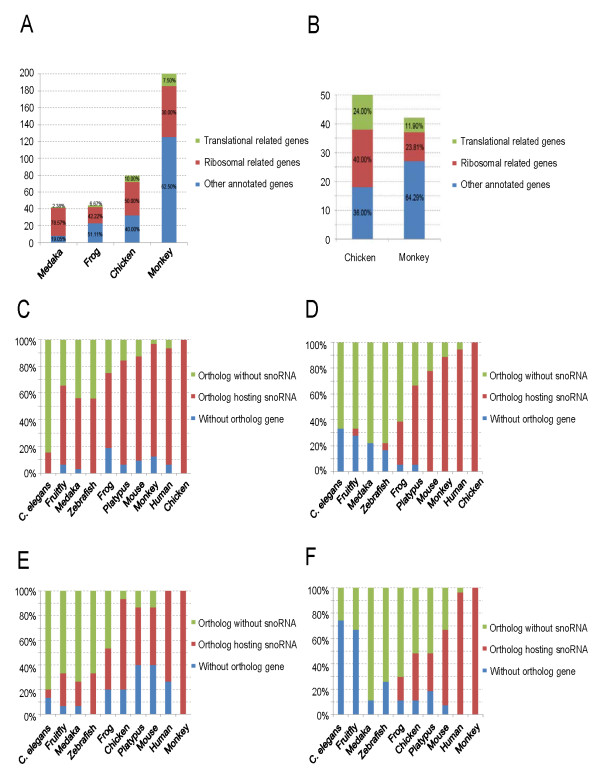
**Ortholog analysis of snoRNAs and their host genes**. **A**. Functional distribution of host genes of all predicted snoRNAs in four representative vertebrate species. **B**. Functional distribution of host genes of experimentally validated snoRNAs in rhesus monkey and chicken. **C**. Ortholog analysis of validated chicken snoRNA host genes with ribosome/translation-related functions in nine species. **D**. Ortholog analysis of validated chicken snoRNA host genes with non-ribosome/non-translation-related functions in nine species. **E**. Ortholog analysis of validated rhesus monkey snoRNA host genes with ribosome/translation-related functions in nine species. **F**. Ortholog analysis of validated rhesus monkey snoRNA host genes with non-ribosome and non-translation-related functions in nine species.

A large proportion of vertebrate snoRNAs are encoded in the introns of protein-coding or non-protein-coding genes. Although snoRNA host genes with ribosome-translation-related functions were the first to be reported, some snoRNAs are also hosted by non-ribosome or non-translation-related genes. Here, we systematically analyzed the functional spectrum of host genes for all intronic snoRNAs predicted in four representative vertebrates (the data are from ENSEMBL release 50), including medaka, frog, chicken, and rhesus monkey. As shown in Figure 8A, more than 80% of snoRNA host genes in medaka are ribosome-related protein-coding genes, whereas this percentage decreases to 30% in the rhesus monkey. Similar patterns were evident in the functional distribution of experimentally validated snoRNA host genes when the chicken and rhesus monkey were compared (Figure 8B). The data suggests that snoRNA-encoding genes expanded in the introns of non-ribosomal and non-translational protein-coding genes during vertebrate evolution.

We also searched for gene orthologs hosting snoRNAs in eight additional species, including *C. elegans*, fruit fly, medaka, zebrafish, frog, platypus, mouse, and human. Interestingly, we found that almost all chicken snoRNAs and host genes thereof had orthologs in humans and the rhesus monkey (Figure 8C and Figure 8D), suggesting that birds and primates shared not only snoRNAs but also the host genes from a common ancestor dating back more than 310 million years ago. A large proportion (about 80%) of rhesus monkey snoRNA host genes with ribosome- and translation-related functions have orthologs in the chicken genome, and the chicken orthologs are also hosts of snoRNAs (Figure 8E). However, only 37% of the orthologs of non-ribosome and non-translation-related rhesus monkey snoRNA host genes carried snoRNAs in the chicken genome (Figure 8F), indicating that the majority of monkey snoRNAs encoded by introns of non-ribosome-related genes appeared after the divergence of birds and mammals.

## Discussion

Recent studies have demonstrated that the functions of non-protein-coding RNAs may encompass almost every aspect of biological activity in normal development and disease biogenesis [21,25,42-45]. Rhesus macaques are a suitable primate model for basic and applied biomedical research [38,39]. However, in contrast to the considerable literature on human and mouse ncRNAs, rhesus monkey ncRNAs have not previously been systematically characterized. Here, we performed a detailed screening of the rhesus monkey intermediate-size ncRNA transcriptome and cloned 117 rhesus monkey ncRNAs, including 80 snoRNAs, eight unclassified ncRNAs, and 29 known RNAs (snRNAs, Y RNA, and others). By comparative genomics analysis, we found several lineage- or species-specific snoRNAs. Genomic organization analysis showed that the majority of rhesus monkey snoRNAs have many paralogs in the rhesus monkey genome. By flanking sequence analysis, we found that SINE-like retroposon-mediated *trans*-duplication may have been an important mechanism in expansion of novel snoRNAs in the rhesus monkey genome.

Among the 117 identified rhesus monkey ncRNAs, eight unclassified ncRNA candidates could not be assigned to any known class of ncRNA. These eight unclassified ncRNAs were ubiquitously expressed in the six rhesus monkey tissues tested. Recently, we also identified nine unclassified ncRNAs from the chicken [46]. Previous reports also showed that some ncRNAs obtained from cDNA library sequencing did not belong to any known ncRNA family, and these ncRNAs were designated as unclassified or unknown [13,31,47]. Hüttenhofer and coworkers found 57 such unclassified ncRNAs, of length 50~500 nt, in mouse brain cDNA libraries [13]. Deng et al. reported 14 unclassified ncRNAs of length 70~200 nt in a *C. elegans *cDNA library [31]. Yuan identified 29 unclassified ncRNAs by constructing cDNA libraries from four developmental stages of *Drosophila melanogaster *[48]. These unclassified ncRNAs often show little sequence conservation and are less prevalent compared to known snoRNAs. However, these observations do not mean that the unclassified ncRNAs are non-functional [13,31,48]. The increasing number of newly identified unclassified ncRNAs suggests that other types/classes of ncRNA of intermediate size (50~500 nt) remain to be identified, and novel ncRNA families will likely be susceptible to classification using enhanced bioinformatic comparisons and extensive functional studies of the roles played by such ncRNAs.

Previous reports showed that the majority of known snoRNAs were conserved between human and mouse, at a level of 80~90% [49]. Most rhesus monkey snoRNAs identified in the present study show high homology to those of the human and mouse. However, 13 snoRNAs had a conservation score below 0.6 (Table 1), suggesting that some snoRNAs are less conserved between primates and rodents. Using comparative genomics analysis, we found several lineage- or species-specific snoRNAs. Fifteen snoRNA families were ancient, being present at an early stage of vertebrate evolution, whereas 11 snoRNA families appeared after the divergence of birds and mammals. Fourteen young snoRNA families arose during mammalian evolution and one of these (SNORA15) developed only after primates had arisen. Our findings are in line with recent studies in other species. Previously, we found 30 chicken/bird-specific ncRNAs [46], and Schmitz reported 49 platypus-specific snoRNAs [35]. Computer analysis of human genomic tiling array data revealed 300 putative candidates for classification as primate-specific ncRNAs [50]. Together with previous reports, our data show that ncRNAs may play important roles in lineage development, or speciation, during evolution.

Although homologs of some rhesus monkey snoRNAs could be found in the mouse and human genomes, the expression of several snoRNAs was not detectable by northern blotting, suggesting that some snoRNA homologs might be pseudogenes without transcriptional potential in the human and/or mouse. Thus, we found only 14 potential primate-specific and eight rhesus monkey (or non-human primate)-specific transcripts (Table 1 and Figure 2). However, it remains possible that undetectable expression in the human or mouse might be attributable to transcriptional regulation by spatio-temporal, physiological, or pathological stimuli/stresses that were not present under the normal conditions prevalent when our tissue samples were taken. In support of this hypothesis, several examples of tissue-specific expression of ncRNAs have been reported in previous studies describing brain-specific snoRNAs or snoRNAs involved in neuronal development [51]. By analogy, some microRNAs and piRNAs display specific spatio-temporal expression patterns, and play functional roles in cell differentiation and organogenesis during development [11,12,52,53]. In the present study, we also found that SNORA71, ubiquitously expressed in human and rhesus monkey tissues, is predominantly expressed in the brain of mouse.

In vertebrates, most snoRNAs are located within introns of protein-coding or non-protein-coding genes [21,54]. Some snoRNAs are present as several copies, either in different introns of the same gene or within introns of different genes [32,55]. Genomic organization analysis showed that the majority of the rhesus monkey snoRNAs identified in this study have multiple paralogs in the rhesus genome, suggesting redundancy arising from duplication, including transposition. Diverse molecular mechanisms may be involved in the creation of protein-coding genes, such as gene duplication and retroposition [56]. To investigate the mechanisms of rhesus monkey snoRNA expansion, we analyzed the flanking sequences of each snoRNA paralog and found that these sequences adjacent to some rhesus monkey snoRNAs have a typical SINE-like retroposon characterized by a poly(A) end and TSDs, suggesting that some rhesus monkey snoRNA paralogs are retrogenes formed by autonomous retroposon-mediated retroposition. In addition, the 5' flanking sequences of rhesus monkey SNORA76b and SNORA24b possess T2A4 motifs, which are preferentially recognized by the L1 retroposon-encoded nicking endonuclease, suggesting that SNORA76b and SNORA24b were generated from a parental copy by L1 integration machinery-mediated retroposition. Significantly, we found that six paralogs of SNORA25 also possess typical SINE-like retroposon characteristics, and contain multiple poly(A) sequences, indicating that SNORA25 underwent multiple duplication events during evolution. Thus, we propose a model involving retroposition for SNORA25 duplication. Recently, the mechanisms of snoRNA gene expansion in other species have been reported. In nematodes, some snoRNA paralogs were generated by *cis*- or *trans*-duplication [23]. Other data suggest that mammalian snoRNA genes are SINE-like retroposons (snoRTs/snoRTEs), and that retroposition mediated by snoRTs may have played an important role in snoRNA expansion during evolution of the mammalian genome [33-35]. The extensive expansion of snoRNA-encoding genes during mammalian evolution might ensure the presence of a functional copy when a parental gene loses function because of mutation. On the other hand, novel paralogs could independently evolve to generate isoforms with different targets/functions, for example the acquisition of new sites complementary to modification regions of rRNAs [34].

## Conclusions

In the present study, we provide the first experimentally-derived catalog of rhesus monkey ncRNAs. Small nucleolar RNAs (snoRNAs) comprise one of the largest groups of functionally diverse ncRNAs currently known to exist in eukaryotic cells. By performing northern blotting and comparative genomic analysis on rhesus monkey snoRNAs, we determined several features of interest. First, we identified several lineage- or species-specific snoRNAs. Moreover, we observed that the majority of snoRNAs have multiple paralogs in the rhesus monkey genome. Based on the data from the ENSEMBL genome annotation project, the total number of snoRNA-encoding genes was shown to have increased during vertebrate evolution. Our results demonstrate that SINE-like retroposon-mediated *trans*-duplication may have been a driving force for the expansion of novel snoRNAs in the rhesus monkey genome.

## Methods

### Animals and Ethics statement

Two year-old rhesus macaques (*Macaca mulatta*) were used in this study. For tissue sampling, monkeys were anesthetized with ketamine (25 mg/kg) and pentobarbital (30 mg/kg) and killed; tissues were removed, cut into blocks, and immediately frozen in liquid nitrogen for RNA isolation. Murine tissues were collected from six-month-old C57BL/6 mice. All experimental procedures were conducted in accordance with the protocols of the Chinese Academy of Medical Sciences and the Institutional Animal Care and Use Committee of Peking Union Medical College. Chicken tissues were collected from four week-old meat-type broilers (bred by a commercial company, Arbor Acres), in accordance with the policies of the Animal Care and Use Committee of China Agricultural University. Total RNA from human tissues was purchased from Shang Hai Haoran Biological Technology Co. Ltd., Shanghai, China.

### Construction of rhesus monkey libraries enriched in ncRNAcDNA

Total RNA was isolated from mixed heart and skeletal muscle tissue of rhesus macaques. Full-length ncRNA-specific libraries of both capped and uncapped transcripts were generated according to a previously described method [31], with modifications. Total RNA was fractionated on Qiagen-tips with 0.6~1.0 M NaCl gradient elution employing QRW2 buffer (the protocol was taken from the Qiagen RNA/DNA handbook). Highly abundant rRNAs (5.8S rRNAs and 5S rRNAs) and snRNAs (U1 snRNA, U2 snRNA, U4 snRNA, and U5 snRNA) were removed from the small RNA fraction (50~500 nt) employing an Ambion MicrobExpress kit. The remaining RNAs were dephosphorylated with calf intestine alkaline phosphatase (Fermentas) and ligated to a 3' adaptor with T4 RNA ligase (Fermentas). After removal of excess 3' adaptor, the ligation products were split into two aliquots, of which one was treated with PolyNucleotide Kinase (PNK, Fermentas) to phosphorylate non-capped RNA, and the other was incubated with Tobacco Acid Pyrophosphatase (TAP, Epicentre) to remove 5'-end methyl-guanosine caps from capped RNA. Thereafter, both samples were ligated to the 5' adaptor and reverse transcribed with Thermoscript reverse transcriptase (RT) (Invitrogen) using oligo 3RT as the RT primer. cDNA was amplified by PCR over 13 cycles using Platinum Taq (Invitrogen) with the 3RT and 5AD primers, cloned into the vector pGEM-T, (Promega), and sequenced. All primer sequences used in this study are shown in Additional File 9.

### Northern blot hybridization

Total RNA extracted from six rhesus monkey tissues (heart, liver, brain, kidney, spleen, and skeletal muscle), and skeletal muscle from human, mouse, and chicken, were separated by 8% (w/v) PAGE (with 7M urea) and transferred to nylon membranes (N+, Amersham). Probes detecting specific ncRNAs were labelled with digoxigenin (DIG)-11-UTP by *in vitro *transcription using T7 and SP6 RNA polymerase. The RNA blots were hybridized in ULTRAhyb (Ambion) at 68°C overnight, washed with 2 × SSC/0.1% (w/v) SDS washing buffer at 68°C for 2 × 5 min, followed by stringent washing with 0.1 × SSC/0.1% (w/v) SDS buffer at 68°C for 2 × 30 min. Thereafter, RNA blots were blocked with blocking buffer for 30~60 min at room temperature and incubated for 30 min with anti-DIG-alkaline phosphatase (AP) antibody (1:10,000, diluted in blocking buffer). Hybridization signals were detected using the CDP-star reagent (Roche). Chemiluminescent signals were detected on X-ray film.

### Rhesus monkey ncRNA annotation

A total of 4,844 clones were sequenced from the rhesus monkey ncRNA libraries. The Staden package was used to trim vector and adaptor sequences, employing default parameters, and we obtained 4,059 insert sequences for further analysis. After removing redundant sequences, the remaining 2,164 unique sequences were annotated according to their degree of similarity to data in the NCBI nt database (2008-06 release), Rfam ncRNA sequences (8.1), ENSEMBL rhesus monkey ncRNAs and cDNA sequences (release 49), and NCBI rhesus monkey Refseq mRNAs (release 2008-05), using BLASTN (version 2.2.17). We filtered the alignments and retained only those with plus/plus strand matches and e-values above 1e-20. Sequence annotations from these alignments were combined in the priority: Rfam ncRNAs, NCBI nt sequences, ENSEMBL ncRNAs, NCBI refseq mRNAs, and ENSEMBL cDNA sequences. Structural alignment with known snoRNAs was performed using INFERNAL software [57]. SnoReport software [58] was utilized to recognize two major classes of snoRNAs (H/ACA box- and C/D box-containing snoRNAs).

### Target prediction of rhesus monkey snoRNAs

We downloaded sequences and annotations of rhesus tRNAs, rRNAs, snRNAs, and snoRNAs from the GtRNAdb and ENSEMBL databases [6,59]. The guide sequences of C/D box snoRNA were defined by the region sandwiched by the C(C') box and D(D') box. Alignment between snoRNAs and the above mentioned RNA sequences was achieved using a modified BLASTN program. For each guide sequence of C/D box snoRNA, we selected one best-aligned target. The secondary structure of H/ACA box snoRNA was predicted using Mfold software [60]. The guide sequences of H/ACA box snoRNA were identified as sequences within the internal loop of one (or both) snoRNA hairpin structures. We predicted target RNAs for H/ACA box snoRNAs by the following criteria. First, the target RNA should share at least seven nucleotides complementary in sequence to the flanking sequences of the junction sites between the stem and loop of the snoRNA guide sequence, and, second, the predicted pseudouridine site in the target RNA that paired to the 5' nucleotides of juncture sites in guide sequences should be a uridine.

### Comparative genomic analysis of rhesus monkey snoRNAs

Genomic sequences of all examined species were downloaded from the UCSC genome browser [61], together with the genome annotations of ENSEMBL release 50 [6]. The sequences, annotations, and genomic loci of vertebrate snoRNAs were originally predicted by INFERNAL software [57], supported by the Rfam database [7], and were next integrated into ENSEMBL [6]. Conservation of rhesus monkey snoRNAs in human, mouse, and chicken genomes was examined using BLAST. Conservation scores were calculated based on the maximal alignment length and the identity of BLAST hits in each genome. Multi-alignment patterns for snoRNA sequence comparison among different primates were extracted from UCSC Hg18 alignment data after rhesus monkey snoRNA locations were converted to human genome positions employing the UCSC liftOver software. The genomic context, and annotations of protein-coding genes and their orthologs in other species, were downloaded using BioMart, employing the ENSEMBL genome annotation version described above [62]. RepeatMasker [40] and CENSER [63] were used to search for simple repeats and transposons with known sequences. To locate low copy-number snoRNAs, we wrote PERL scripts to search for 5~50 bp repeats in the flanking sequences of rhesus monkey snoRNAs. To find interspersed high copy-number snoRNAs, we used ClustalW [64] and MEGA [65] software to search for consensus sequences in flanking regions within a 10 kb window of the gene of interest.

## Authors' contributions

YZ designed and performed the experiments and drafted the manuscript. JL carried out bioinformatics analysis and participated in manuscript preparation. CJ was responsible for animal care and tissue sampling. TL, JW, and YC participated in bioinformatics analysis. RW and XZ carried out experiments. RC, XJW, and DZ conceived of the study, participated in design and coordination, and helped to draft the manuscript. All authors read and approved the final manuscript.

## Supplementary Material

Additional file 1 **The sequences of 117 rhesus monkey ncRNAs**. In this file, the nucleotide sequences of 117 monkey ncRNAs are provided. The C/C' boxes, D/D' boxes, and guide sequences of C/D box snoRNAs, are highlighted. The sequences of all ncRNAs obtained in this study have been submitted to GenBank (Accession numbers: FJ915946-FJ916062). Click here for file

Additional file 2 **Structure of H/ACA box snoRNAs**. The secondary structures of H/ACA box snoRNAs were predicted using Mfold software. Click here for file

Additional file 3 **Target prediction of C/D box snoRNAs**. The alignments of guide sequences with target sequences are shown. Click here for file

Additional file 4 **Target prediction of H/ACA box snoRNAs**. The guide sequences of H/ACA box snoRNAs were identified within the internal loop(s) of one (or both) hairpin structures. "p5" refers to guide sequences located in the 5'-end hairpin structures of snoRNA; "p3" refers to those in the 3'-end hairpins. The two nucleotides at the junction sites between the stem and the loop in guide sequences are shown in lower case. The predicted pseudouridine sites are denoted by lower case "u". Click here for file

Additional file 5 **The expression patterns of rhesus monkey ncRNAs**. The expression pattern of each ncRNA was examined by northern blotting using total RNA from rhesus monkey spleen, brain, kidney, liver, heart, and skeletal muscle. Samples of total RNA from human, mouse, and chicken skeletal muscle were included in each blot to test the possible expression of ncRNAs in different species. Based on the cumulative northern blotting data, expression patterns in different species can be classified into six types. All northern blot data are shown in this file. Click here for file

Additional file 6 **Sequence alignments of five rhesus monkey snoRNAs in nine primate species**. Multiple alignments of five snoRNAs in nine primate species are shown. The species analyzed were *Homo sapiens *(hg18), *Pan troglodytes *(panTro2), *Gorilla gorilla *(gorGor1), *Pongo pygmaeus abelii *(ponAbe2), *Macaca mulatta *(the rhesus monkey) (rheMac2), *Callithrix jacchus *(calJac1), *Tarsius syrichta *(tarSyr1), *Microcebus murinus *(micMur1), and *Otolemur garnetti *(otoGar1).
Click here for file

Additional file 7 **Copy numbers of 58 rhesus monkey snoRNA families in eight representative vertebrate genomes**. The homologs of 58 rhesus monkey snoRNA families in seven other representative vertebrate genomes were analyzed based on the annotations of ENSEMBL Release 50. In each tested vertebrate genome, the copy numbers of intron-encoded and intergenic snoRNAs were separately calculated. Click here for file

Additional file 8 **Twenty-two intronic snoRNAs with SINE-like retro-transposable elements**. The sequences and features of 22 potential rhesus monkey snoRNA retrogenes were demonstrated. Click here for file

Additional file 9 **All oligonucleotide sequences used in this study**. All oligonucleotide sequences used in this study were shown, which included the sequences for adapters, primers and probes. Click here for file
