# Supplementary material for: Systematic identification and evolutionary features of rhesus monkey small nucleolar RNAs
Source: BMC Genomics. 2010 Jan 25;11:61. doi: 10.1186/1471-2164-11-61 (PMC2832892; doi:10.1186/1471-2164-11-61)
Supplement: Additional file 1 — The sequences of 117 rhesus monkey ncRNAs. In this file, the nucleotide sequences of 117 monkey ncRNAs are provided. The C/C' boxes, D/D' boxes, and guide sequences of C/D box snoRNAs, are highlighted. The sequences of all ncRNAs obtained in this study have been submitted to GenBank (Accession numbers: FJ915946-FJ916062). [file 1471-2164-11-61-S1.pdf]

Additional file 1

The sequences of 117 ncRNAs of rhesus monkey

117 ncRNAs of rhesus monkey include the following types of ncRNAs:

- 32 C/D box snoRNAs
- 48 H/ACA box snoRNAs
- 1 7SK RNA
- 2 RNase
- 2 7SL RNAs
- 1 Vault RNA
- 17 snRNAs
- 6 Y RNAs
- 8 unclassified ncRNA candidates

32 C/D box snoRNAs

The sequences of box C or box C' in each C/D box snoRNA are given in yellow bold

The sequences of box D or box D' in each C/D box snoRNA are given in green bold

The guide sequences of each C/D box snoRNA are highlighted in cyan underline

>SNORD116

ATCGA**TGATGA**GTCCCCCATAAAAACATTCCTTGGAAAAG**CTGA**ACAAAA**TGAGTGA**GAACTCATACCGTCGTTCTCA
TCGGAA**CTGA**GG

>SNORD13

GATCCTTTTGTGGT**TCATGA**GCA**TGATGA**TTGGGTGTTCACACGTTTGTGTGAGATGTGCCACCCTACAACCTTGTTAC
GACGTCGGCACATTCCCCAT**CTGA**C**CTGA**

>SNORD15

TTCAG**TGATGA**CA**TGATGA**CGAGTCAGAAAGGTCACCTC**CTGC**TCTTGGTCCTTGTCAGTACCATGTTCTGTGGTGCT
GTGCGTGAGTTCCTTCTGCAGAAGTGTCCTATTCAT**TGATTGA**TTTAGAGGCATTTGT**CTGA**GA

>SNORD16

A**TGATG**TCGTAATTTGCGTCTTACT**CTGT**TCTCAGCGACAGTTGCCTGCTGTCAGTAAGCTGGTACAGAAGGT**TGACG**
AAAATTTTTA**CTGA**GC

>SNORD17a

GTGAAA**TGATGA**TTCAGTTTATCCATTCG**CTGA**GTGCGCTGCACTGACCTCCTTCCAAGCCTCAGTTCCTGTTCTAGGA
ACTTGAGGCTATGTAGCCTGCAAATGCCCTGCAGTCTGCAGTGTTCTACTGTGAACTGCTTGTGTGTTGGCAGGCTACC
GGTAAGAATGGTTGGTGTCAGCAGGGATGGGGCTCTCTGGGACCCATCTCACAAAGA**TGAGTGGTGA**AAATCTGATCAC
TTG**CTGCA**GCCTT

>SNORD17b

TCGTACGACTCTTAGCGGTGGATTGAAA**TGATGA**TTCAGTTTATCCATTCG**CTGA**GTGCGCTGCACTGACCTCCTTCCA

AGCCTCAGTTCCTGTTCTAGGAACTTGAGGCTATGTAGCCTGCAAATGCCCTGCAGTCTGCAGTGTTCTACTGTGAACT
GCTTGTGTGTTGGCAGGCTACCGGTAAGAATGGTTGGTGTCAGCAGGGATGGGGCTCTCTGGGACCCATCTCACAAAGA
TGAGTGGTGAAAATCTGATC

>SNORD22
TTCCCAATGAAGAAACTTTCACACGTCTTACTCTCTGTCCTAGTCCCAGAGCCTGTAAAGGTGAACCCACTGGGACTGG
CTGGGGGAAAAGAGGAAGATTTGTTCCAGAAGGAACTGTCTGAGGGA

>SNORD24
GATGACAGTTTATTTGCTACTCTTGACTGCTAGAATGATGAGGAGCTTAACCACCATTATCTTAACTGAGG

>SNORD26
GCCAACACTAGCCCGAAGCATAACCAACACTAATACCAAACCAACATATACCAAACCATTCACCTACCTACGGGGATGA
TTTTACAAACTGAACTCTCTCTTTCTGATGGATTAGTGGAGAAAACAAAAATTCTGAGTA

>SNORD27
ACTCCGTGATGAACACAAAATGACAAGCATATGGCTGAACTTATAAGTGATGTCATCTTACTACTGAGA

>SNORD45
GGTCAATGATGAGCTGGCATGTATTCTGAATCTAAAGTTGATTATTAATACTTTAGCTCTAGAATTACTCTGAGACC

>SNORD46
GTAAGGTGATGAAAAAGAATCCTTAGGCGTGGTTGTGGCCGTCTTGGTCACCTGTGTGCCACTTGCCAATGCAAGG
ACTTGTCATAGTTACACTGACTGTT

>SNORD67
GAGAGTGATGAGTTGCACACTGGTGGAGCCATGGTATCAGGTGATACAGGCACCACTCAGTATCACCCTGGTGACAAA
ATCAAGTGCACAGGGGCCATCTGATT

>SNORD87
CACAGTGATGACTTAAATTACTTTTTGCCGTTTACCCAGCTGAGGGTGTCTTTGAAGAAATAATTTTAAGACTGAGA

>SNORD94
AGGCTGTGATGATTGGCGCAGGGGTACGGACCTCAGCTGAGTCATGGGAGCTGAATGTATGTGTTTCTCTTTTGTCCT
GCGTGTGGCAGGCTAATGGGGAGCACTTACATGAGACTGTTGCCTCAATCTGAGCCT

>snosnR60_Z15
ATGATGATAACATAGTTCAGCAGACTAACGCTGATGAGCAATATTAAGTCTTTCGCTCCTATCTGATG

>snoU6-53
TCCCAATGATGAGTTGCCATGCTAATACTGAGCCACCAGGTAGGGCAGTGTTGCCCTGGTTTGGGTGCCAGTGAGTTT
AACAAAACTTCTCACATGAAGATCTGAGGGGC

>U8
ATCGTCAGGTGGTATAATCCTTACCTGTTCCTCTTTTGGAGGGCAGATTAGAACA**TGATGA**TTGGAGAATGCATGAAAC
G**TGATTA**ACGTCTCTGCGTAATCAGGACTTGCAACACC**CTGA**TTGCTCCTAT**CTGA**TT

>U3a
CCGAAAACCACGA**GGAAGA**GACGTAGTGTTCTTTC**CTGA**GCGTGAAGCCGGCATTTGGTGTTGCTTTACTGCAACC
GCCGTTTGCCAT**TGATGA**TCGTTCTTCTCTCCTTTTTTGGGGAGTAAGAGGGAAAGAACACGGT**CTGA**GTGG

>U3b
AACACTATACTTTCAGGGATCATTTCTATAGTTTGTTACTAGAGAAGTTTCTCTGAATGTGTAGAGCACCGAAAACCAC
GAGGAAGAGACGTAGTGTTCTTTCCTGAGCGTGAAGCCGGCATTTGGTGTTGCTTCACTACAACCGCTATTTGCCGT**TG**
ATGATCGTTCTTCTCTCCATTTTTGGGGAGTAAGAGGGAAAGAACACGGT**CTGA**GTGGT

>U3c
AAAGACTATACTTTCAGGGATCATTTCTATAGTTTGTTACTAGAGAAGTTTCTCTGAATGTGTAGAGCACCGAAAACCA
CGAGGAAGAGACGTAGTGTTCTTTCCTGAGCGTGAAGTCGGCATTTGGTGTTGCTTCACTGCAACCGCTATTTGCCAT**T**
GATGATCGTTCTTCTCTCCATTTTTGGGGAGTAAGAGGGAAAGAACACGGT**CTGA**GTGGT

>U3d
AAAAGACTATACTTTCAGGGATCATTTCTATAGTT**TGTGA**CTAGAGAAGTTTCT**CTGA**ACGTGTAGAGCACCGAAAACC
ACGAGGAAGAGACGTAGTGTTCTCTTCTGAGCGTGAAGCAGGCGTTTGGTGTTGCTTCCGTGCAACTGCCATCAGCCAT
TGATGATCGTTCTTCTCTCCGCTTTGGAGAGTAAGAGGGAGAGAACGCTGT**CTGA**GTGGT

>U3e
AAAAAACTATACTTTCAGGGATCATTTCTATAGTT**TGTGA**CTAGAGAAGTTTCT**CTGA**ACGTGTAGAGCACCGAAAACC
ACGAGGAAGAGACGTAGTGTTCTTTCCTGAGCGTGAAGTCGGCATTTGGTGTTGCTTCACTGCAACCGCTATTTGCCAT
TGATGATCGTTCTTCTCTCCATTTTTGGGGAGTAAGAGGGAAAGAACACGGT**CTGA**GTGGT

>U3f
AAAAAAAAACTATACTTTCAGGGATCATTTCTATAGTT**TGTGA**CTAGAGAAGTTTCT**CTGA**ACGTGTAGAGCACCGAAA
ACCACGAGGAAGAGACGTAGTGTTCTCTCCTGAGCGTGAAGCAGGCGTTATGGTGTTGCTTCTGTGCAATTGCCATCAG
CCAT**TGATGA**TCGTTCTTCTCTCCGCTTTGGAGAGTAAGAGGGAGAGAACGCTGT**CTGA**GTGG

>U3g
AAGACTATACTTTCAGGGATCATTTCTATAGTTTGTTACTAGAGAAGTTTCTCTGAATGTGTAGAGCACCGGAAACCAC
GAGGAAGAGACGTAGTGTTCTTTCCTGAGCGTGAAGCCGACATTTGGTGTTGCTTCACTGCAACCGCCATTTGCCAT**TG**
ATGATCGTTCTTCTCTCCGCTTTGGAGAGTAAGAGGGAGAGAACGCTGT**CTGA**GTGGT

>U3h
AAGACTATACTTTCAGGGATCATTTCTATAGTT**TGTGA**CTAGAGAAGTTTCT**CTGA**ACGTGTAGAGCACCGAAAACCAC
GAGGAAGAGACGTAGTGTTCTCTTCTGAGCGTGAAGCAGGCGTTTGGTGTTGCTTCCGTGCAACTGCCATCAGCCAT**TG**
ATGATCGTTCTTCTCTCCATTTTTGGGGAGTAAGAGGGAAAGAACACGGT**CTGA**GTGG

>U3i
AAGACTATACTTTCAGGGATCATTTCTATAGTTTGTTACTAGAGAAGTTTCTCTGAATGTGTAGAGCACCGAAAACCAC
GAGGAAGAGACGTAGTGTTCTTTCCTGAGCGTGAAGTCGGCATTTGGTGTTGCTTCACTGCAACCGCTATTTGCCAT**TG**
ATGATCGTTCTTCTCTCCGCTTTGGAGAGTAAGAGGGAGAGAACGCTGT**CTGA**GTGGT

>U3j
AAGACTATACTTTCAGGGATCATTTCTATAGTT**TGTGA**CTAGAGAAGTTTCT**CTGA**ACGTGTAGAGCACCCAAAACCAC
GAGGAAGAGACGTAGTGTTCTCTCCTGAGCGTGAAGCAGGCGTTTGGTGTTGCTTCCGTGCAACTGCCATCAGCCAC**TG**
ATGATCGTTCTTCTCTCCGCTTTGGAGAGTAAGAGGGAGAGAACGCTGT**ATGA**GTGGT

>U3k
AAGACTATACTTTCAGGGATCATTTTTATAGTTTGTTACTAGAGAAGTTTCTCTGAATGTGTAGAGCACCGAAAACCAC
GAGGAAGAGACGTAGTGTTCTTTCCTGAGCGTGAAGCCGGCATTTGGTGTTGCTTCACTGCAACCGCCATTTGCCAT**TG**
ATGATCGTTCTTCTCTCCAGTTTTGGGGAGTAAGAGGGAAAGAACACGGT**CTGA**GTGGT

>U3l
TAAGACTATACTTTCAGGGATCATTTCTATAGTTTGTTACTAGAGAAGTTTCTCTGAATGTGTAGAGCACCGAAAACCA
CGAGGAAGAGACATAGTGTTCTTTCCTGAGCGTGAAGCCGGCATTTGGTGTTGCTTCACTGCAACCGCCATTTGCCAT**T**
GATGATCGTTCTTCTCTCCGCTTTGGAGAGTAAGAGGGAGAGAACGCTGT**CTGA**GTGGT

>U3m
AAGACTATACTTTCAGGGATCATTTCTATAGTTTGTTACTAGAGAAGTTTCTCTGAATGTGTAGAGCACCGAAAACCAC
GAGGAAGAGACATAGTGTTCTTTCCTGAGCGTGAAGCCGGCATTTGGTGTTGCCTCACTGCAACCGCCATTTGCCAT**TG**
ATGATCGTTCTTCTCTCCATTTTTAGAGAGTAAGAGGGAAAGAACACGGT**CTGA**GTGGT

>U3n
AAGACTATACTTTCAGGGATCATTTCTATAGTTTGTTACTAGAGAAGTTTCTCTGAATGTGTAGAGCACCGGAAACCAC
GAGGAAGAGACGTAGTGTTCTTTCCTGAGCGTGAAGCCGACATTTGGTGTTGCTTCACTGCAACCGCCATTTGCCAT**TG**
ATGATCGTTCTTCTCTCCATTTTTGGGGAGTAAGAGGGAAAGAATACGGT**CTGA**GTGGT

48 H/ACA box snoRNAs

>SCARNA11
TTTGTCCTGGCCTATTTTTCTGCTCCCCTGTGCTCAGTTCTAACAGGGTAGTCTGGCAGGACACACAGTAATTTCCCT
CTCAGTTTAGGAGGGCCGTCCTAAGAATGGGGCTGGCTCTTAAAGGCACGAGAGGACAGTTTAAAA

>SCARNA15
CTGGAGACTAAGAAAATAGAGTCCTTGAAATCAAGCTGACTCTGCTTTTAGCCTCCTAAATGAAAAGGTAGATAGAA
CAGGTCTTGTTTGCAAAATAAACTCAAGGCCTACTTATCTACCAACAGCA

>SCARNA25
ATGGAAGCTGCAGGAACCAACCATGTGGACAAATGAAAAAATGCCTGTTGTCTACAGATTGCAGCGATCCTACATA
AACATATGGAGGTCTCTGTCTGGCTTAGGACAGCTGGCTAAGTCTGATCGTTCCCCTCCATACAAC

>SCARNA4
CTGGAGGACTAAGAAGGCTGAGTCTGATGAAGCAAGACTTTGCTGATACATTCCTCCTAGAAAAAAGGGTTGGAGA
GAGCAGCCTTCACTGAAGAGTATCACAGGGCTGACTGTACTACCCAACACTC

>SNORA11
TGGGGTGTGCTCAGAGCAGGGGGCGCGAAGAATGCCTCCTCTGTTTACAACACACCCAACAGTAATCTGGGGTCAT
TGTGACAAGGGACACAGAGCTTGTGACCTCCCTACAAAC

>SNORA13
TCAGCCTTTGTGTTGCCCATTCACTTTGGAAACTAGTGAATGTGGTGTCAAAAAAGACGTAAATTAAACGCTTTGCA
GCCTTTTCCTGCCCTTGAATTTGGTATCTTTGGTGTAGGAGCTGCATAAGTAACAGTT

>SNORA14
CTGCATTCTTAAACCCTCTTGGTAGCTTCGTTCTAAGTGCTTCCAAGATATGAGTGAATGCTATAGAAATTGCAGGGG
AGTCCAAAGGGCTGCGCTTCTCCCGTGGCTCAGTCTTATTTCATACCTGCGACA

>SNORA15
AAGGCATGGCCGAATACTGTGTTCTTTTATCAGTAGTTTACACAGCCAGACACCATGCAAAAGCAGTCTTCCCTTTG
GAATGACCAATGGTATGCTAAGGTTTTTCATAGCATATCATTATTAAAGGTGAATACAGAT

>SNORA17
CACTGCCCCTAGAGGCGTTGCAGCTGTGGCTGCCGTGTCACATCTGTGTCATTAGGTGGCAGAGATTAGAGAGGCTA
TGTCTACGCTCGGCGTTCTGCCCCGTGAACGTTTGAATGTTTGATAGTCTGACACTC

>SNORA18
GGTTGAGGTCTATCCCGATGGGGCTTTTCCTGTAGCCTGCACGTCGTTGGAAACGCCTCATAGAGTAACTCTGTGGT
TTTACTTTACTCACAGGACTATTGTTAGGTCTGTGGGAAGGAACTACAAGACAGTT

>SNORA19
GTGCACATGTCATTGACCTGCTTTCTTCTGTATATGTGAGCAGTGTTATTTCTTATGTGCTATACAAATAATTGAAGGC
TAATTAGCAGTATAACTATAAAAAGTAATGCTGCCAGTCTCCTTCAGACAAAA

>SNORA2
TTGGCCCTGAATCAAGGCCAGCAGTTTGCTGAAGCTGTTGGTTTCAAGCAGGAGCCTAAAGAATTGTCTTTCTGTG
GTCTGTTGGCCATTTCATAAGTTCGGAAATGTAATGGTCAATTCATTAGAAAGAAACATGA

>SNORA20
TCTTCCCATTTATTTGCTGCTTATAGTCGCACAGTGATATGAGCAGTTATACGCGTGGGATAAAATAACATTGGGTCAC
TGTGAATTGAGATGAAGTAACCATTTTCATCTCTTCTGCATAGACTAGACATTG

>SNORA23
CATGGCTGCCGTAATGTGTGCATAGGTTTCATCTGTGTCTGGTAGCAGTGTCTGTCTGTGTTTTGCATTCAGATCTTGC
TATCCACACAAACGTCATGCAGCCAAAGAGTAACTTGGGATCATAGTACTGGTCTAGTGTTGTCTCTGGACACATCT
ACCACTGGCCAGCCTCCAAATTCCCACACACAG

>SNORA24
CTCCATGTATCTTTGGGACCTGTCAGCTGTGGCAGTCTCCCTTCCTAGCCATGGAAGAGCATATTCTTGTTTATTGGC
AAAGCTGTCACCATTTAATTGGTATCAGATTCTGACTTGCACAAGTAACATT

>SNORA25
TAGGTCATTTCAAAGAGGGCTCATGAGGCTGTGAAACCCAGAGCTCTTAACGCTGTGACCAAAGATGGAAGTTCTC
TATAGGATGCCATAGCACTCCTATGTTTGGTGCTATGTTTTCCTGAGGAGATATAAA

>SNORA27
TACCCCCTTTTCACTTTGCCAATTGGACTTATGTCTTTATTGGTCATTCAAGTGGGGCAAAGGAAATAATCCTTTTAAA
ACTCAGGCAAACTGAGTGTTTGTCTTGTATCCTGTCAGAGTAAACAAAT

>SNORA28
TTGCAAGCAACACTCTGTGGCAGATGATGGAAACTGTCTGACACAATTTGAGCTTGCTATAGCAAGAAAGTCTAAC
CTATTCCGGTGTTCTCTCTCCCATGAGACAAGCCGTTATATAGACTTAAACAG

>SNORA31
CTGCATCCACTGATAGACCTTGAACAATTTACTGTTGTTCTTTTGGTTTGCACTAGGATGCAAAAGAAAAATCCCTGC
GCTTTCTGTCTGTCTTTGTGGCGGCCCAGATTGAATTGGGGAATACATCT

>SNORA36a
ATTCCAAAGTGTTGAGTTCAGTTCAGGGTGGCTTCCCTGCTCTGTTAATTAAACTTTGGAACATTGAAACTGGCTAG
GGAAAATGATTGGATAGAAAATGTTATTCTATTCATTTATCCCCAGCCTACAAAA

>SNORA36b
TTCCAAAGTGTTGAGTTCAGTCCAGGGCAGCTTCCCTGTTCTGTTAATTAAACTTTGGGACATTAAAATGGACTAAG
GGAGATGATTGGGTAGGAAGTATTATTCTATTCATTTGCCTCCCAGCCTACAAAA

>SNORA4
CTACCAAAAGTTAGCTTTTTGGGGGGAAGGTTTTTAAGTAACCTTTGCCAGCTTGGGCTATTTGGAAGAGTAAAAGG
ACCACACTCCACAGTGGGCTATACCACTTAGTATAGTTCGCTACTATTTTGTGGCCTACATGA

>SNORA40
CTGCACTTATGTATGTTTTTGTTTAACTTGTGGACAAAGACTTATAAATAGGTGCAAAAAATAAATCCTCTTTTGCAA
CCCAGAACTCATTGTTCAGTATGAGTTTTGATACATATAAGAAGGGATATTA

>SNORA41
TTCCACAGCTACTGGTCTGCAGCTGTTCTTATGGTAGCAGTTGTGGCATTCCTCTGTGGGAAAGAAACTGTTAACAC
AAACACCTCTTTCTTAGCAAAACAGAAAGTGGGCATATGTGTGACAGACATAAG

>SNORA42
GAGCATGGTAATGGATTTATGGTGGGTCCTTCGCTGTGGGCCTCTCATAGTGTACCCATGCCAGAGTAAATGGCAGCC
TCAAACCATTGCCCAGCCCCCTTACCTGTGGGCTGTGAGCACTGAAGGGGGTTGCACAGTG

>SNORA43
GCTGTCCTGGACCTGTCGGCACCAGACAGTTGCTCTGCCGTGCCTGTGACCTCGGGGCAAAAAGGAAGTGGCGATT
TCTACACTCAGTGCCCGGGAACCAGTGGGCACTGAGAATGGTTTATGGCCTGACATGA

>SNORA49
GCTTCCTCAGCCTTACTCCAGGGGCTTTTTGTTGCCTGTAAAGTGCCCTGGCATTGCCTGAGGATAGATGAGAAAGC
ACATGTCCCTCCCCAGTAAGACGCTGTTTTCTTTTGGGGCCTACAAGTTGAGCTGACAGTA

>SNORA5
CTGCAGCCATGTCAAATTCAGTGCCTGTCCTATACATGGTAGGCACTGGCCCAGAAGGCTGCCACAGAAACACTGT
GACTCATGGGCCCTGTTCCTGGGTCCCAGGCTCAGGGATAAATTTGGTTACAGACATCA

>SNORA50
AAGCACTGCCTTTGAACCTGATGTGTCTTGTTTGTAGCTTCACGGGCCAAGCAACAGTGCTAGAGCATAACGACTTG
TTATAACTGGGGCTCTTCAGCTCTCAACTGAACTGCTCTTTTAAAAACAAGGTACATTT

>SNORA53
CAACATGCTTCCTTAGATCCACCTTTGTGGATGAATCTTGAACTGAGTTCCACTTGTAAACTTCTTGTTTCTTGTGGT
TCCAGTCAAAGAAACATCCAGCAACTTTTTTGGTTGTATAGTCAAAGGTGCTTGAGTCATTGGCATGTAAGAGAAAT
ATACCTGCATGTTAGTCTAACGTTCTGATAGAAATGACATGCATTTTTGCTGCCATTTGTTACTATCAGGACTCGACTC
GTGTGCGGACACTT

>SNORA54
GAGCACTGTTCGTTAACCTGTTAGCCTGGCTGTAGCTAATGGGTTCCATTCCAGTGCAATAGCATTTCCAGCGACACA
TGACTGACTGACTGGTGGCTTTCAGTTTCAGGTCTTGGAGACAAAT

>SNORA58
GGGCATACCCGTAGACCTTGCCTGACTGTGCTCATGGCCAGGCAAGGGGGACAGTGTATGCAAGAGTAATGTGGAG
TTTGTGCTAACTCTAGCCAGCTTAATTAATGACTGGATAAATTGCACAACTCTCACATTCT

>SNORA61
AATCCTCCTGTTCCCTTTCCCATCGGATCTGAACACCGGTCTTGGTGGTCGTAAAAGGAGGAAAAGTAATAGTGAAG
CTGGCCTGAATGTTGTAATCTGGTATATGGCATGTGGGCTAGTTTCAGACAGGT

>SNORA62
CTGCACACTATTAAAGCTCAGGGTGGAGGCCAGTCTTGGCTCATGAACTTCTGAGTGTCGGAAGTGTGCTACATTAA
TGGCAGGATTTTCGCTAACACCAGTAGAGCTTGCCTCTATGACTGGAGTTTGGTAGTACTCGCTGCCACATAC

>SNORA63
AAAGCAGGATTCAGACTACAATATAGCTGCTAAGTGCTGTGTTGTCGTTCCCCCTGCTCAAAATAAAGTTGTTTCTTA
ACTATACCTGTCTGCTATATCCCTGTAGCAGCCAGGGACGCTTGGTCTCATACATGT

>SNORA64
GTCTCTCAGCTCCGCTTAACCACACGGGTCCGGCGTGTGCTTGGCGTGTTTTCAGGGAGGCAGAGAAAGGCTCTCC
TAATGCACGACAGACCCGCCCAGCATGGCCTCTCTGTTCCTAGGAGTGCGACAGAC

>SNORA66
GTGCAAACTCGATCACTAGCTCTGCATGATGTGGCAGAAGCGAAGGGAACCAGGTTTGCAAAAGTAACTGTGGTGA
TAGAAATGTGTTAGCCTCAGACACTACTGAGGTGGCTTTCTATCCTAGTACAGTC

>SNORA68
ATTGCACCTAAACCCAAGAATCACTGTTTCTTATAGCGGTGGTTTAAACAGAGGTGCAAACAGCAAGCGGATCTCGT
CGCCTTTGGGAGGCTGTGGCTGTCCCCCTCAAAGTGAATTTGGAGGTTCTACAACT

>SNORA7
AACCTCCTGGGATCGCGTCTGGAGAGTGCCTAGTATTCTGCCAGCTTCGGAAAGGGAGGGAAAGCAAGACTGGCA
GAGGCACCCATTCCATTCCCAGCTTGCTCCGTAGCTGGCGATTGGAAGACACTCTGCGACAGTGA

>SNORA70
CTGCAGCCAATTAAGCCGACTGAGTTCCTTTCCTCATGGGGGCCCAGTGTGCAATGACTGCAAACAGCAGCTTCCTT
GGTAGTGTATGCAGCCTGTTTGTTGTATGGGTTGCTCTAAGGGACCTTGGAGACAGGC

>SNORA71
CACCTGCATTCAAAAATGATCACGGGCTGCCTGTGCTCTGGTCATCGATAACGCAGGGAGAGGAATTGCTGAAAGC
CGTTTCCCGTGTTTGGAGGGTTCACACCTGTCCCTTTCAAATGCTGGCGCCTTCACACACTC

>SNORA72
CTGCGAATATTCTCGCTGTTCTGATTTTGTAATAGTCAGGACAGGCTAAACATTCGCTATATTAAGACCATGCGTGTGT
CCCCAGACCTAGTTCTTTCCCTAGGTCTGGTTTTATAAATGCTGGTGATAAAC

>SNORA73
AATCCCTTTCCACAACGTTGGAGATAAAGCTGGGCCTTGAGTCTGCGCCTGCATATTCCTACAGCTTCCCAGAGTCC
TGTGGACAATGACTGGGGAGACAAACCATGCAGGAAACAACC

>SNORA74a
ATCCAGCGGTTGTCAGCTATCCAGGCTTGTGTGGTGCCTGTGATGGTGTTACACTGTTGGAAGAGCAAGCACTGTCT
TTATTGAGGTTTGGCTCCAAGCACTGTTTTGGTGTTGTAGCTGAGAAAATCTCAATA

>SNORA74b
ATCCAGCGGTTGTCAGCTATCCAGGCTTGTGTGGTGCCTGTGATGGTGTTACACTGTTGGAAGAGCAAGCACTGTCT
TTATTGAGGTTTGGCTCCAAGCACTGTTTTGGTGTTGTAGCTGAGTACCTTTGGGCAGTGTTTTGCACCTCTGAGAG
TGGAATGACTCCTCCTGTGGAGTTGGTCCTAGTCTGGGTGCAAACAATT

>SNORA76
ACGCGCTGTCTTTGAGCCCCCGCCGAGCCTCCTCGTGGCGCCGGGGGTCAATCTGCAGCGCTAGAGCATGTGCTTG
CGCATAACTGGGGCCGCTTGGCCCCCCGCGGGCGGCCCTTTTAACCGCGAGCGACAATT

>SNORA8
TTGCACTGCATGGTATCTGCACTCAGCAGTTTACTCCTGCTAGGGTGTTCAAAGGTCAGTGCTATAGAAATTCAGTAT
CTGGCATCGTTGGTTTTCTTGGCTTTGTGCTTGTTAAACCTGGTATTTCTATTGATACAGTA

>SNORA81
TACCTTCTTGTATAAGCACTGTGCTAAAATTGCAGACACTAGGACTATGTCTTGGTTTTTGCAATAATGCTAGCAGAG
TACACACAAGAAGAAAAGTAACAGCACTAGATTGTAAAGACTGGGATGGACCTCTTTCTTAATGTCCAATGTCCTTT
GTCTTAAGATTTGGTGCAATATCT

37 other ncRNAs

>7SK RNA
AGGATGTGAGGGCGATCTGGCTGCGACATCTGTCACCCCATTGATCGCCAGGGTTGATTCGGCTGATCTGGCTGGCT
AGGCGGGTGTCCCCTTCCTCCCTCACCGCTCCATGTGCGTCCCTCCCGAAGCTGCGCGCTCGGTCGAAGAGGACGA
CCATCCCCGCTAGAGGAGCACCGGTCTTCGGTCAAGGGTATACGAGTAGCTGCGCTCCCCTGCTAGAACCTCCAAA
CAAGCTCTCAAGGTCCATTTGTAGGAGAACGTAGGGTAGTCAAGCTTCCAAGACTCCAGACACATCCAAATGAGGC
GCTGCATGTGGCAGTCTGCCTTTCTTT

>RNase_MRP, RNA component of mitochondrial RNA processing endoribonuclease (RMRP)
ATTCCCCCTTCCCACTGTAAAGTCCGCCAAGAAGCGTGGCCCGCTGAGCGGCGTGGTGCGGGGTAGTTGTCCGTCA
GCTCTTCTAGTTACGCAGGCAGTGCGTCTTGGCGCACTAACCACACGGGGCTCATTCTCAGCGCGTCTGG

>RNaseP_nuc, ribonuclease P RNA component H1 (RPPH1)
ATAAGGCGGAGGGAAGCTCATCAGTGGGGCCACGAGCTGAGTGCGTCCTGTCACTCCACTCCCATGTCCCTTCGGA
AGGTCTGAGACTAGGGCCAGAGGCGGTCCTAACAGGGCTCTCCCTGATCTTCGGGGAGGTGAGTTCCCAGAGAATG
GGGCTCCGCGCGAGGTCAGACTGGGCAGGAGATGCCGTGGACCCCGCCCTTCGGGGAGGGGCCCGGCGGATGCCT
CCTTTGCCGGAGCTTGGAACAGACTCACGGCCGGCGAAGTGAGTTCAATGGCTGAGGTGAGGTACCCCGGAGGGG
ACCTCATAACCCAATTCAGACTACTCTCCCCCGCCCAT

>7SLa
GCCGGGCGCGGTGGCGCGTGCCTGTAGTCCCAGCTACTAGGGAGGCTGAGGTGGGAGGATCGCTTGAGCCCAGGA
GTTCTGGGCTGTAGTGCGCTATGCCGATCGGGTGTCCGCACTAAGTTCGGCATCAATATGGTGACCTCCCGGGAGCG
GGGGACCACCAGGTTGCCTAAGGAGGGTGAACCGGCCCAGGTCGGAAACGGAGCAGGTCAAAACTCCCGTGCTGA
TCAGTAGTGGGATCGCGCCTGTGAATAGCCACTGCACTCCAGCCTGGGCAATATAGCGAGACCCTGTCTCTA

>7SLb
GCCGGGCGCGGTGGCGCGTGCCTGTAGTCCCAGCTACTAGGGAGGCTGAGGCTGGAGGATCGCTTGAGTCCAGGA
GTTCTGGGCTGTAGTGCGCTATGCCGATCGGGTGTCCGCACTAAGTTCGGCATCAATATGGTGACCTCCCGGGAGCG
GGGGACCACCAGGTTGCCTAAGGAGGGGTGAACCGGCCCAGGTCGGAAACGGAGCAGGTCAAAACTCCCGTGCT
GATCAGTAGTGGGATCGCGCCTGTGAATAGCCACTGCACTCCAGCCTGGGCAACATAGCGAGACCCTGTCTCTA

>Vault RNA
CTTTAGCTCAGCGGTTACTTCGGCGACACCTCCTAGGATTACACCAACCTCTCTGGGTTGTTCGAGACCCGCGGGCG

CTCTCCAGTCCT

>U1-1
GGCGAAGCTACCATCTGTGGGATTATGACTGAACGAAGGTGGTTTTCCCAGGGCGAGGCTTATCCATTGCACTCCGG
ATGTGCTGACCCCTGCGATTTCCCCAAATGTGGGAAACTCGACTGCATAATTTGTGGTAGTGGGGGACTGCGTTCGC
GCTTTCCCCT

>U1-2
ATACTTACCTGGCAGGGGAGATACCATGATCACGAAGGTGGTTTTCCCAGGGCGAGGCTTATCCATTGCACTCTGGA
TGTGCTGACCCCTGCGATTTCCCCAAATGTGGGAAACTCGACTGCGTAATTTGCGGTATTGGGGGACTGCGTTCGCG
CTTTCCCCTGAA

>U1-3
ATACTTACCTGGCAGGGGAGATACCATGATCACGAAGGTGGTTTTTTCAGGGCGAGGCTTATCCATTGCACTCCGGA
TGTGCTGACCCCTGCGATTTCCCCAAATGTGGGAAACTCGACTGCATAATTTGTGGTAGTGGGGGACTGCGTTCGCG
CTTTCCCCTGAT

>U11
AAAAAAAGGGCTTCTGTCGTGAGTGGCACACGTAGGGCAACTCGATTGCTCTTCGTGCGGAATCGACATCAAGAGA
TTTCGGAAGCATAATTTTTTGACATTCGGGCAGCTGGTGATCGTTGGTCCCGGCGCCCTA

>U12
ATGCCTTAAACTTATGAGTAAGGAAAATAACGATTCGGGGTGACGCCCGAATCCTCACTGCTAATGTGAGACGAATT
TTTGAGCGGGTAAAGGTCGCCCCTAAGGTGACCCGCCTACTTTGCGGGATGCCTGGGAGTTGCGATCTGCCCG

>U2-1
CTTCTCGGCCTTTTGGCTAAGATCAAGTGTAGTATCTGTTCTTATCAGTTTAATATCTGATACGTCCTCTATCCGAGGA
CAATATATTAAATGGATTTTTGGAATTAGGAGATGGAAAAGGAGCTTGCTCCGTCCACTCCACGCATCGACCTGGTAT
TGCAGTACTTCCAGGAACGGTGCACCA

>U2-2
ATCGCTTCTCGGCCTTTTGGCTAAGATCAAGTGTAGTATCTGTTCTTATCAGTTTAATATCTGATACGTCCTCTATCCG
AGGACAATATATTAAATGGATTTTTGGAGCAGGGAGATGGAATAGGAGCTTGCTCCGTCCACTCCACGCATCGACCTG
GTATTGCAGTACCTCCAGGAACGGTGCACCA

>U4
AGCTTTGCGCAGTGGCAGTATCGTAGCCAATGAGGTTTATCCGAGGCGCGATTATTGCTAATTGAAAACTTTTCCCAA
TACCCCGCCATGACGACTTGAAATATAGTCGGCATTGGCAATTTTTGACAGTCTCTACGGAGACTGAC

>U4atac
AAACCATCCTTTTCTTGGGGTTGCGCTACTGTCCAATGAGCGCATAGTGAGGGCAGTACTGCTAACGCCTACACAAC
ACACCCGCATCAACTAAAGCTTTGCTTTACCTTGGTGCAATTTTTGGAAAAATG

>U5-1
TAGCATGATACTCTGGTTTCTCTTCAGATCGTATAAATCTTTCGCCTTTTACTAAAGATTTCCGTGGAGAGGAACAACT
CTGAGTCTTAAACCAATT

>U5-2
ATACTCTGGCTTCTCTTCAGATCGCATAAATCTTTCGCCTTTTACTAAAGATTTCCGTGGAGAGGAACAACTCTGAGT
CTTAACCCAATTTTTTGAGGCCTTGTATTTTGCAAGGCTA

>U5-3
ATATACTCTGGTTTCTCTTCAAATCGTATAAATCTTTCGCCTTTTACTAAAGATTTCCGTGGAGAGGAACAATTCCGAG
TCTTAAGCCAATTTTTTGAGGTCCCACGTTTGTGGGACTAAC

>U5-4
ATACTCTGGTTTCTCTTCAGATCGTATAAATCTTTCGCCTTTTACTAAAGATTTCCGTGGAGAGGAACAACTCTGAGT
CTTAAACCAATTTTTTGAGGCCTTGTTTCGGCAAGGCTACATT

>U5-5
ATACTCTGGTTTCTCTTCAGATCGTATAAATCTTTCGCCTTTTACTAAAGATTTCCGTGGAGAGGAACAACTCTGAGT
TTTAAACCAATTTTTTGAGGCCTTGCTTTTTCAGCAGGGCTTATAA

>U6-1
AATATACTAAAATTGGAACGATACAGAGAAGATTAGCATGGCCCCTGCGCAAGGATGACACGCAAATTCGTGAAGC
GTTCCATATTTTA

>U6-2
AAAAATTGGAACGATACAGAGAAGATTAGCATGGCCCCTGCGCAAGGATGACACGCAAATTCGTGAAGCGTTCCAT
ATTTTTTTTGACCACGAGCTCACAGGGT

>U6atac
GTGTTGTATGAAAGGAGAGAAGGTTAGCACTCCCCTTGACAAGGATGGAAGAGGCCCTCGGGCCTGACAACACGC
ATACGGTTAAGGCATTGCCACCTACTTCGTGGCATCTAACCATCGTTTTTTT

>Y1
GGGAGTAACTATGACGTTATTGTTAAGTTGATTTAACATTGTCTCCCCCCACAACCGCGCTTGACTAGCTTGATGTT

>Y2
ACGAAAAAGTTGGTCCGAGTGTTGTGGGTTATTGTTAAGTTGATTTAACATTGTCTCCCCCCACAACCGCGCTTGAC
TAGCTTGACGTTT

>Y3
GAGGCGGGGGGGCGAGCCCCGAGGGGCTCTTATTGTTAAGTTGATTTAACATTGTCTCCCCCCACAACCGCGCTTGA
CTAGCTTGACGTT

>Y4
GGCTGGTCCGATGGTAGTGGGTTATCAGAACTTATTAACATTAGTGTCACTAAAGTTGGTATACAACCCCCCACTGCT
AAATTTGACTGGCTTTTT

>Y5
AGTTTGGTCCGAGTGCAGTGGTGTTTACAACTAATTGATCACAACCAGTTACAGATTTCTTTGTTCCTTCTCCACTCC
CACTGCTTCACTTGACCAGCCTTA

>Y6
GGCTGGTCCGAAGGTAGTGAGTTATCTCAATTGATTGTTCACAGTCAGTTACAGATCGAACTCCTTGTTCTACTCTTT
CCCCCCTTCTCACTACTGCACTTGACTAGTCTTA

>unclassified ncRNA candidate 1
CTATCGGGCCCATACCCCGAAAATGTTGGTTATATCCTTCCCGTACTACCA

> unclassified ncRNA candidate 2
ACCTGTAGCTGTCTAGCGACAGAGTGGTTCAATTCCACCTTTCGGGCGACA

> unclassified ncRNA candidate 3
TAATACACTGGTCTTGTAAACCAGAAATGAGCACCCTTCCTAGGGCACCCCA

> unclassified ncRNA candidate 4
CACTGTATTGCTATCCCAGCATTAACCTTTTAAGTTAAAGACGGGGACACACCCCCTGCAGTGACCA

> unclassified ncRNA candidate 5
TGTGTTTCAGAAATTAAGTGTTTCTACTTACTAAGGGCTTTGAAGGCTCTTGGTCTTGTTTAACCTAAATTTCTA

> unclassified ncRNA candidate 6
ATGCATCTATTTGACAGACCTGGAGCAGTTCTACTACCTGCTGCTAAGGTTTCCACTACAGATGCAAGAAAAACATG
TCCTTGCGCTTTCCGTCTGTCTAATTTGTGGCAGCTGAGATTGAATAGAGGAATACAGGG

> unclassified ncRNA candidate 7
CTCCAGCTGTAGGCAGCTGCCTAGGTTGTCTTGTACCTAGGCAAGTGTTACACTGCTGGGAGAACAGCAGCCAATA
GCTGGTTGGCATTCTGGCCCTGGTTCATGCCAACTCTGTGTTGACTACCCCAGGATGCCAGCATAGT

> unclassified ncRNA candidate 8
TTTGATGGCTGTTCCTCTCACTGCTTGGAGCCTTAGGCAGTGGGATTTTGATCCATCATATATCAAAAATGGCTTATCT
TCACTCAGGGCACCATGAGGATGGGCTGGCTGTCCGTTAGTGCCTTCTGATTTTTGCGGAGTCAAACAATTACT
