# Supplementary material for: Systematic identification and evolutionary features of rhesus monkey small nucleolar RNAs
Source: BMC Genomics. 2010 Jan 25;11:61. doi: 10.1186/1471-2164-11-61 (PMC2832892; doi:10.1186/1471-2164-11-61)
Supplement: Additional file 2 — Structure of H/ACA box snoRNAs. The secondary structures of H/ACA box snoRNAs were predicted using Mfold software. [file 1471-2164-11-61-S2.pdf]

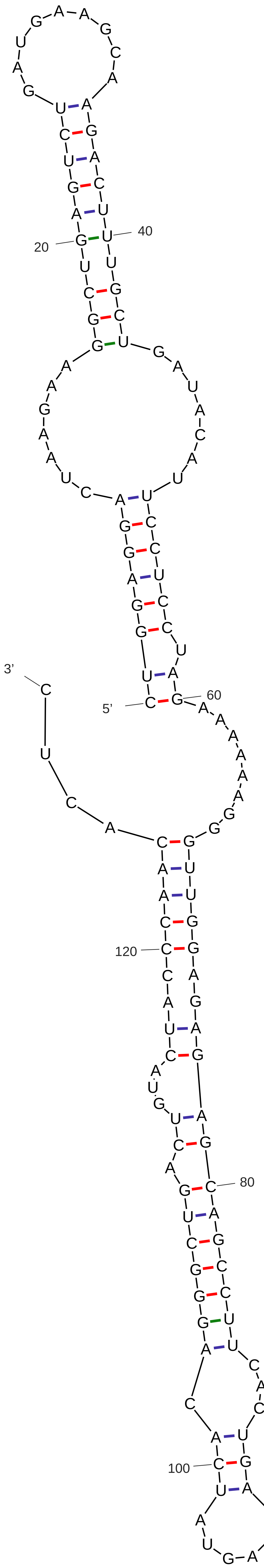

dG = -39.70 [initially -39.70] SCARNA4

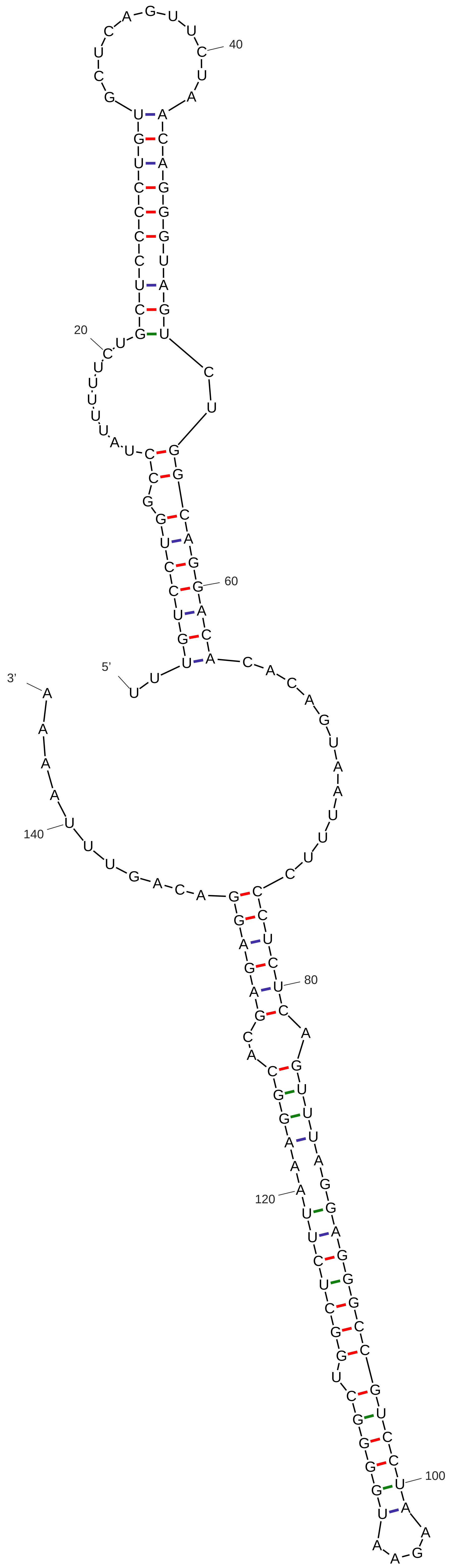

dG = -53.80 [initially -53.80] SCARNA11

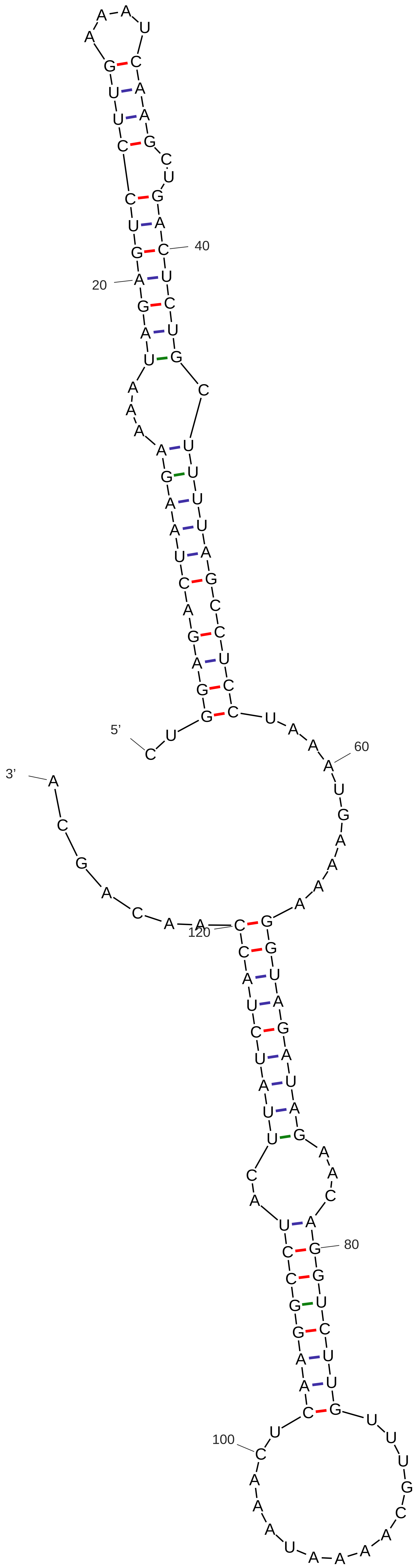

dG = -43.30 [initially -43.30] SCARNA15

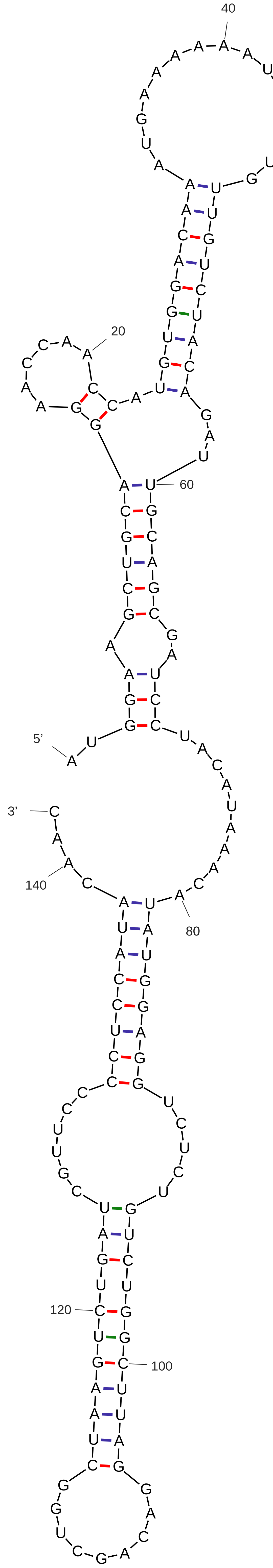

dG = -41.30 [initially -43.60] SCARNA25

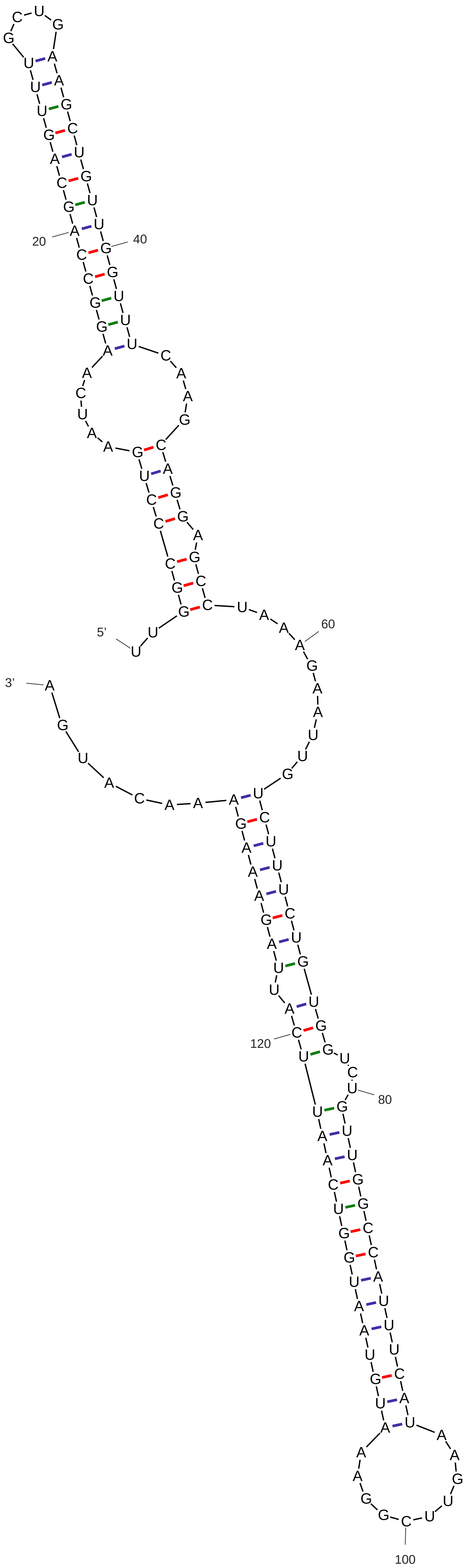

dG = -49.00 [initially -49.00] SNORA2

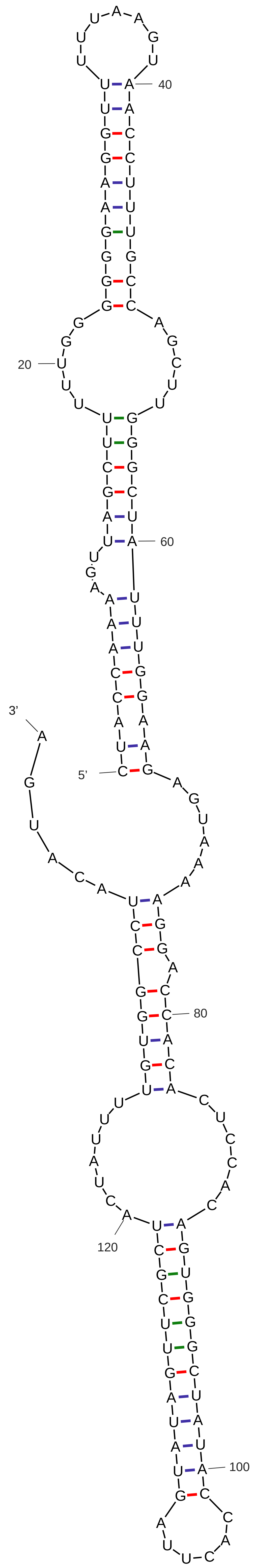

dG = -47.60 [initially -47.60] SNORA4

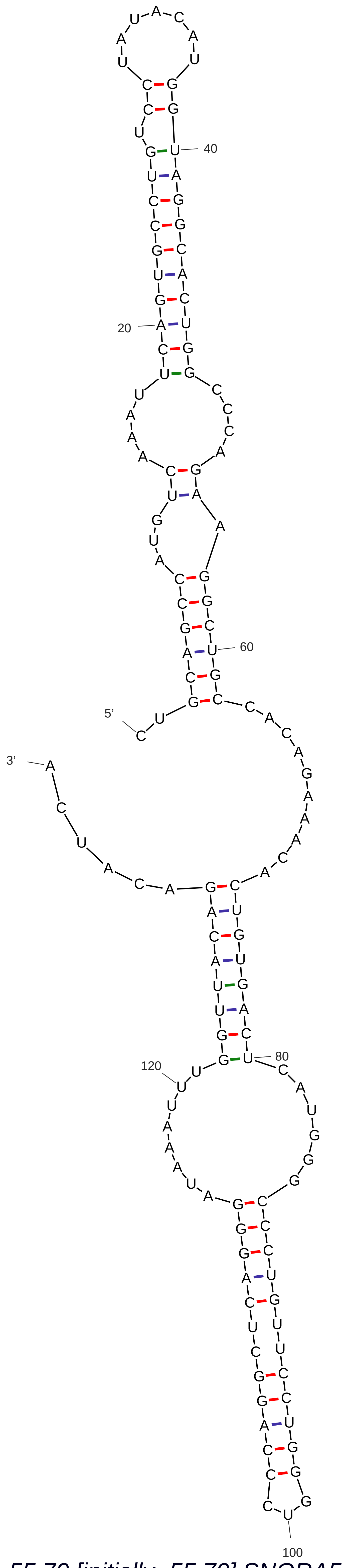

dG = -55.70 [initially -55.70] SNORA5

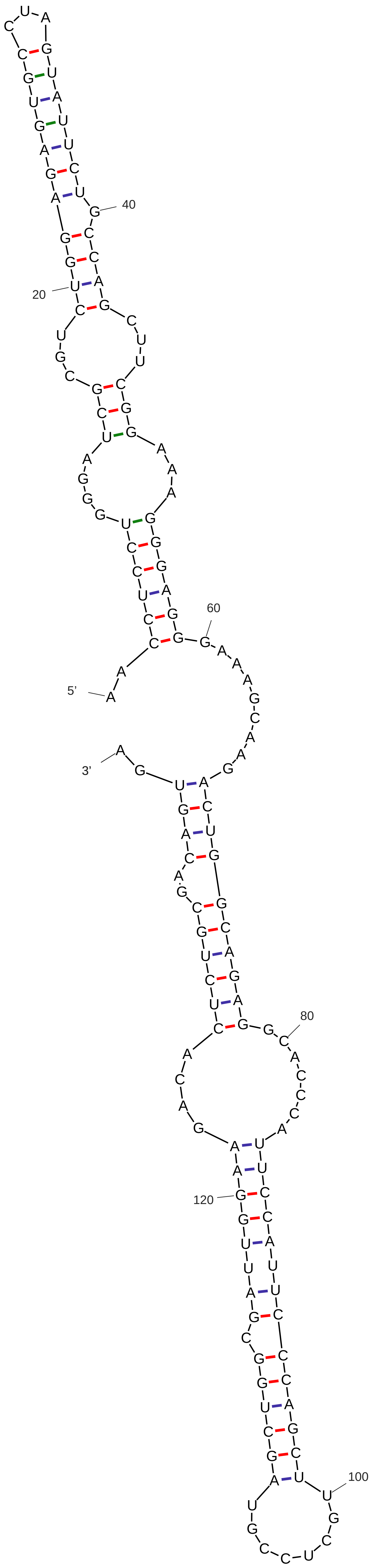

dG = -55.60 [initially -55.60] SNORA7

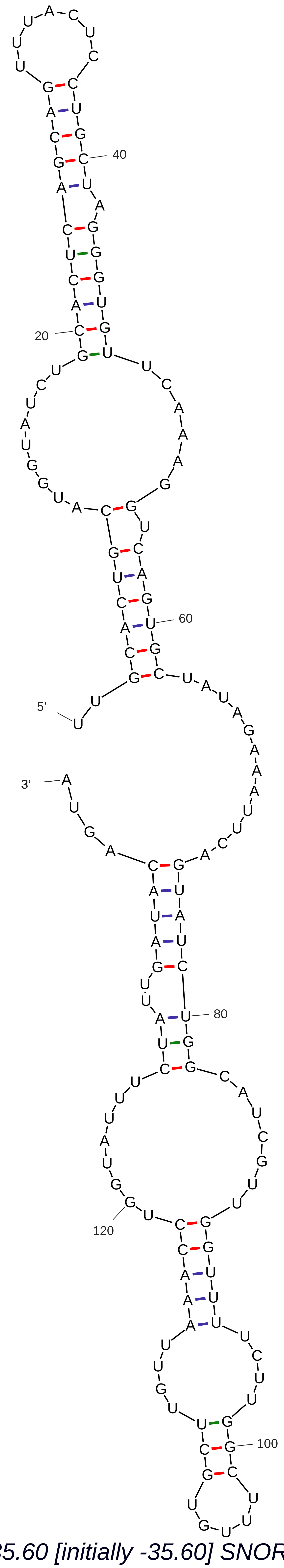

dG = -35.60 [initially -35.60] SNORA8

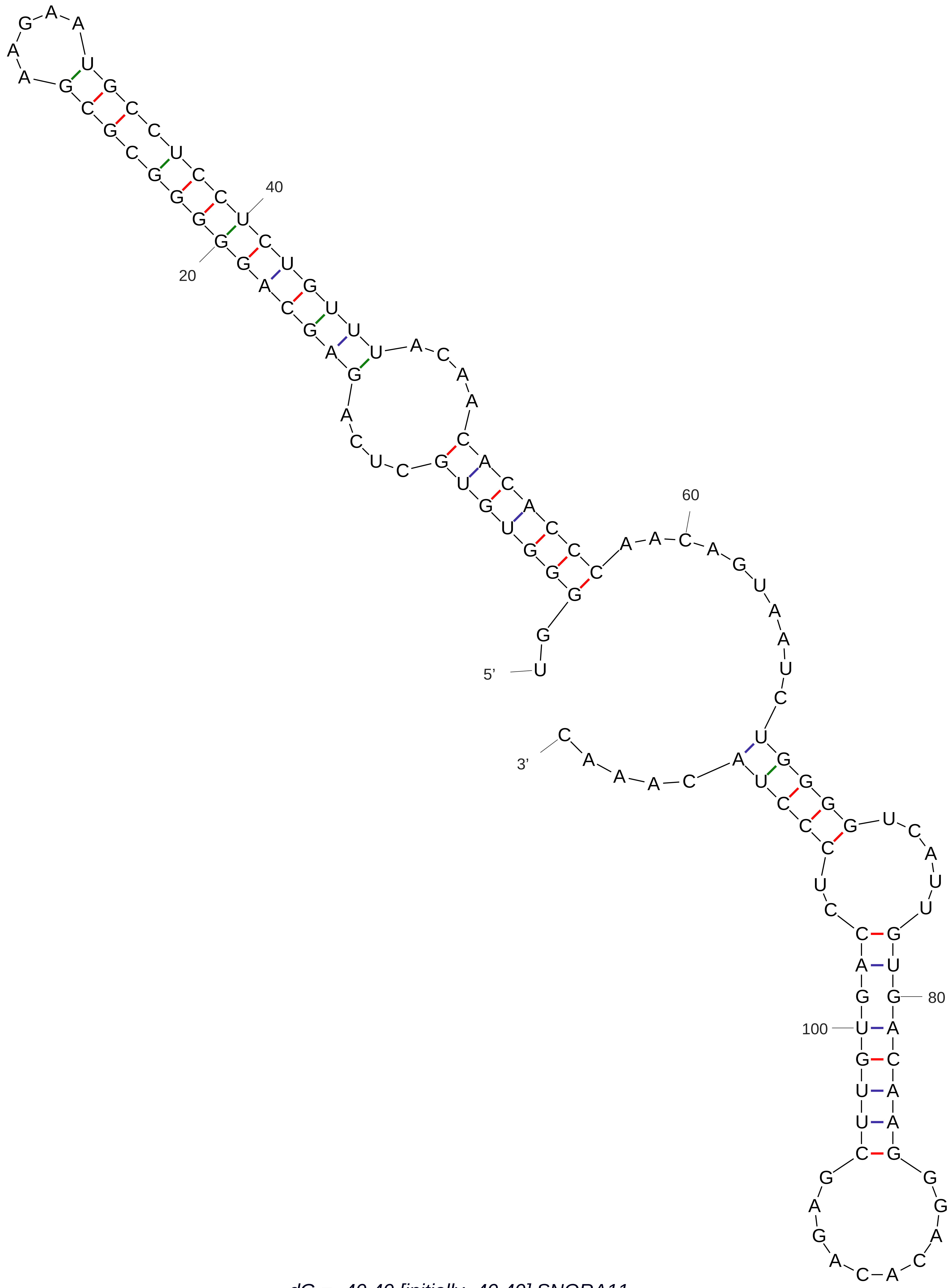

dG = -40.40 [initially -40.40] SNORA11

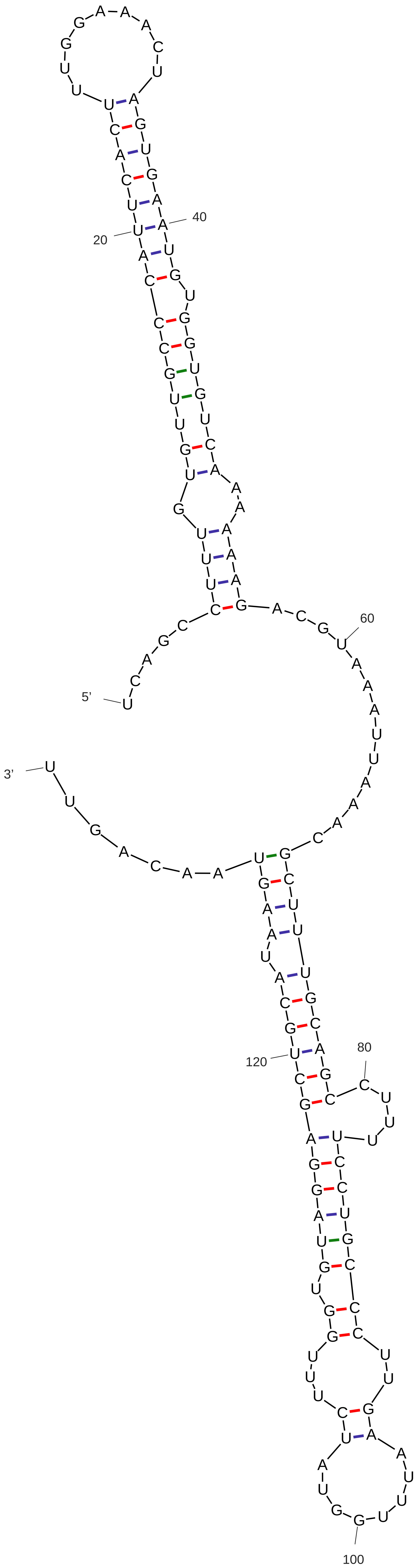

dG = -37.50 [initially -37.80] SNORA13

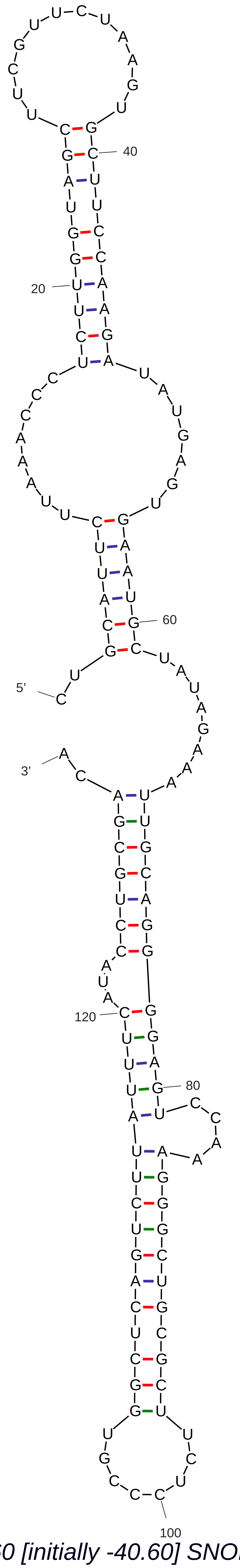

dG = -40.60 [initially -40.60] SNORA14

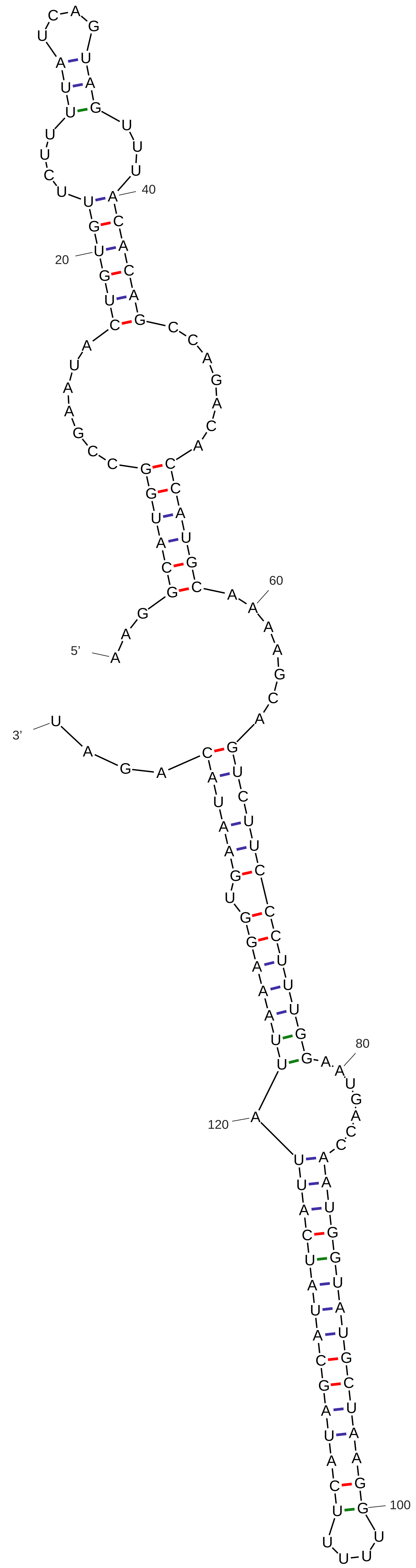

dG = -37.10 [initially -37.10] SNORA15

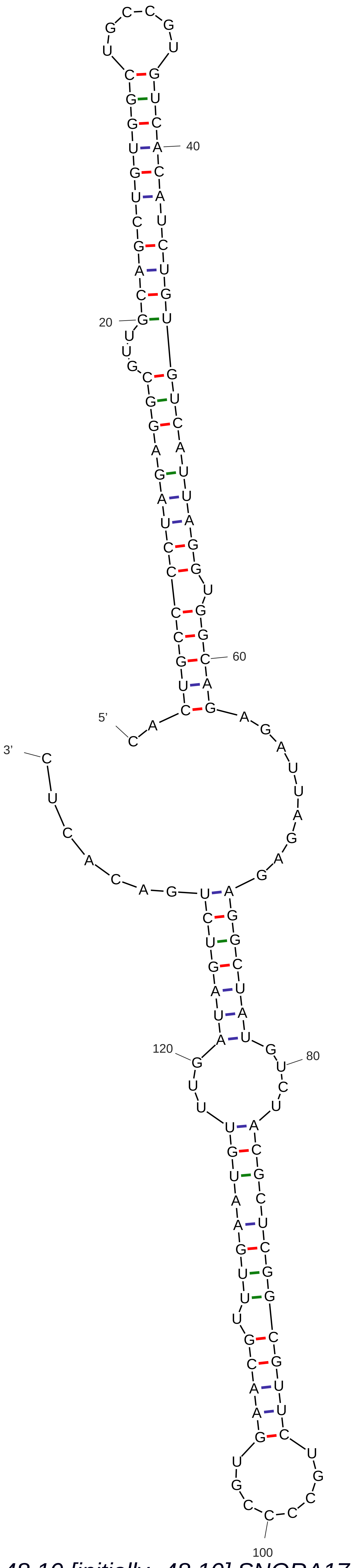

dG = -48.10 [initially -48.10] SNORA17

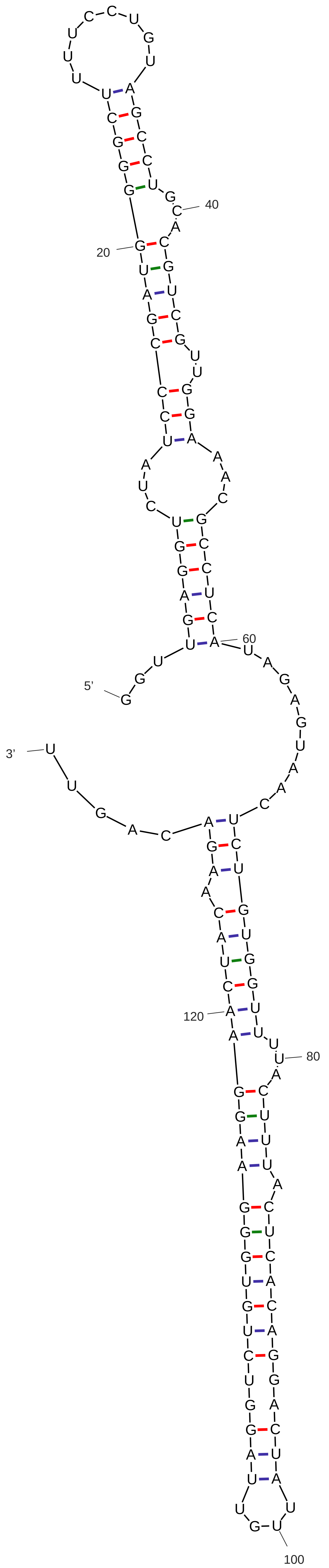

dG = -44.50 [initially -44.50] SNORA18

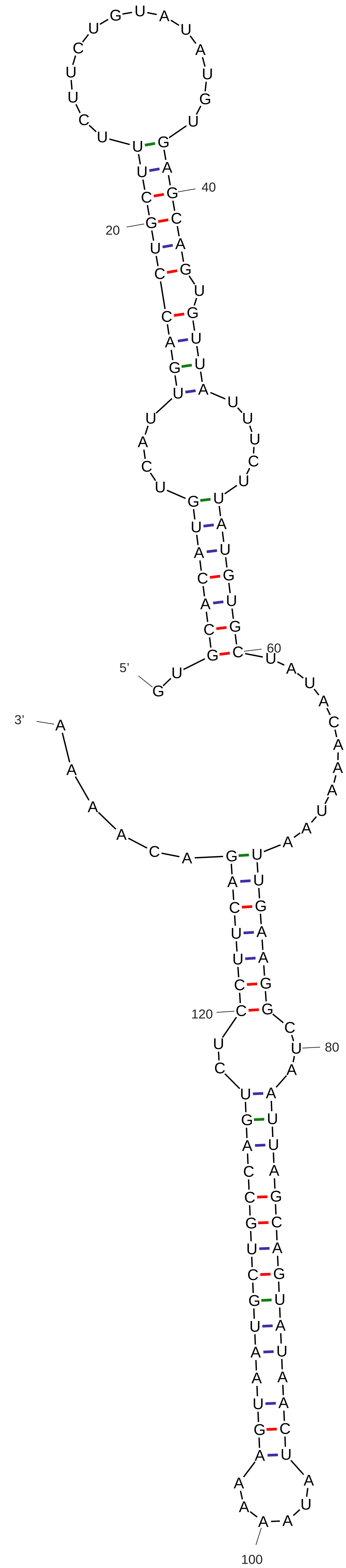

dG = -38.90 [initially -38.90] SNORA19

Output of sir_graph (©)
mfold_util 4.4

Created Mon Nov 23 11:52:18 2009

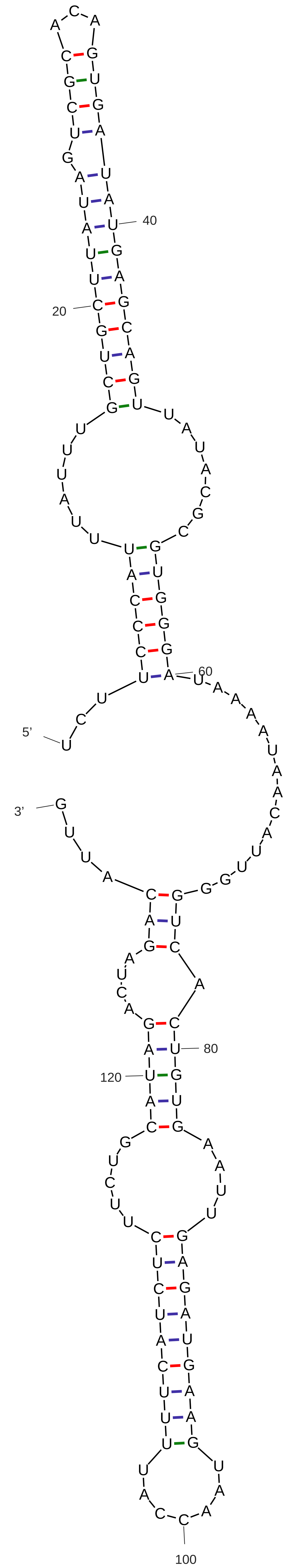

dG = -41.30 [initially -41.30] SNORA20

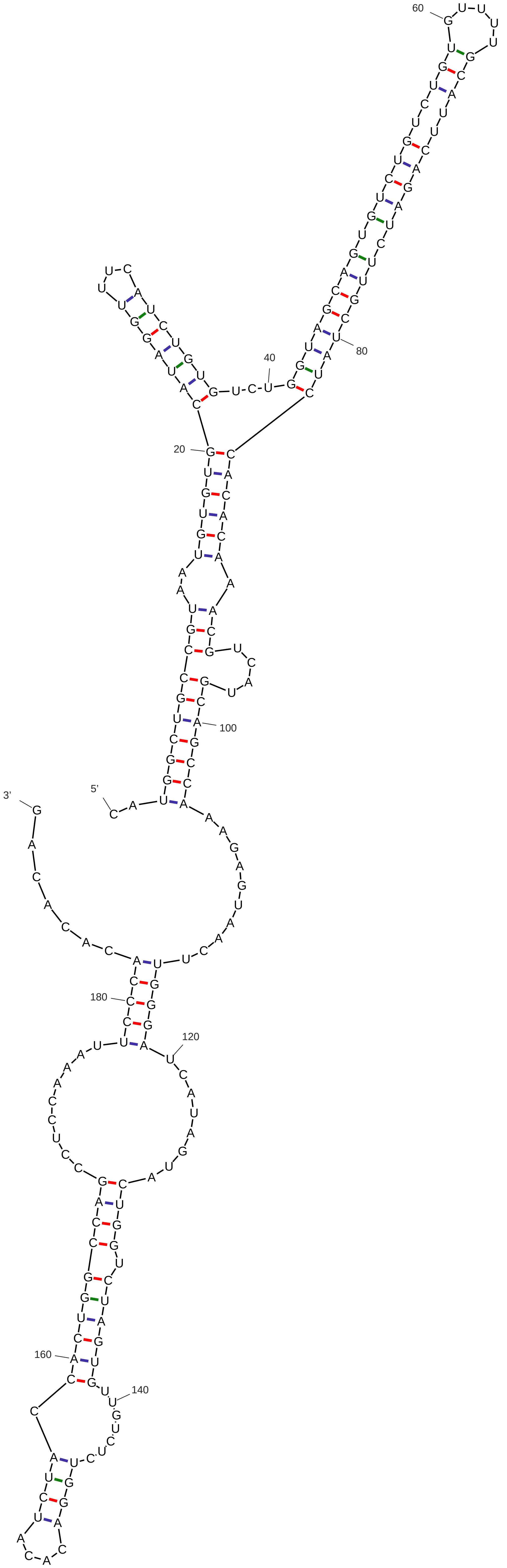

dG = -59.60 [initially -60.00] SNORA23

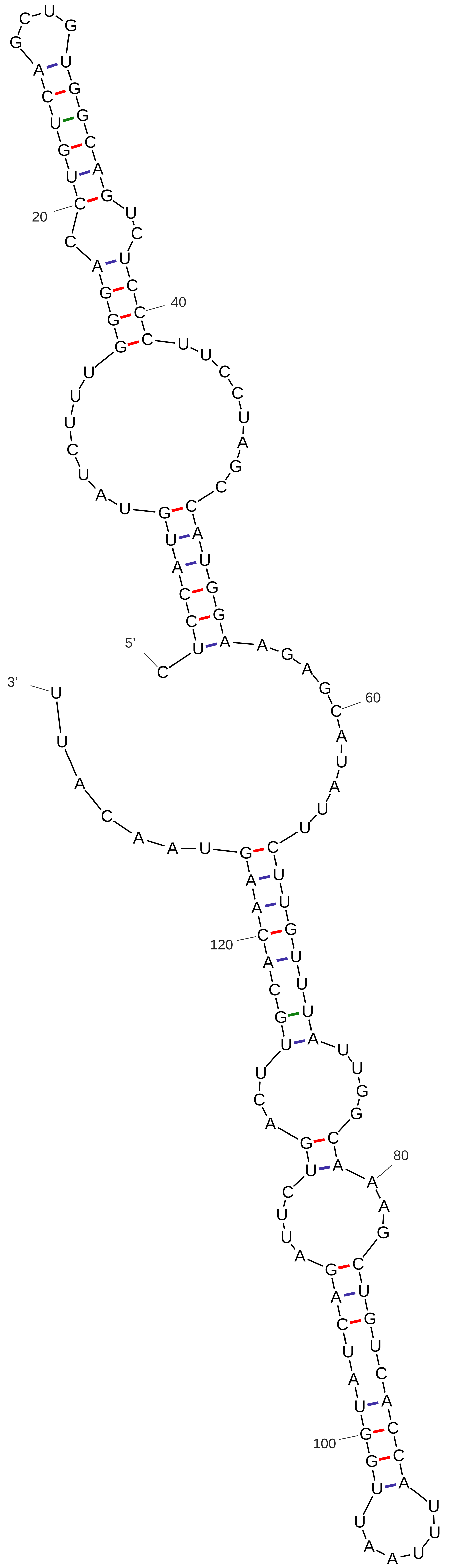

dG = -31.70 [initially -31.70] SNORA24

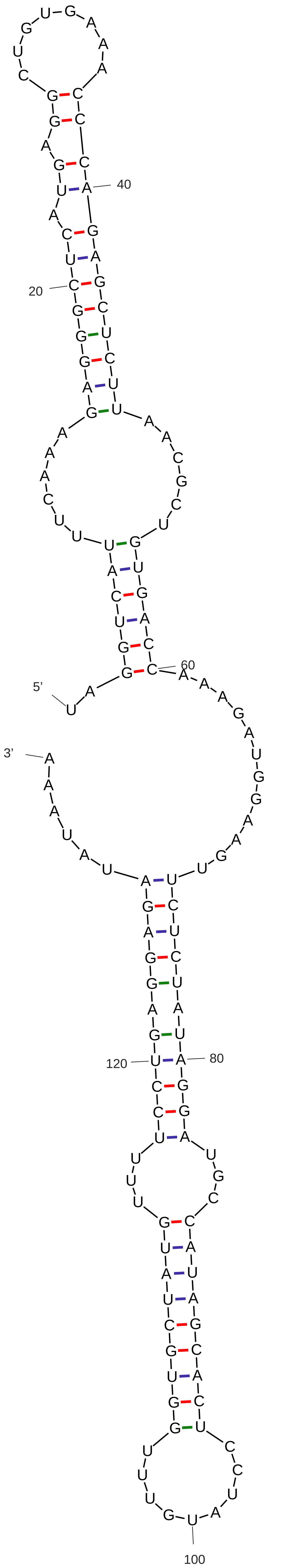

dG = -47.90 [initially -47.90] SNORA25

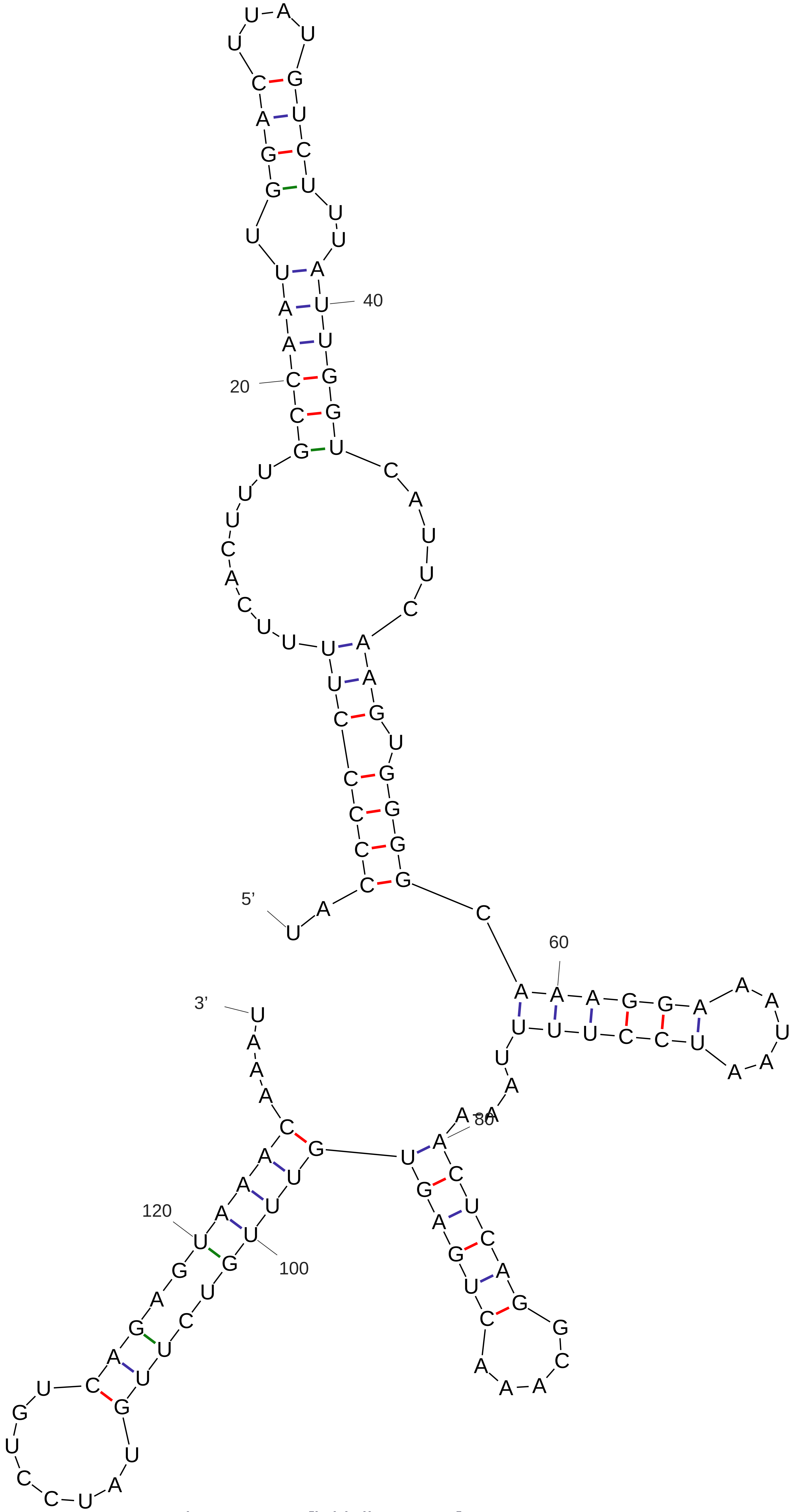

dG = -34.80 [initially -34.40] SNORA27

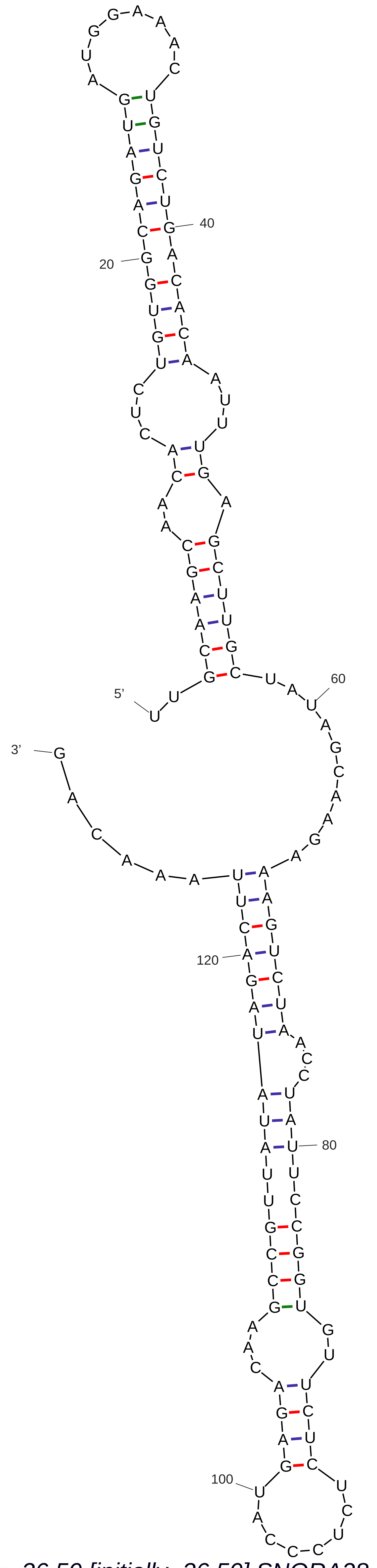

dG = -36.50 [initially -36.50] SNORA28

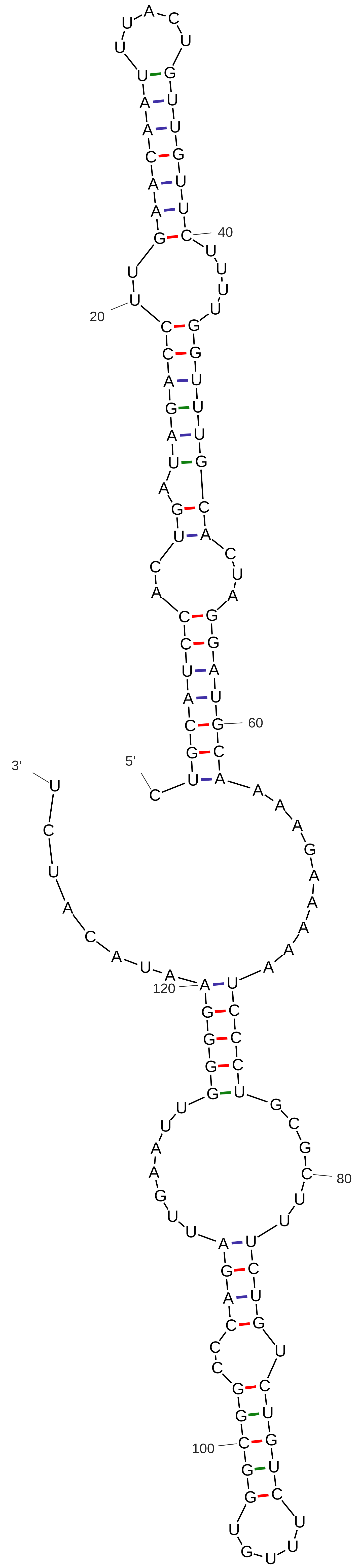

dG = -40.50 [initially -40.50] SNORA31

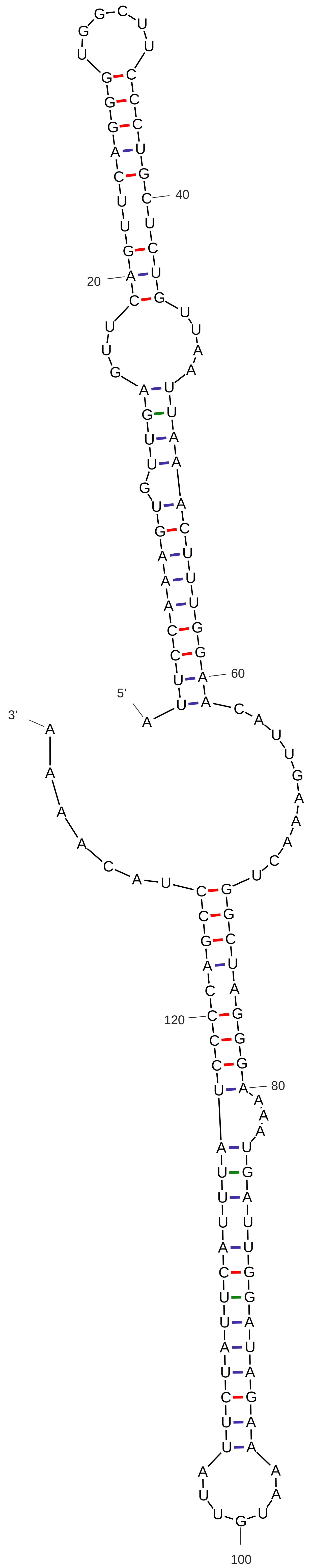

dG = -46.50 [initially -46.50] SNORA36a

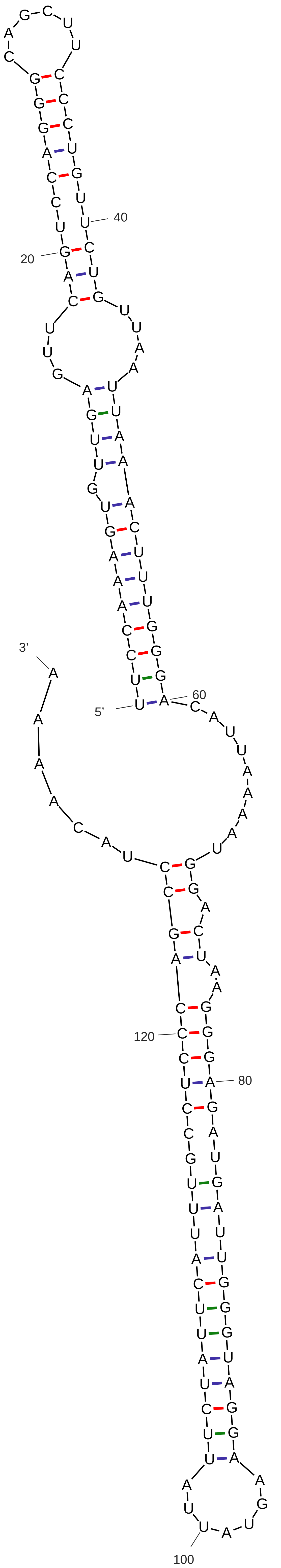

dG = -43.70 [initially -43.70] SNORA36b

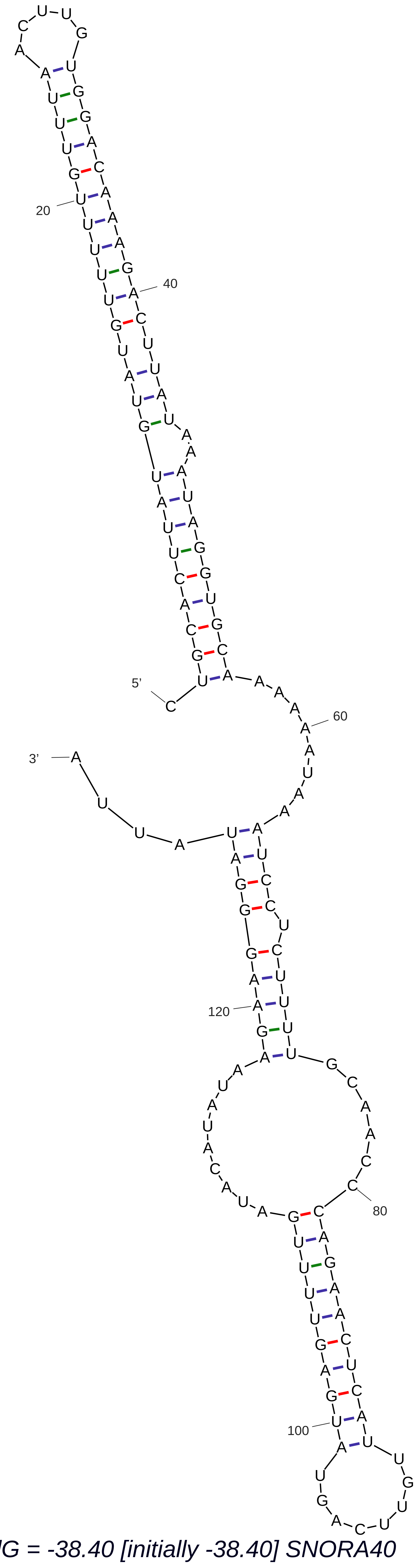

dG = -38.40 [initially -38.40] SNORA40

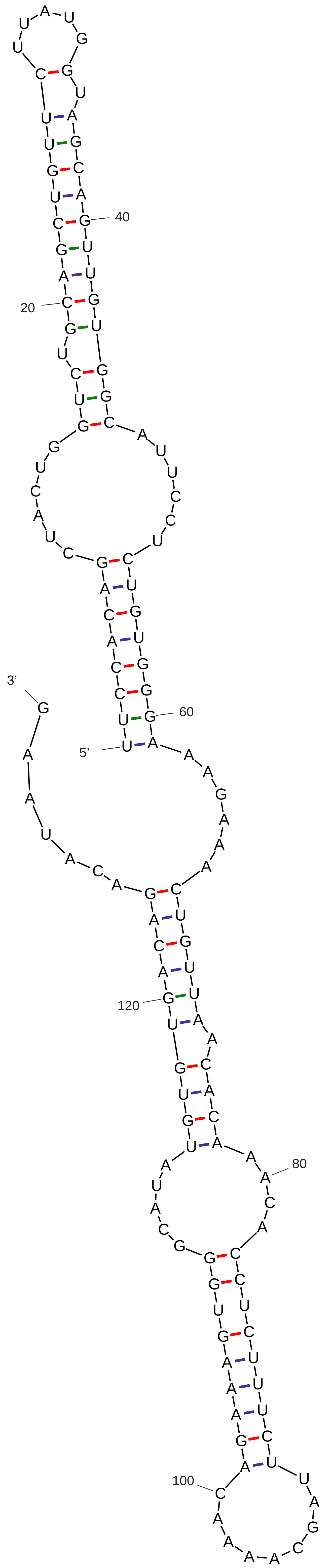

dG = -43.00 [initially -43.00] SNORA41

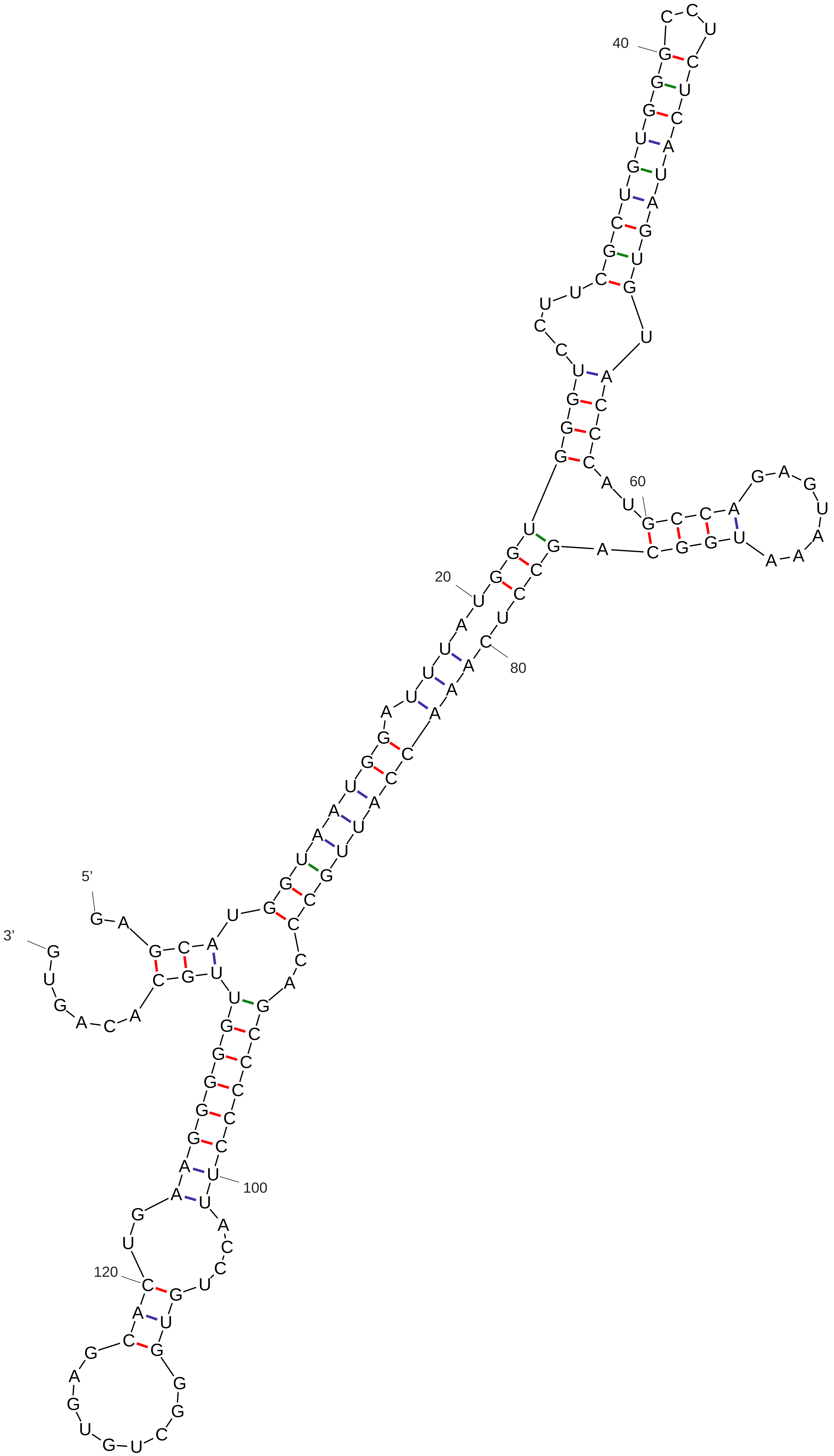

dG = -49.40 [initially -54.10] SNORA42

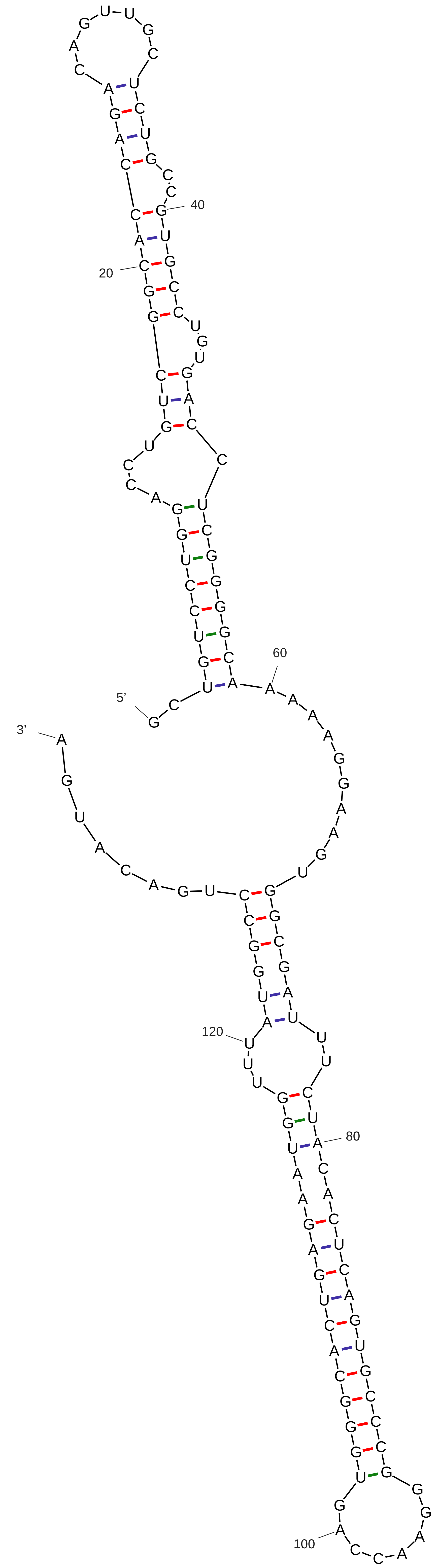

dG = -50.00 [initially -50.00] SNORA43

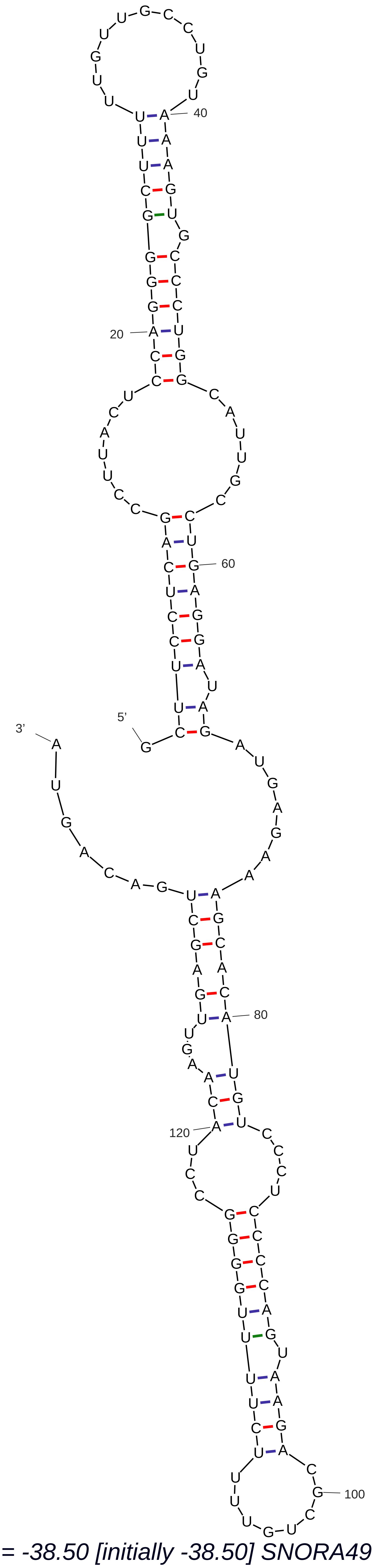

dG = -38.50 [initially -38.50] SNORA49

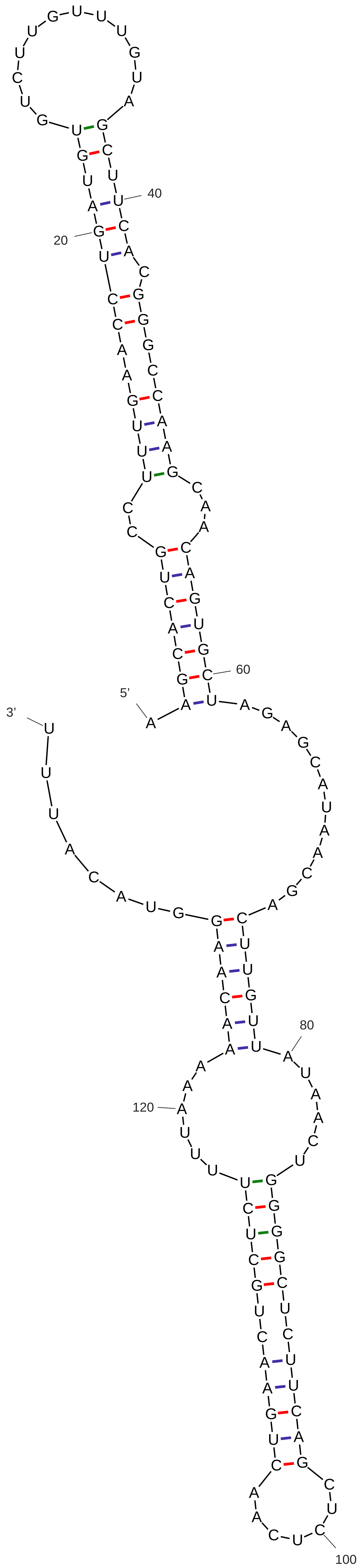

dG = -35.00 [initially -35.00] SNORA50

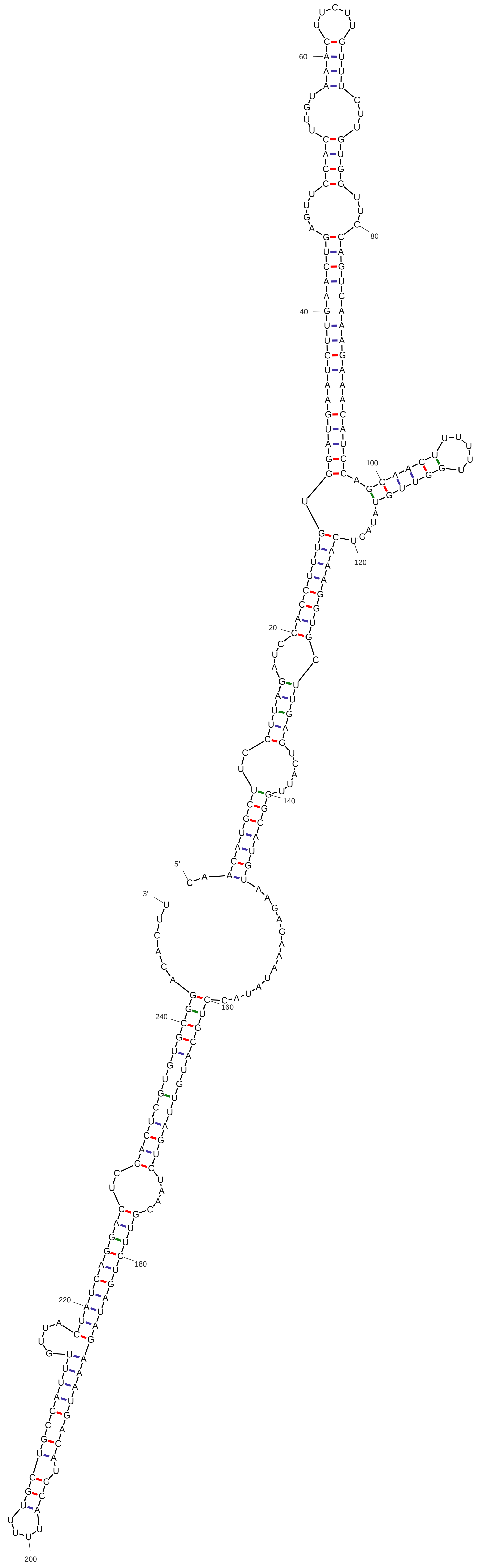

dG = -78.83 [initially -81.10] SNORA53

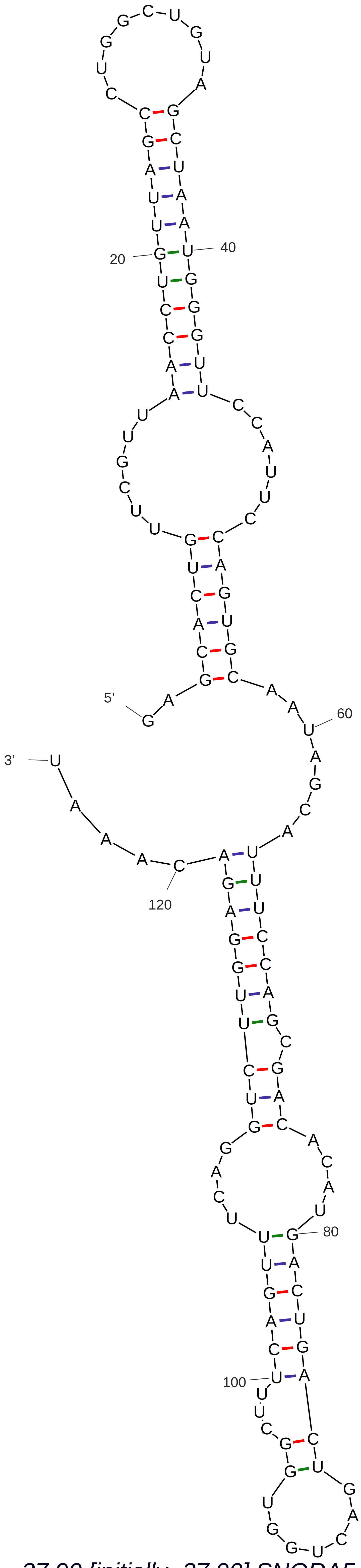

dG = -37.90 [initially -37.90] SNORA54

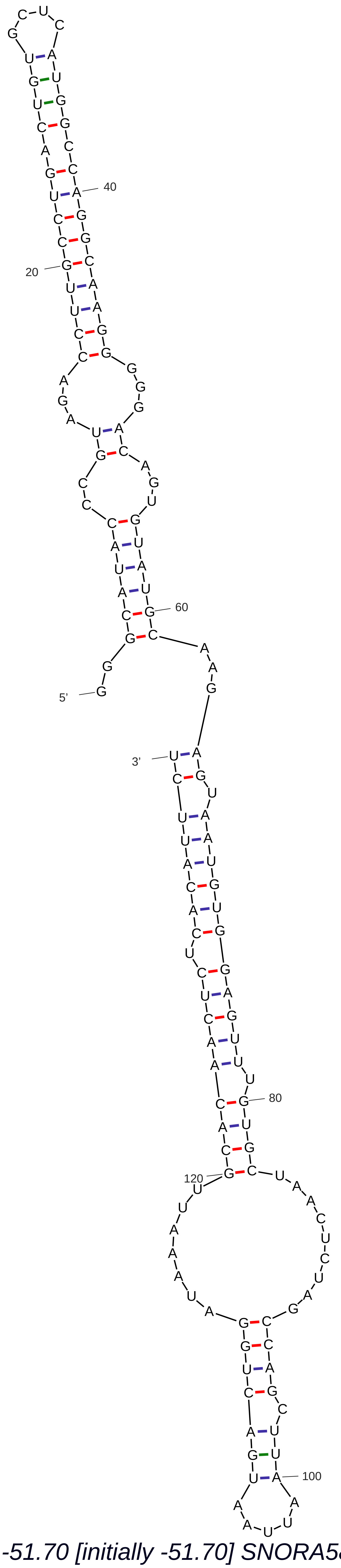

dG = -51.70 [initially -51.70] SNORA58

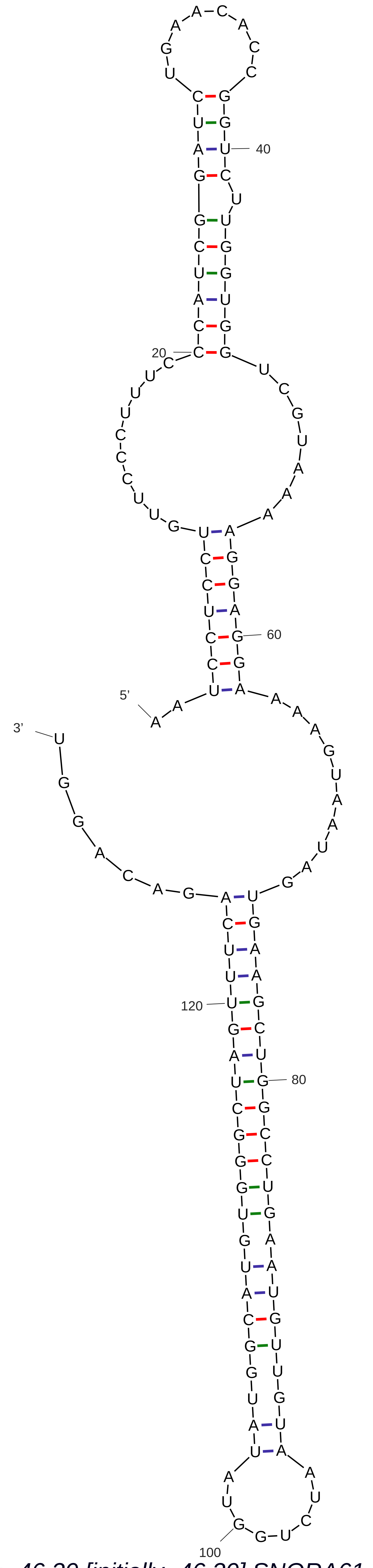

dG = -46.30 [initially -46.30] SNORA61

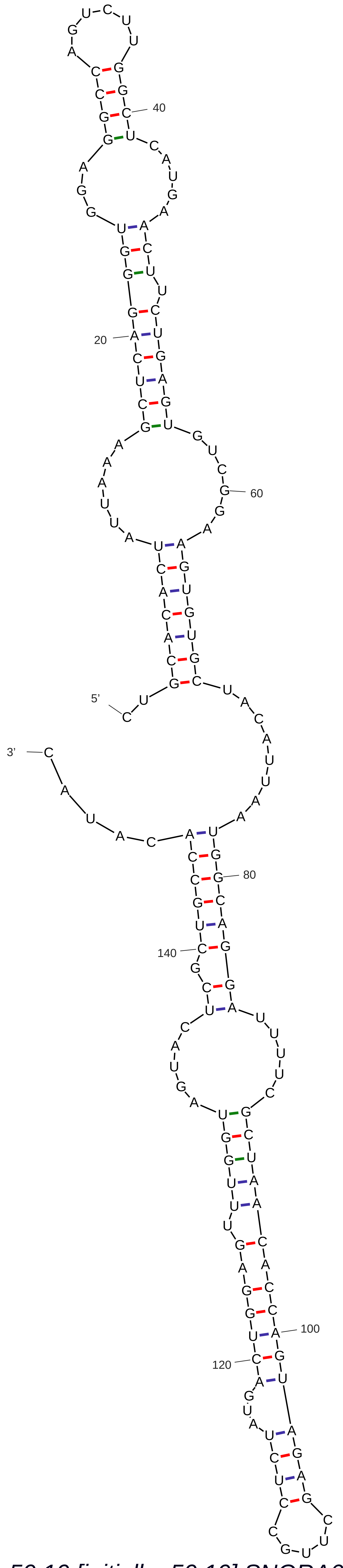

dG = -50.10 [initially -50.10] SNORA62

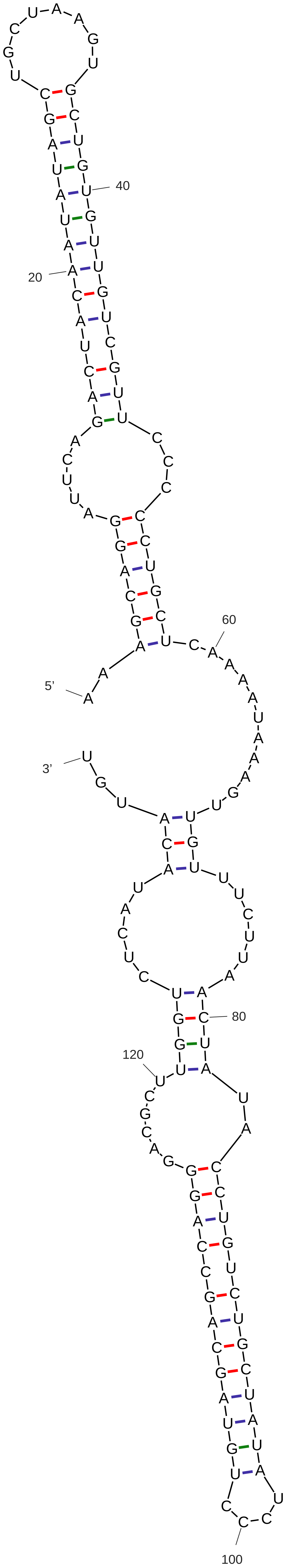

dG = -41.30 [initially -41.30] SNORA63

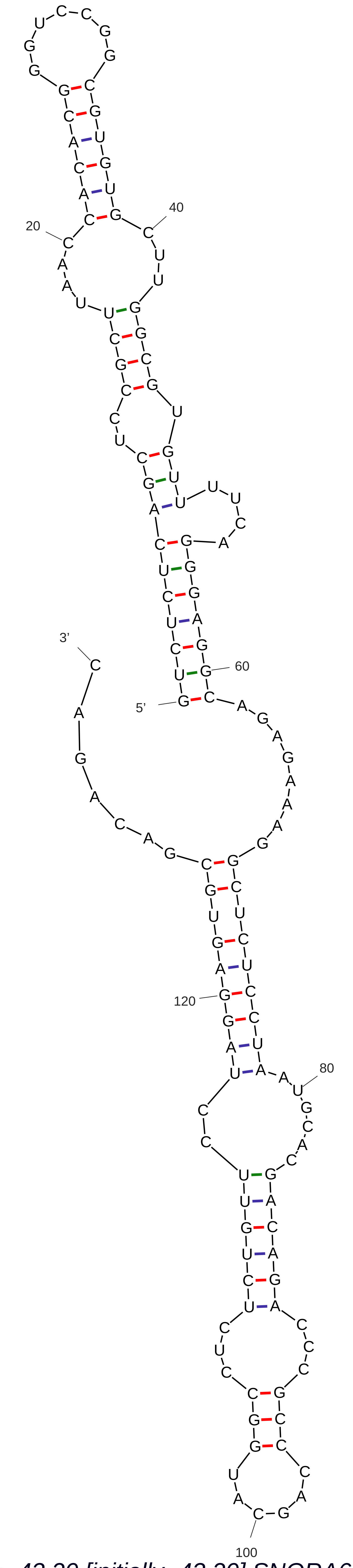

dG = -43.20 [initially -43.20] SNORA64

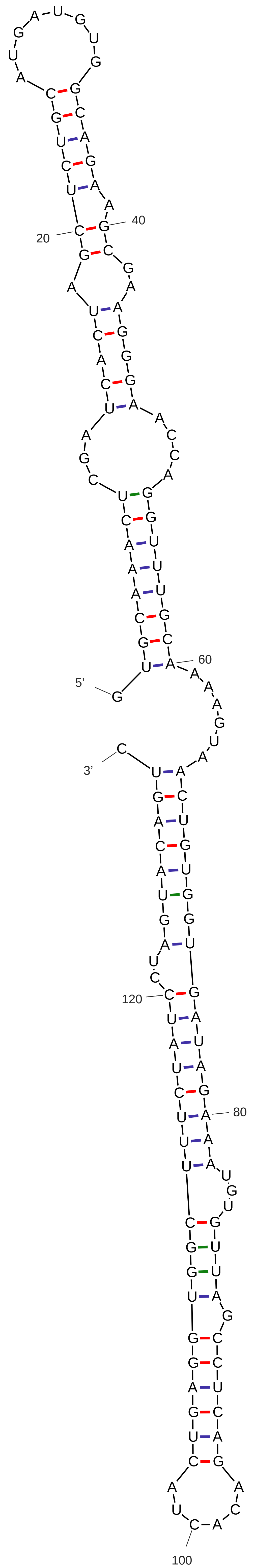

dG = -44.00 [initially -44.00] SNORA66

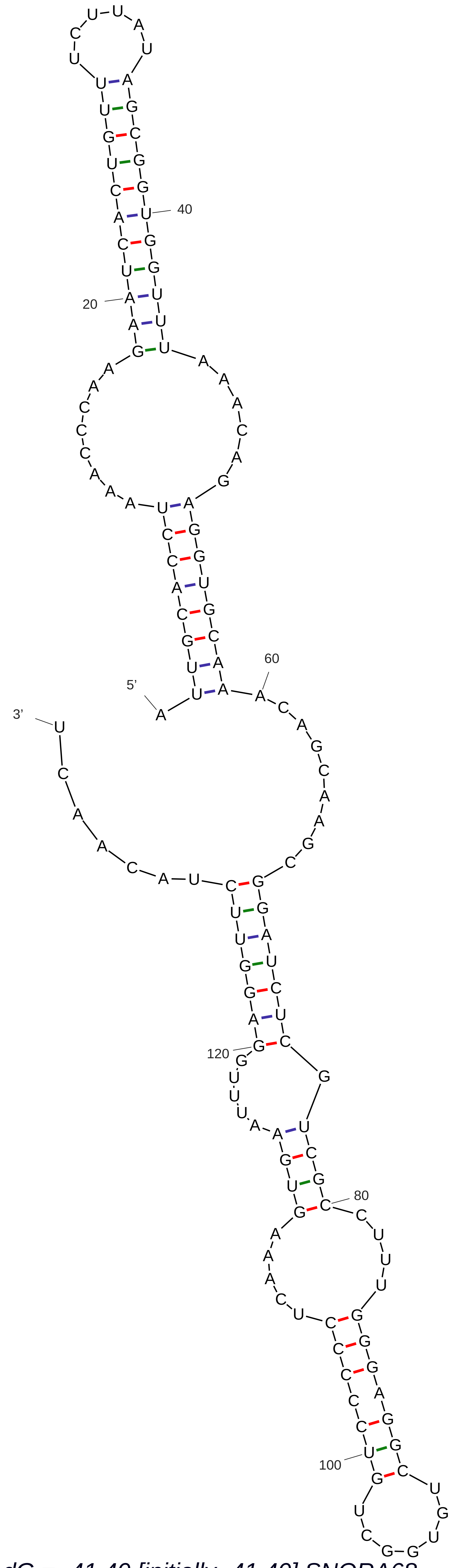

dG = -41.40 [initially -41.40] SNORA68

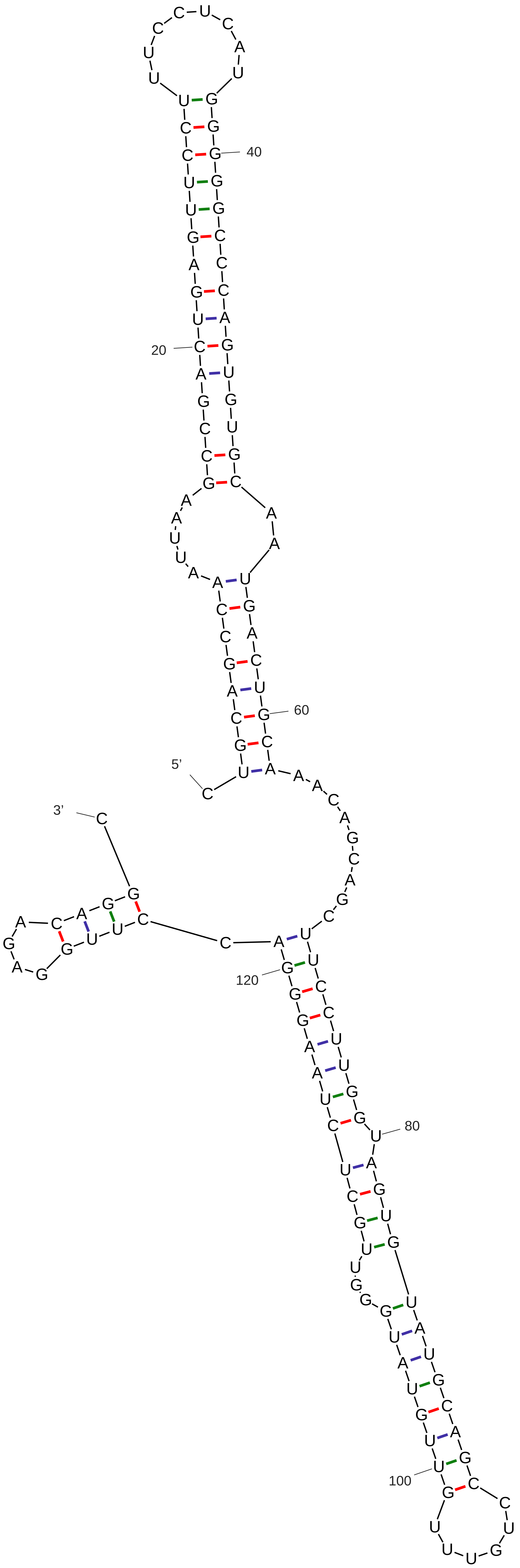

dG = -44.70 [initially -42.20] SNORA70

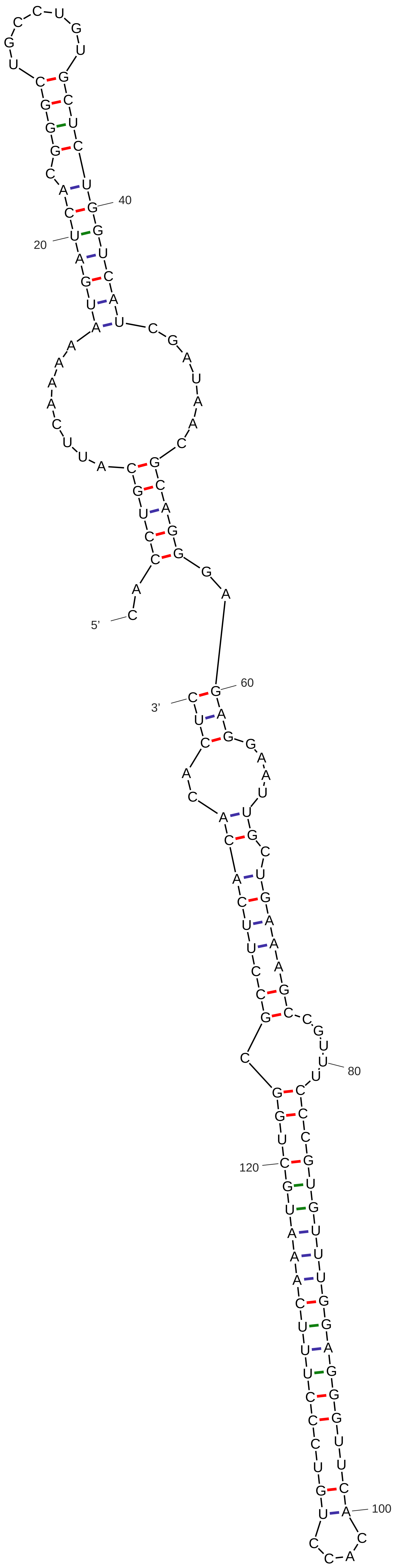

dG = -43.40 [initially -43.40] SNORA71

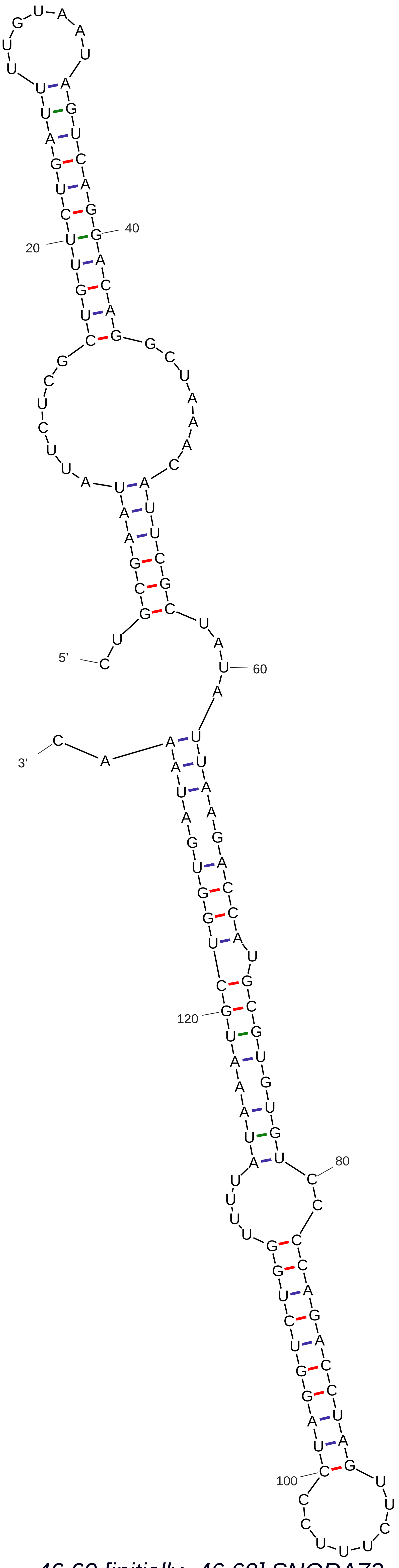

dG = -46.60 [initially -46.60] SNORA72

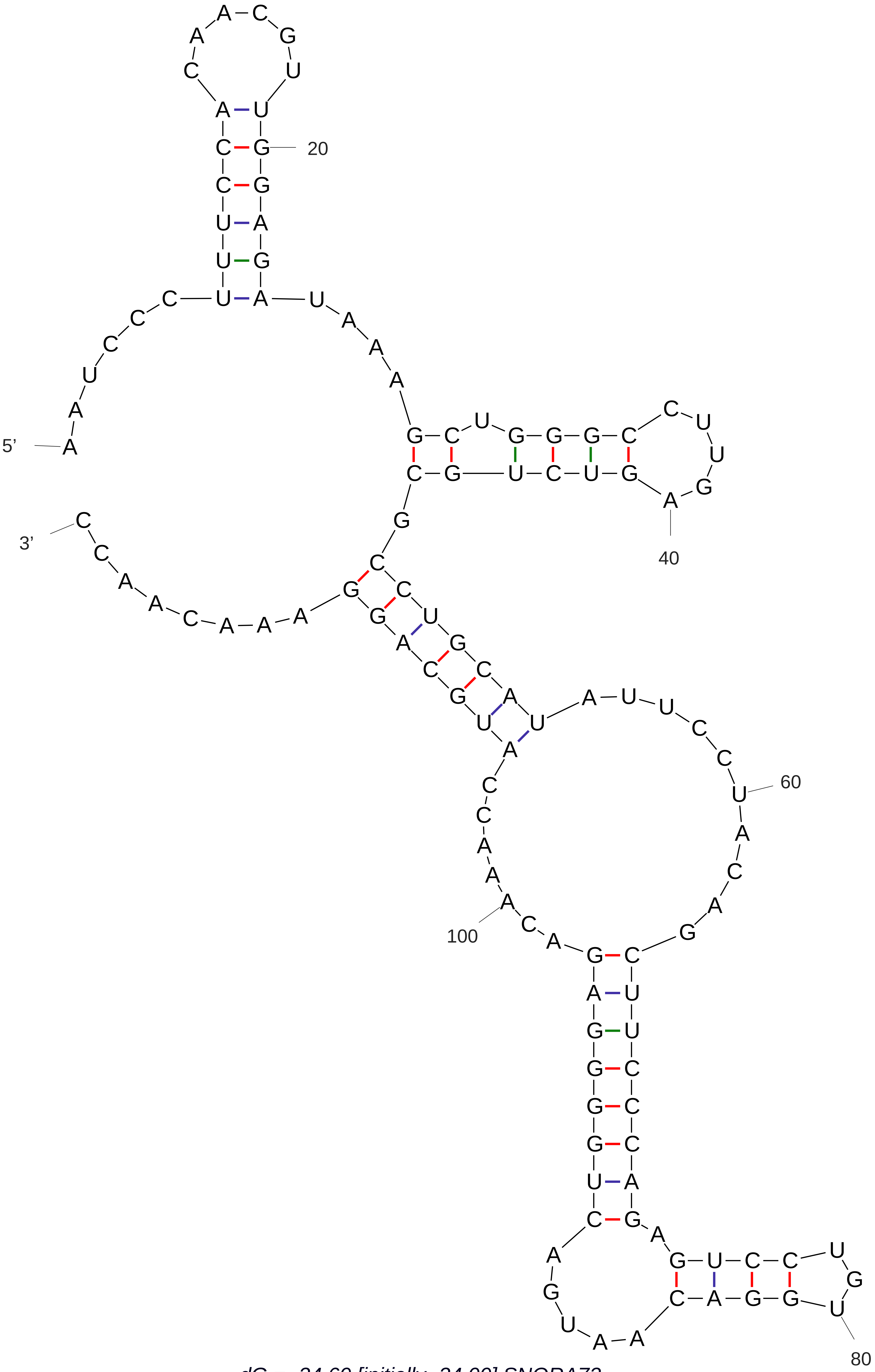

dG = -34.60 [initially -34.00] SNORA73

Output of sir_graph (©)
mfold_util 4.4

Created Mon Nov 23 14:05:03 2009

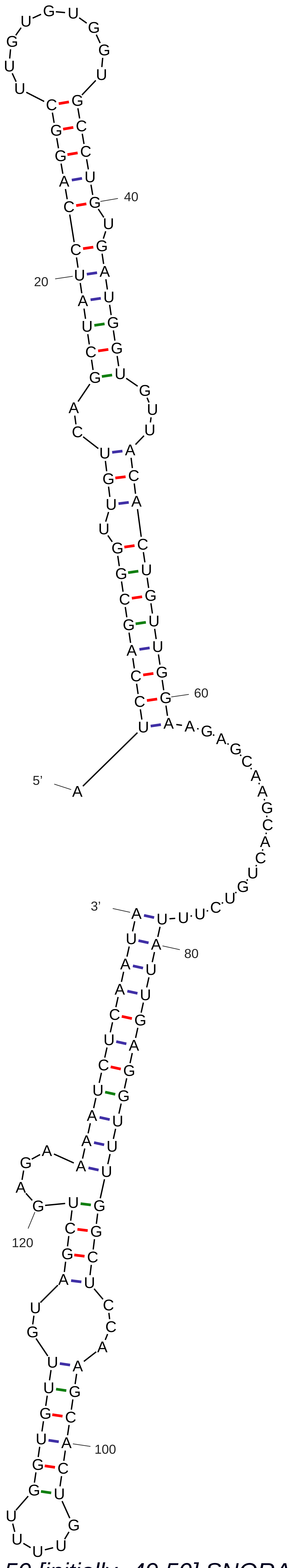

dG = -49.50 [initially -49.50] SNORA74a

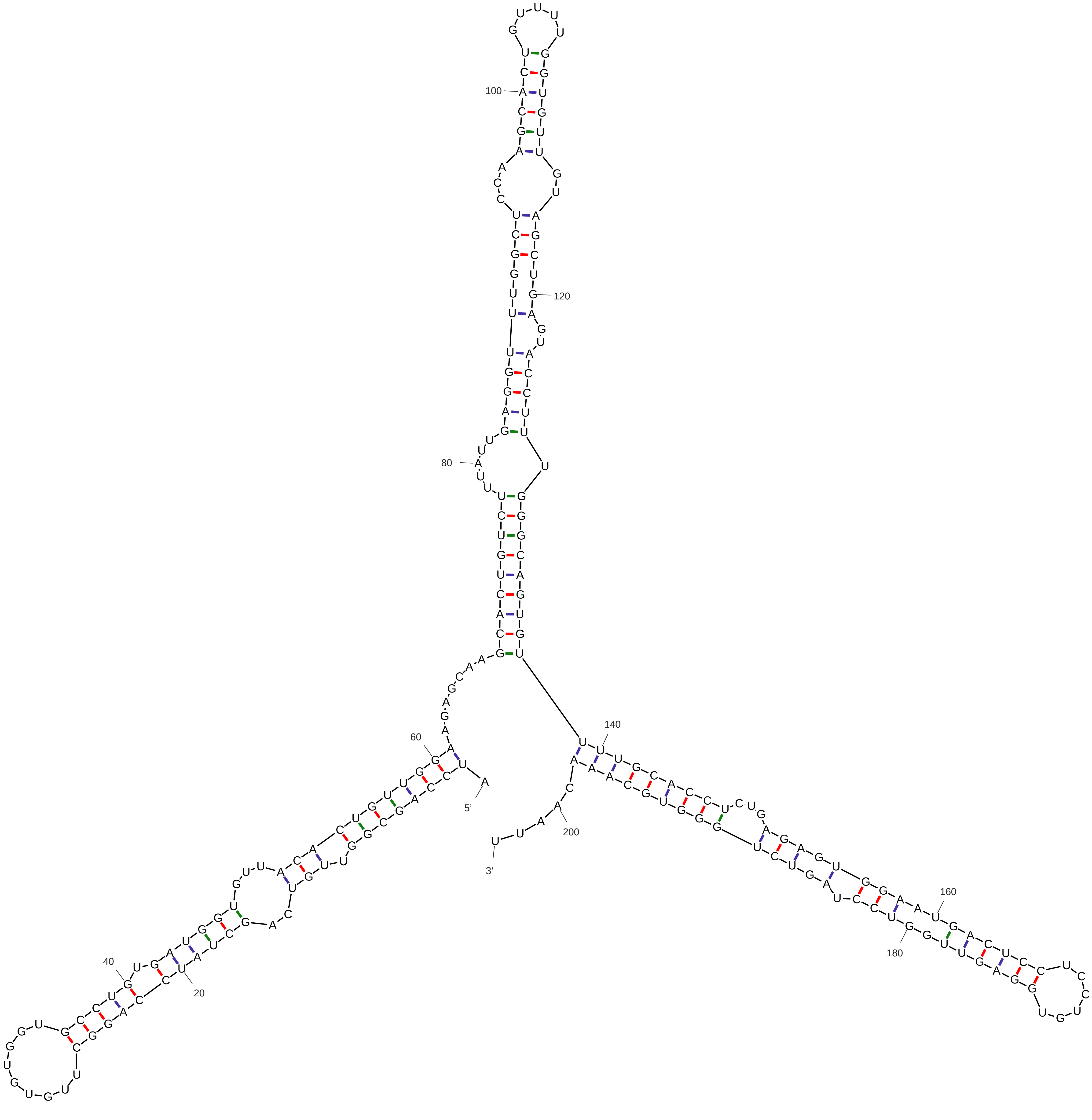

dG = -85.80 [initially -85.60] SNORA74b

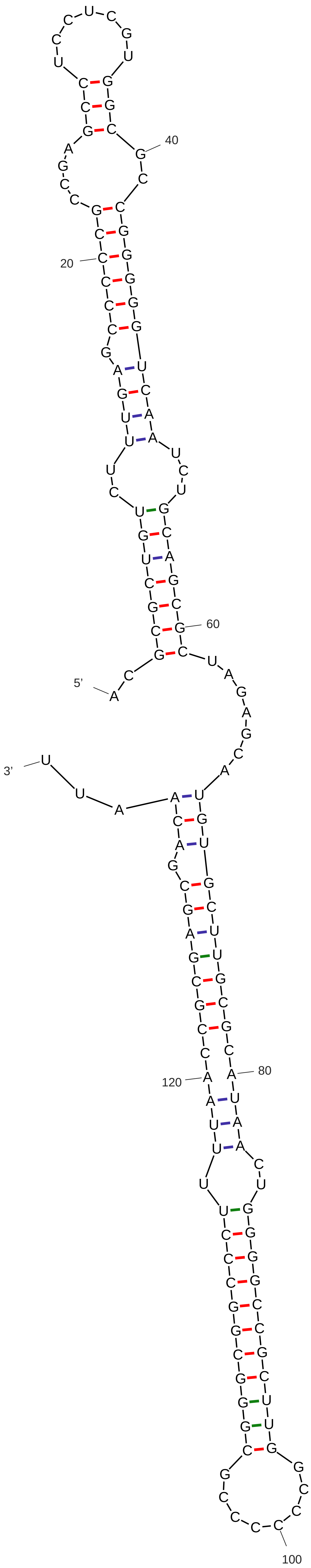

dG = -69.80 [initially -69.80] SNORA76

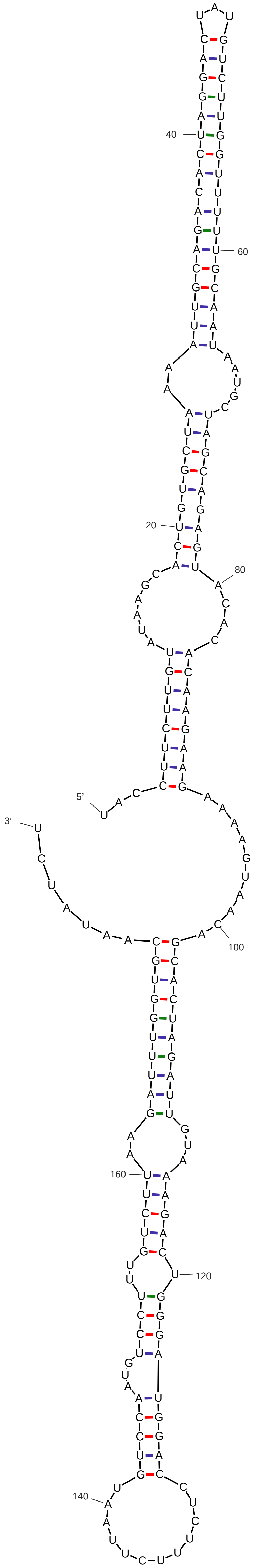

dG = -62.10 [initially -62.10] SNORA81
