# Supplementary material for: Systematic identification and evolutionary features of rhesus monkey small nucleolar RNAs
Source: BMC Genomics. 2010 Jan 25;11:61. doi: 10.1186/1471-2164-11-61 (PMC2832892; doi:10.1186/1471-2164-11-61)
Supplement: Additional file 3 — Target prediction of C/D box snoRNAs. The alignments of guide sequences with target sequences are shown. [file 1471-2164-11-61-S3.pdf]

Additional file 3

Target site prediction of 32 C/D box snoRNAs of rhesus monkey

36 target sites from rRNAs.
16 target sites from snRNAs.

SNORD116 Prime5 Guide Sequence

> ENSMMUG00000024631|ENSMMUT00000034332|U1 spliceosomal RNA [Source:RFAM;Acc:RF00003]

```
Query  19  CCTTGGAAAA  28
           ||||||||||
Sbjct  48  CCTTGGAAAA  39
```

SNORD116 Prime3 Guide Sequence

> ENSMMUG00000034328|ENSMMUT00000050062|Small subunit ribosomal RNA, 5` domain [Source:RFAM;Acc:RF00177]

```
Query  15  GTTCTCAT  22
           ||||||||
Sbjct  22  GTTCTCAT  15
```

SNORD13 Prime5 Guide Sequence

> ENSMMUG00000027465|ENSMMUT00000037148|U6 spliceosomal RNA [Source:RFAM;Acc:RF00026]

```
Query  8   TTCACACGTTTGTGTG  23
           |||||| |||||||||
Sbjct  92  TTCACAAGTTTGTGTG  77
```

SNORD15 Prime5 Guide Sequence

> ENSMMUG00000033040|ENSMMUT00000048774|5S ribosomal RNA [Source:RFAM;Acc:RF00001]

```
Query  11  AGGTCACCTC  20
           ||||||||||
Sbjct  84  AGGTCACCTC  75
```

SNORD15 Prime3 Guide Sequence

> ENSMMUG00000034740|ENSMMUT00000050474|Small subunit ribosomal RNA, 5` domain [Source:RFAM;Acc:RF00177]

```
Query  3    TAGA-GGCATTT  13
            |||| |||||||
Sbjct  280  TAGAGGGCATTT  269

Query  8    GCATTTGT  15
            ||||||||
Sbjct  325  GCATTTGT  318
```

SNORD16 Prime5 Guide Sequence

> ENSMMUG00000025208|ENSMMUT00000034909|U6 spliceosomal RNA [Source:RFAM;Acc:RF00026]

```
Query  5   AATTTGCGTCTTA  17
           |||||||||||||
Sbjct  86  AATTTGCGTCTTA  74
```

SNORD16 Prime3 Guide Sequence

> ENSMMUG00000035358|ENSMMUT00000051092|U6 spliceosomal RNA [Source:RFAM;Acc:RF00026]

```
Query  1   AAATTTTTA  9
           |||||||||
Sbjct  93  AAATTTTTA  85
```

SNORD17a Prime5 Guide Sequence

> ENSMMUG00000034913|ENSMMUT00000050647|Small subunit ribosomal RNA, 5` domain [Source:RFAM;Acc:RF00177]

```
Query  1    TTCAGTTTAT  10
            ||||||||||
Sbjct  607  TTCAGTTTAT  598

Query  3    CAGTTTAT  10
            ||||||||
Sbjct  752  CAGTTTAT  745
```

SNORD17a Prime3 Guide Sequence

> ENSMMUG00000025555|ENSMMUT00000035256|U1 spliceosomal RNA [Source:RFAM;Acc:RF00003]

```
Query  4   TCTGATCACT  13
           ||||||||||
Sbjct  25  TCTGATCACT  16
```

SNORD17b Prime5 Guide Sequence

> ENSMMUG00000034913|ENSMMUT00000050647|Small subunit ribosomal RNA, 5` domain [Source:RFAM;Acc:RF00177]

```
Query  1    TTCAGTTTAT  10
            ||||||||||
Sbjct  607  TTCAGTTTAT  598

Query  3    CAGTTTAT  10
            ||||||||
Sbjct  752  CAGTTTAT  745
```

SNORD17b Prime3 Guide Sequence

> ENSMMUG00000034903|ENSMMUT00000050637|U2 spliceosomal RNA [Source:RFAM;Acc:RF00004]

```
Query  1   GTGGTGAAAAT  11
           |||| ||||||
Sbjct  80  GTGGAGAAAAT  70
```

SNORD22 Prime5 Guide Sequence

> ENSMMUG00000033137|ENSMMUT00000048871|U6 spliceosomal RNA [Source:RFAM;Acc:RF00026]

```
Query  3   CTTTCACACG  12
           ||||||||||
Sbjct  66  CTTTCACACG  57
```

SNORD24 Prime5 Guide Sequence

>ENSMMUG00000024740|ENSMMUT00000034441|U6 spliceosomal RNA [Source:RFAM;Acc:RF00026]

```
Query  2   AGTTTATTTGCTACT  16
           ||| |||||||||||
Sbjct  22  AGTATATTTGCTACT  8
```

SNORD24 Prime3 Guide Sequence

> ENSMMUG00000034810|ENSMMUT00000050544|Small subunit ribosomal RNA, 5` domain [Source:RFAM;Acc:RF00177]

```
Query  14   CATTATCTT  22
            |||||||||
Sbjct  370  CATTATCTT  362
```

SNORD26 Prime5 Guide Sequence

> ENSMMUG00000025929|ENSMMUT00000035630|U6 spliceosomal RNA [Source:RFAM;Acc:RF00026]

```
Query  1   TTTTACAAA  9
           |||||||||
Sbjct  91  TTTTACAAA  83
```

SNORD26 Prime3 Guide Sequence

> ENSMMUG00000034903|ENSMMUT00000050637|U2 spliceosomal RNA [Source:RFAM;Acc:RF00004]

```
Query  3  AGTGGAGAAAACAAAAATT  21
           ||||||||||| ||| |||
Sbjct  81  AGTGGAGAAAATAAACATT  63
```

SNORD27 Prime5 Guide Sequence

> ENSMMUG00000028048|ENSMMUT00000037685|5S ribosomal RNA [Source:RFAM;Acc:RF00001]

```
Query  11  ACAAGCATAT  20
           ||||||||||
Sbjct  32  ACAAGCATAT  23
```

SNORD27 Prime3 Guide Sequence

> ENSMMUG00000035124|ENSMMUT00000050858|Small subunit ribosomal RNA, 5` domain [Source:RFAM;Acc:RF00177]

```
Query  3    ATCTTACTA  11
            |||||||||
Sbjct  255  ATCTTACTA  247
```

SNORD45 Prime5 Guide Sequence

> ENSMMUG00000034810|ENSMMUT00000050544|Small subunit ribosomal RNA, 5` domain [Source:RFAM;Acc:RF00177]

```
Query  2   CTGGCATGT  10
           |||||||||
Sbjct  31  CTGGCATGT  23
```

SNORD45 Prime3 Guide Sequence

> ENSMMUG00000027843|ENSMMUT00000037502|Small subunit ribosomal RNA, 5` domain [Source:RFAM;Acc:RF00177]

```
Query  9    TTAGCTCTAGAATTAC  24
            ||||||||||||||||
Sbjct  151  TTAGCTCTAGAATTAC  136
```

SNORD46 Prime5 Guide Sequence

> ENSMMUG00000024407|ENSMMUT00000034108|5S ribosomal RNA [Source:RFAM;Acc:RF00001]

```
Query  15  GCGTGGTTGTGGCCGT  30
           |||||| |||||||||
Sbjct  19  GCGTGG-TGTGGCCGT  5
```

SNORD67 Prime5 Guide Sequence

> ENSMMUG00000025475|ENSMMUT00000035176|U1 spliceosomal RNA [Source:RFAM;Acc:RF00003]

```
Query  17  GCCATGGTATC  27
           |||||||||||
Sbjct  30  GCCATGGTATC  20
```

SNORD67 Prime3 Guide Sequence

> ENSMMUG00000035367|ENSMMUT00000051101|U6 spliceosomal RNA [Source:RFAM;Acc:RF00026]

```
Query  11  TGCACAGGGGCCAT  24
           ||||||||||||||
Sbjct  69  TGCACAGGGGCCAT  56
```

SNORD87 Prime5 Guide Sequence

> ENSMMUG00000035174|ENSMMUT00000050908|Small subunit ribosomal RNA, 5` domain [Source:RFAM;Acc:RF00177]

```
Query  6    ATTACTTTTT  15
            ||||||||||
Sbjct  359  ATTACTTTTT  350
```

SNORD87 Prime3 Guide Sequence

> ENSMMUG00000024054|ENSMMUT00000033755|U6 spliceosomal RNA [Source:RFAM;Acc:RF00026]
Length=108

```
Query  3   TAATTTTAAGA  13
           |||||||||||
Sbjct  34  TAATTTTAAGA  24
```

SNORD94 Prime5 Guide Sequence

> ENSMMUG00000028323|ENSMMUT00000037934|U6 spliceosomal RNA [Source:RFAM;Acc:RF00026]

```
Query  4   GCGCAGGGGT  13
           ||||||||||
Sbjct  68  GCGCAGGGGT  59
```

SNORD94 Prime3 Guide Sequence

> ENSMMUG00000034845|ENSMMUT00000050579|U4 spliceosomal RNA [Source:RFAM;Acc:RF00015]

```
Query  1    CTGTTGCC  8
            ||||||||
Sbjct  130  CTGTTGCC  123
```

snosnR60_Z15 Prime5 Guide Sequence

> ENSMMUG00000034633|ENSMMUT00000050367|5S ribosomal RNA [Source:RFAM;Acc:RF00001]

```
Query  2   AACATAGT  9
           ||||||||
Sbjct  76  AACATAGT  69
```

snosnR60_Z15 Prime3 Guide Sequence

> ENSMMUG00000034616|ENSMMUT00000050350|Small subunit ribosomal RNA, 5` domain [Source:RFAM;Acc:RF00177]

```
Query  4   ATATTAAGT  12
           |||||||||
Sbjct  30  ATATTAAGT  22
```

snoU6-53 Prime5 Guide Sequence

> ENSMMUG00000034753|ENSMMUT00000050487|U6 spliceosomal RNA [Source:RFAM;Acc:RF00026]
Length=103

```
Query  3   TGCCATGCTAAT  14
           ||||||||||||
Sbjct  56  TGCCATGCTAAT  45
```

snoU6-53 Prime3 Guide Sequence

> ENSMMUG00000034328|ENSMMUT00000050062|Small subunit ribosomal RNA, 5` domain [Source:RFAM;Acc:RF00177]

```
Query  5    AACAAAACTT  14
            ||||||||||
Sbjct  571  AACAAAACTT  562
```

U8 Prime5 Guide Sequence

> ENSMMUG00000023832|ENSMMUT00000033533|5S ribosomal RNA [Source:RFAM;Acc:RF00001]

```
Query  12  TAATCAGGAC  21
           ||||||||||
Sbjct  65  TAATCAGGAC  56
```

U3a Prime5 Guide Sequence

> ENSMMUG00000033938|ENSMMUT00000049672|Small subunit ribosomal RNA, 5` domain [Source:RFAM;Acc:RF00177]

```
Query  4   GTAGTGTTCTTTC  16
           ||||| |||||||
Sbjct  81  GTAGT-TTCTTTC  70
```

U3a Prime3 Guide Sequence

> ENSMMUG00000025444|ENSMMUT00000035145|5S ribosomal RNA [Source:RFAM;Acc:RF00001]

```
Query  8   TCTCTCCTTT  17
           ||||||||||
Sbjct  97  TCTCTCCTTT  88
```

U3b Prime3 uide Sequence

> ENSMMUG00000035425|ENSMMUT00000051159|5S ribosomal RNA [Source:RFAM;Acc:RF00001]

```
Query  10   TCTCCATTTTG  21
            |||||||||||
Sbjct  113  TCTCCATTTTG  102
```

U3c Prime3 Gude Sequence

> ENSMMUG00000035425|ENSMMUT00000051159|5S ribosomal RNA [Source:RFAM;Acc:RF00001]

```
Query  10   TCTCCATTTTG  21
            |||||||||||
Sbjct  113  TCTCCATTTTG  102
```

U3d Prime5 Guide Sequence

> ENSMMUG00000024636|ENSMMUT00000034337|5S ribosomal RNA [Source:RFAM;Acc:RF00001]

```
Query  2   TAGAGAAGTTTCT  14
           |||| ||||||||
Sbjct  99  TAGA-AAGTTTCT  88
```

U3d Prime3 Guide Sequence

```
> ENSMMUG00000034208|ENSMMUT00000049942|5S ribosomal RNA
[Source:RFAM;Acc:RF00001]

Query  15  GCTTTGGAGA  24
           ||||||||||
Sbjct  52  GCTTTGGAGA  43
```

U3e Prime5 Guide Sequence

```
> ENSMMUG00000024636|ENSMMUT00000034337|5S ribosomal RNA
[Source:RFAM;Acc:RF00001]

Query  2   TAGAGAAGTTTCT  14
           |||| ||||||||
Sbjct  99  TAGA-AAGTTTCT  88
```

U3e Prime3 Guide Sequence

```
> ENSMMUG00000035425|ENSMMUT00000051159|5S ribosomal RNA
[Source:RFAM;Acc:RF00001]

Query  10   TCTCCATTTTTG  21
            ||||||||||||
Sbjct  113  TCTCCATTTTTG  102
```

U3f Prime5 Guide Sequence

```
> ENSMMUG00000024636|ENSMMUT00000034337|5S ribosomal RNA
[Source:RFAM;Acc:RF00001]

Query  2   TAGAGAAGTTTCT  14
           |||| ||||||||
Sbjct  99  TAGA-AAGTTTCT  88
```

U3f Prime3 Guide Sequence

```
> ENSMMUG00000034208|ENSMMUT00000049942|5S ribosomal RNA
[Source:RFAM;Acc:RF00001]

Query  15  GCTTTGGAGA  24
           ||||||||||
Sbjct  52  GCTTTGGAGA  43
```

U3g Prime3 Guide Sequence

```
> ENSMMUG00000034208|ENSMMUT00000049942|5S ribosomal RNA
[Source:RFAM;Acc:RF00001]

Query  15  GCTTTGGAGA  24
           ||||||||||
Sbjct  52  GCTTTGGAGA  43
```

U3h Prime5 Guide Sequence

```
> ENSMMUG00000024636|ENSMMUT00000034337|5S ribosomal RNA
[Source:RFAM;Acc:RF00001]

Query  2   TAGAGAAGTTTCT  14
           |||| ||||||||
Sbjct  99  TAGA-AAGTTTCT  88
```

U3h Prime3 Guide Sequence

```
> ENSMMUG00000035425|ENSMMUT00000051159|5S ribosomal RNA
[Source:RFAM;Acc:RF00001]

Query  10   TCTCCATTTTTG  21
            ||||||||||||
Sbjct  113  TCTCCATTTTTG  102
```

U3i Prime3 Guide Sequence

```
> ENSMMUG00000034208|ENSMMUT00000049942|5S ribosomal RNA
[Source:RFAM;Acc:RF00001]

Query  15  GCTTTGGAGA  24
           ||||||||||
Sbjct  52  GCTTTGGAGA  43
```

U3j Prime5 Guide Sequence

```
> ENSMMUG00000024636|ENSMMUT00000034337|5S ribosomal RNA
[Source:RFAM;Acc:RF00001]

Query  2   TAGAGAAGTTTCT  14
           |||| ||||||||
Sbjct  99  TAGA-AAGTTTCT  88
```

U3j Prime3 Guide Sequence

> ENSMMUG00000034208|ENSMMUT00000049942|5S ribosomal RNA [Source:RFAM;Acc:RF00001]

```
Query  15  GCTTTGGAGA  24
           ||||||||||
Sbjct  52  GCTTTGGAGA  43
```

U3k Prime3 Guide Sequence

> ENSMMUG00000035425|ENSMMUT00000051159|5S ribosomal RNA [Source:RFAM;Acc:RF00001]

```
Query  10   TCTCCAGTTTTG  21
            |||||| |||||
Sbjct  113  TCTCCATTTTTG  102
```

U3l Prime3 Guide Sequence

> ENSMMUG00000034208|ENSMMUT00000049942|5S ribosomal RNA [Source:RFAM;Acc:RF00001]

```
Query  15  GCTTTGGAGA  24
           ||||||||||
Sbjct  52  GCTTTGGAGA  43
```

U3m Prime3 Guide Sequence

> ENSMMUG00000035425|ENSMMUT00000051159|5S ribosomal RNA [Source:RFAM;Acc:RF00001]

```
Query  10   TCTCCATTTTT  20
            |||||||||||
Sbjct  113  TCTCCATTTTT  103
```

U3n Prime3 Guide Sequence

> ENSMMUG00000035425|ENSMMUT00000051159|5S ribosomal RNA [Source:RFAM;Acc:RF00001]

```
Query  10   TCTCCATTTTTG  21
            ||||||||||||
Sbjct  113  TCTCCATTTTTG  102
```
