# Supplementary material for: Systematic identification and evolutionary features of rhesus monkey small nucleolar RNAs
Source: BMC Genomics. 2010 Jan 25;11:61. doi: 10.1186/1471-2164-11-61 (PMC2832892; doi:10.1186/1471-2164-11-61)

Additional file 5

The expression pattern of rhesus monkey ncRNAs

Figure legend

The expression pattern of each ncRNA was examined by Northern blot with total RNA from rhesus monkey spleen, brain, kidney, liver, heart and skeletal muscles. Total RNA from human, mouse and chicken skeletal muscle were included in each blot in order to test the possible expression of each ncRNA in different species. Based on all the Northern blot data, the expression pattern in different species can be classified into six groups: group 1 (40 ncRNAs detected by 18 probes) shown in panel A, group 2 (51 ncRNAs detected by 49 ncRNA probes) shown in panel B, group 3 (16 ncRNAs detected by 14 probes) shown in panel C, group 4 (8 ncRNAs detected by 8 probes) shown in panel D. Group 5 and group 6 presented in panel E and F. All ncRNAs are labeled as Name_probe_size on the left of each Northern blot.

A

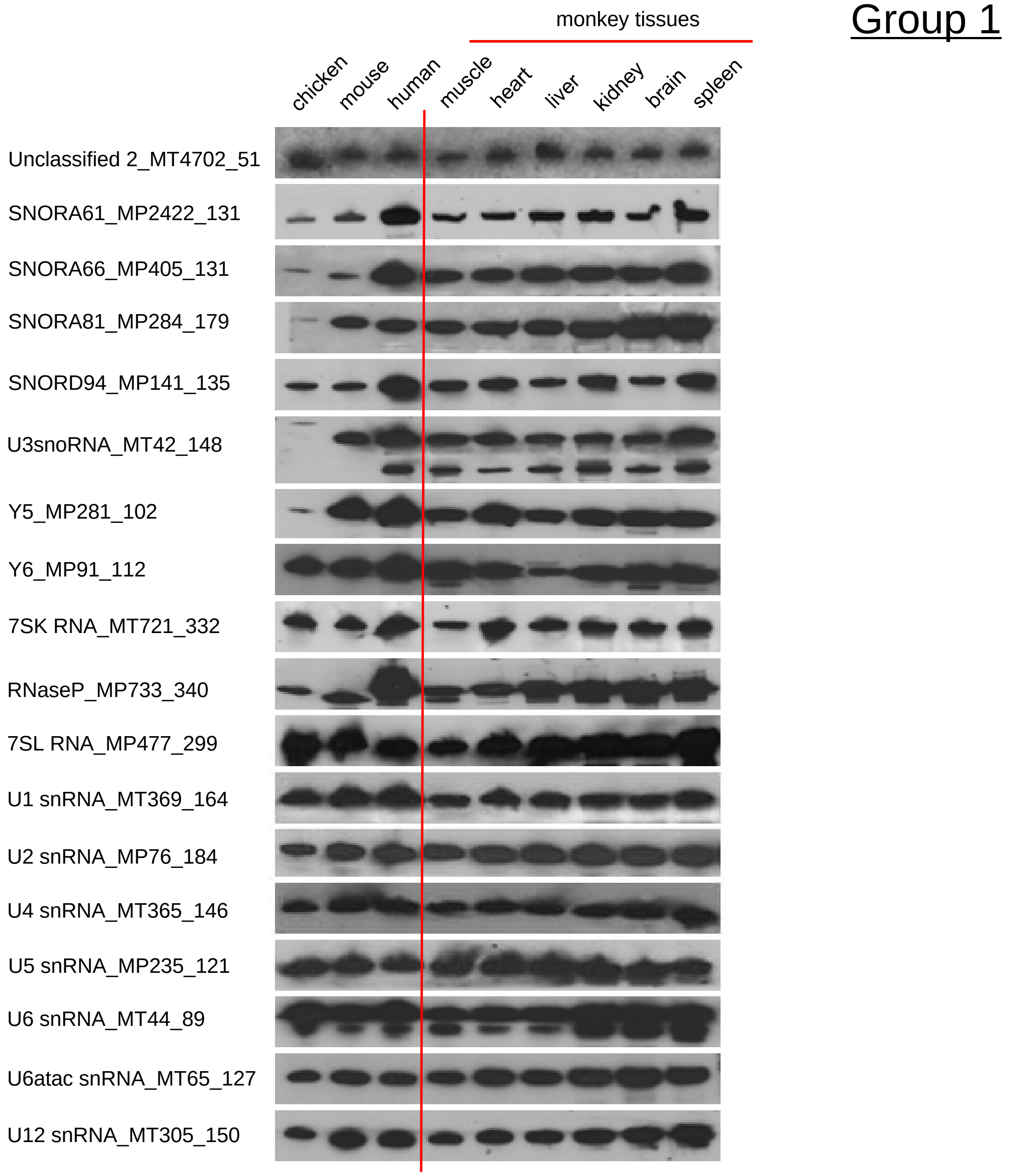

B

Group 2

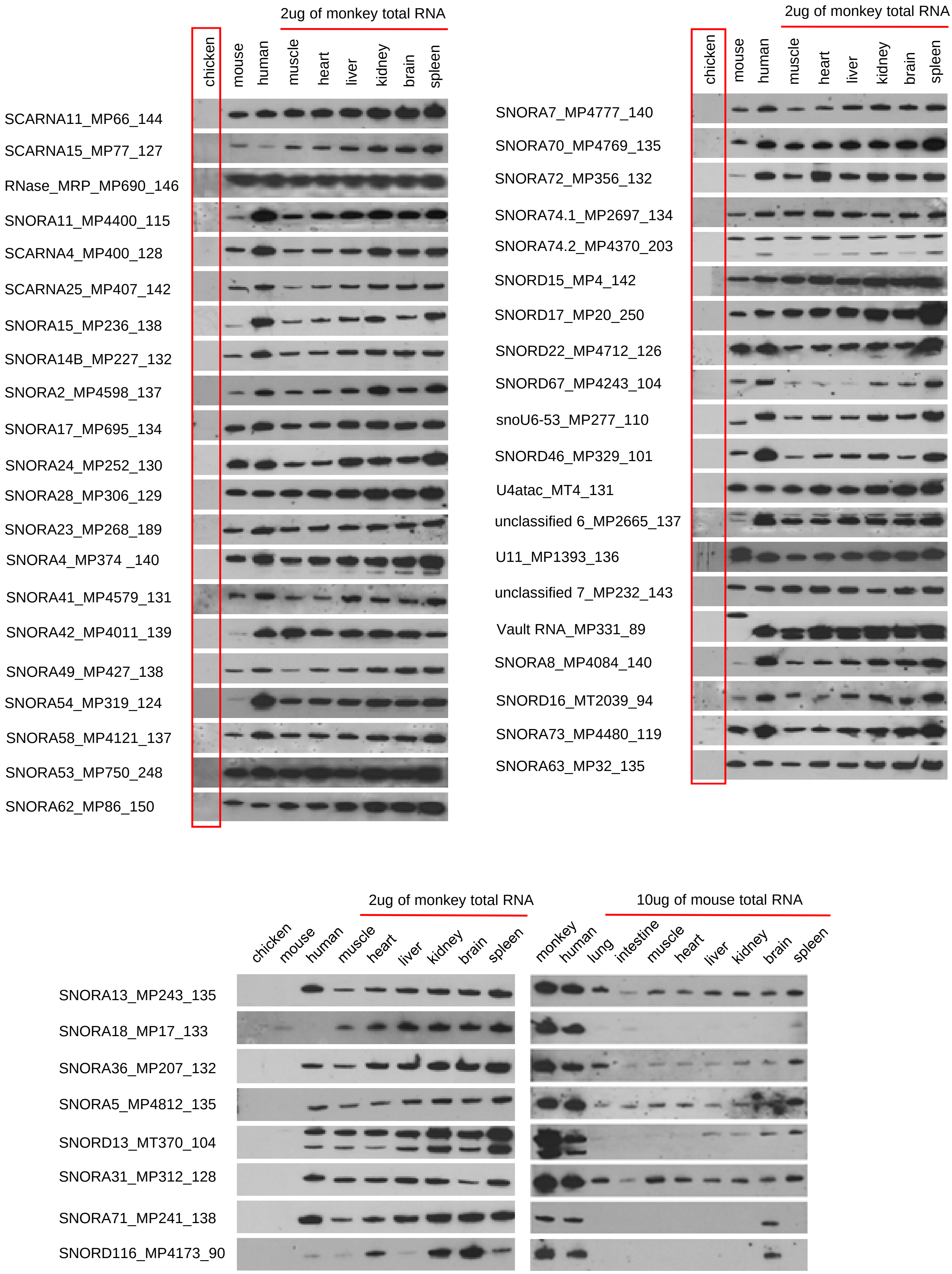
2ug of monkey total RNA
chicken
mouse
human
muscle
heart
liver
kidney
brain
spleen
SCARNA11_MP66_144
SCARNA15_MP77_127
RNase_MRP_MP690_146
SNORA11_MP4400_115
SCARNA4_MP400_128
SCARNA25_MP407_142
SNORA15_MP236_138
SNORA14B_MP227_132
SNORA2_MP4598_137
SNORA17_MP695_134
SNORA24_MP252_130
SNORA28_MP306_129
SNORA23_MP268_189
SNORA4_MP374 _140
SNORA41_MP4579_131
SNORA42_MP4011_139
SNORA49_MP427_138
SNORA54_MP319_124
SNORA58_MP4121_137
SNORA53_MP750_248
SNORA62_MP86_150
2ug of monkey total RNA
chicken
mouse
human
muscle
heart
liver
kidney
brain
spleen
SNORA7_MP4777_140
SNORA70_MP4769_135
SNORA72_MP356_132
SNORA74.1_MP2697_134
SNORA74.2_MP4370_203
SNORD15_MP4_142
SNORD17_MP20_250
SNORD22_MP4712_126
SNORD67_MP4243_104
snoU6-53_MP277_110
SNORD46_MP329_101
U4atac_MT4_131
unclassified 6_MP2665_137
U11_MP1393_136
unclassified 7_MP232_143
Vault RNA_MP331_89
SNORA8_MP4084_140
SNORD16_MT2039_94
SNORA73_MP4480_119
SNORA63_MP32_135
2ug of monkey total RNA
10ug of mouse total RNA
chicken
mouse
human
muscle
heart
liver
kidney
brain
spleen
monkey
human
lung
intestine
muscle
heart
liver
kidney
brain
spleen
SNORA13_MP243_135
SNORA18_MP17_133
SNORA36_MP207_132
SNORA5_MP4812_135
SNORD13_MT370_104
SNORA31_MP312_128
SNORA71_MP241_138
SNORD116_MP4173_90

C

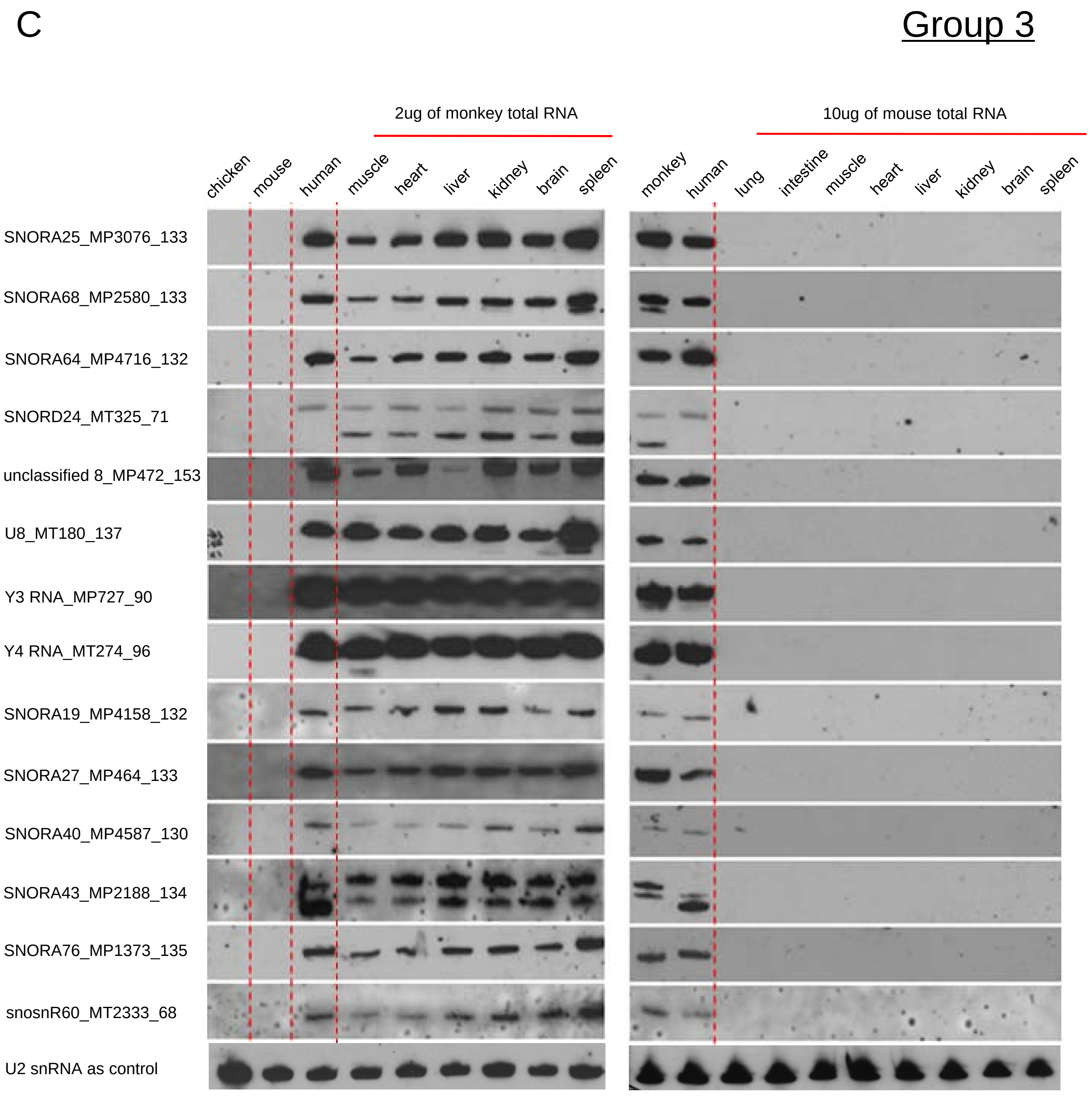
Group 3
2ug of monkey total RNA
10ug of mouse total RNA
chicken
mouse
human
muscle
heart
liver
kidney
brain
spleen
monkey
human
lung
intestine
muscle
heart
liver
kidney
brain
spleen
SNORA25_MP3076_133
SNORA68_MP2580_133
SNORA64_MP4716_132
SNORD24_MT325_71
unclassified 8_MP472_153
U8_MT180_137
Y3 RNA_MP727_90
Y4 RNA_MT274_96
SNORA19_MP4158_132
SNORA27_MP464_133
SNORA40_MP4587_130
SNORA43_MP2188_134
SNORA76_MP1373_135
snosnR60_MT2333_68
U2 snRNA as control

D

Group 4

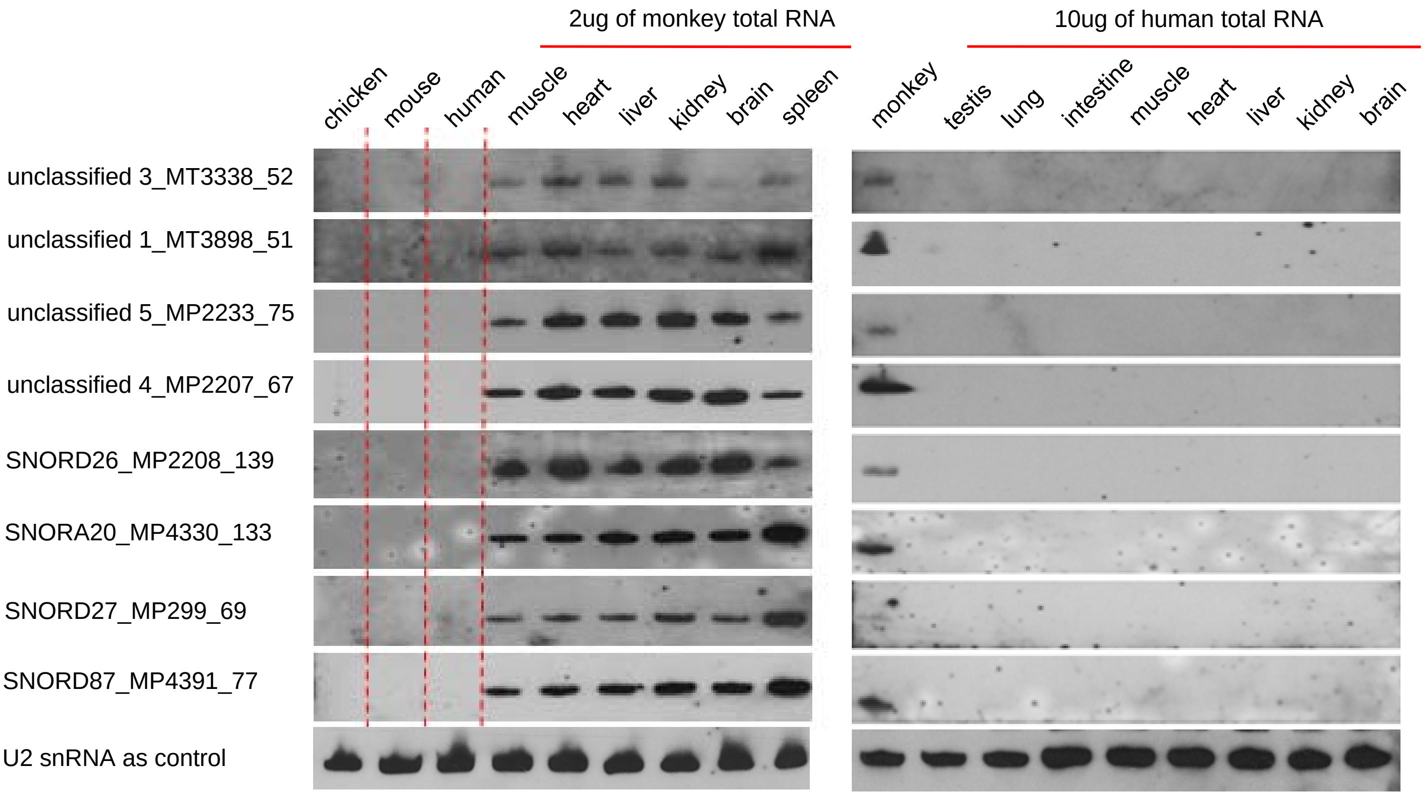

E

Group 5

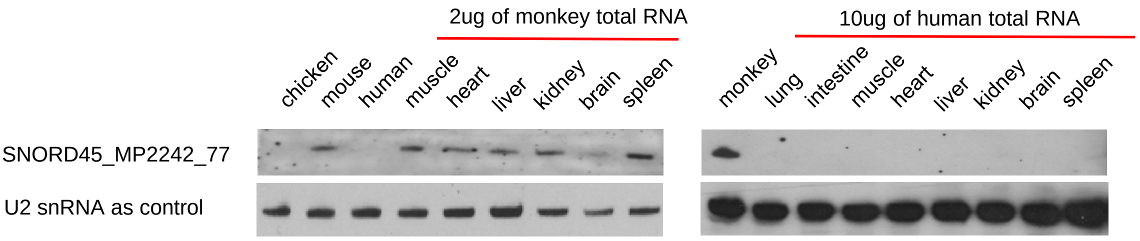

F

Group 6

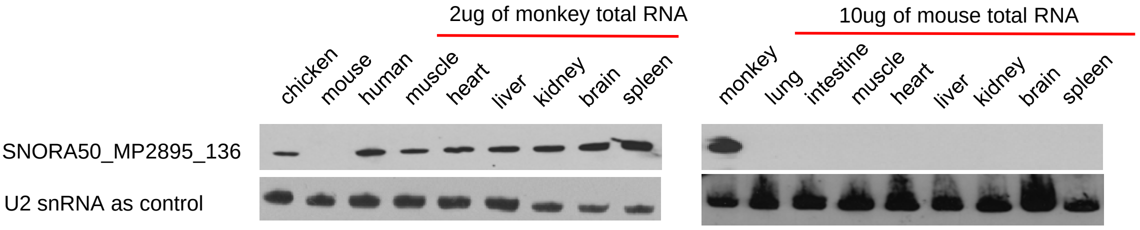

Supplement: Additional file 5 — The expression patterns of rhesus monkey ncRNAs. The expression pattern of each ncRNA was examined by northern blotting using total RNA from rhesus monkey spleen, brain, kidney, liver, heart, and skeletal muscle. Samples of total RNA from human, mouse, and chicken skeletal muscle were included in each blot to test the possible expression of ncRNAs in different species. Based on the cumulative northern blotting data, expression patterns in different species can be classified into six types. All northern blot data are shown in this file. [file 1471-2164-11-61-S5.pdf]
