# Supplementary material for: Systematic identification and evolutionary features of rhesus monkey small nucleolar RNAs
Source: BMC Genomics. 2010 Jan 25;11:61. doi: 10.1186/1471-2164-11-61 (PMC2832892; doi:10.1186/1471-2164-11-61)

Additional file 6 Sequences alignment of five rhesus monkey snoRNAs in the nine primate species

SNORA25: rheMac2 chr14(-): 92292766-92292899

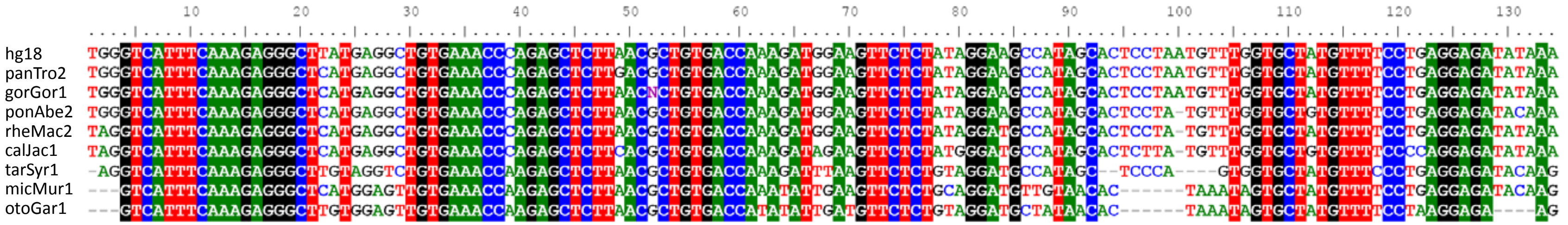

SNORA36: rheMac2 chrX(+):152763449-152763581

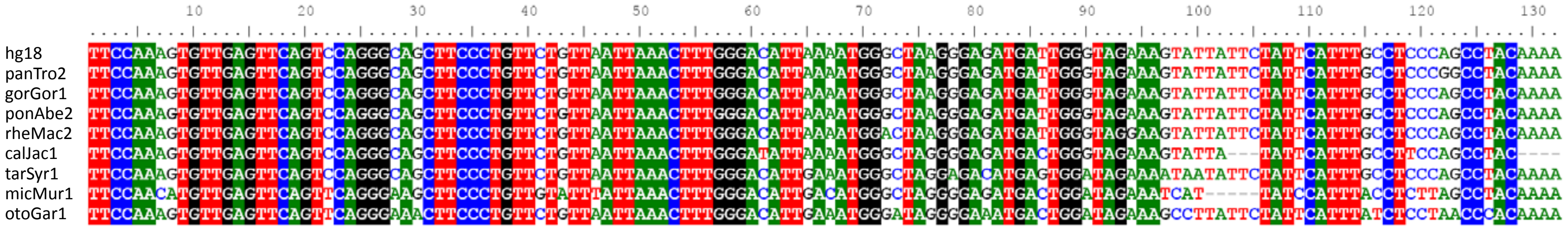

SNORA41: rheMac2 chr12(+):70040225-70040356

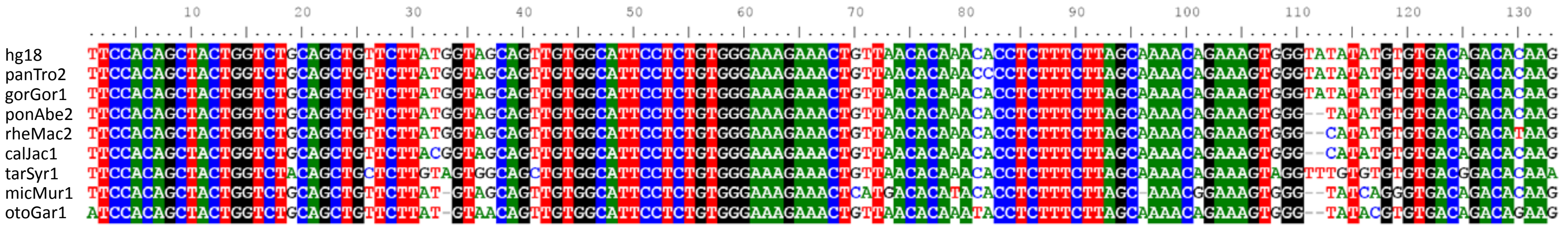

SNORA49: rheMac2 chr11(+):133339079-133339217

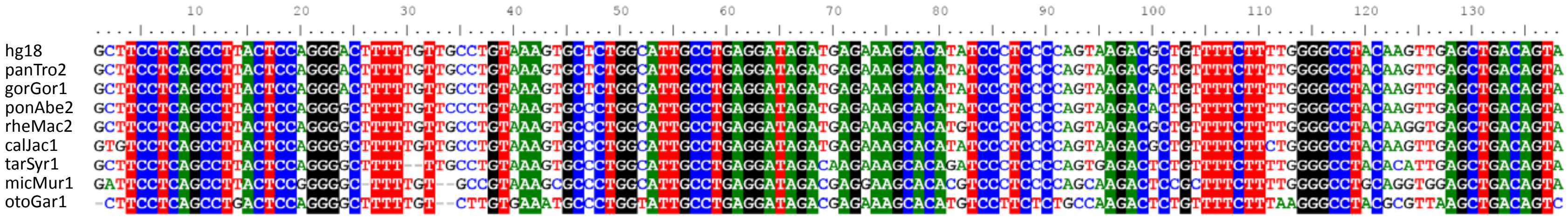

SNORA53: rheMac2 chr11(+):99784917-99785165

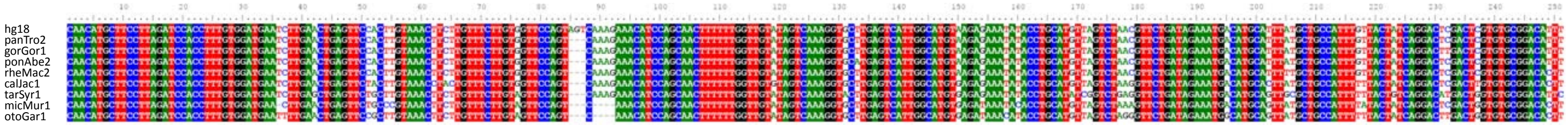

Supplement: Additional file 6 — Sequence alignments of five rhesus monkey snoRNAs in nine primate species. Multiple alignments of five snoRNAs in nine primate species are shown. The species analyzed were Homo sapiens (hg18), Pan troglodytes (panTro2), Gorilla gorilla (gorGor1), Pongo pygmaeus abelii (ponAbe2), Macaca mulatta (the rhesus monkey) (rheMac2), Callithrix jacchus (calJac1), Tarsius syrichta (tarSyr1), Microcebus murinus (micMur1), and Otolemur garnetti (otoGar1). [file 1471-2164-11-61-S6.pdf]
