# Supplementary material for: Systematic identification and evolutionary features of rhesus monkey small nucleolar RNAs
Source: BMC Genomics. 2010 Jan 25;11:61. doi: 10.1186/1471-2164-11-61 (PMC2832892; doi:10.1186/1471-2164-11-61)
Supplement: Additional file 8 — Twenty-two intronic snoRNAs with SINE-like retro-transposable elements. The sequences and features of 22 potential rhesus monkey snoRNA retrogenes were demonstrated. [file 1471-2164-11-61-S8.pdf]

Additional file 8
Twenty-two intronic snoRNAs with SINE-like retro-transposable element

snoRNA were highlighted as GREEN
direct repeats were highlighted as RED
poly-A,poly-T and AT-rich region were highlighted as YELLOW
The second repeats candidate were UNDERLINED

>CD_box_snoRNA_SNORD13.1
LOCUS: chr = 7,strand = -1,start = 44189486, end = 44190254

GTAGGTATTGTTTAAGTTGATATTGTATAAAATTGTGAACTTATATTCACAGAAAATGCAGGTGATTTTATAAAGTTCTGGTTTT
ACTTTCCATTTTTCCATCATAACTCTTTCTTGTCTCCCATTATGTGCCCCCTCAGCCACCACATACTCTCATACAAATGCTTTATG
TATGAAGTATATAGTCTCAGTGGCAAAACACTTCCCAAGTCAAATACCATTAGAATGAATGCTATGTGACTCCCAAAATACGTGTA
TTTAGTGGAAGTGTATATACATTTCTTGAAACAGTATTTCTTTTTTGAGATTTAACAATATTTGAAAGCTTGTGATCCTTTTGTAG
TTCATGGGCATGATGATTGGGTAGTCAAGCATGTTTGTGAGATGTGCCACCCTTGAACCTTGTTACAACATCAGCACATTACCCAT
CTGACCTGAAGAAAGAAAAAAAAAGTTTGTTTAATGGATTGTGATTATATTTACCCCCTTCCCCACTCCACCAAAAGCACTCCTTT
ACAATAAATGAGGAAATATAAAGTTTATACATGGACCTCATGCATGATCAGTATTCAGTAGTTTGATCTACACTTCAAGGCCTCAA
ATAAGTGAAATCACTTATTTGTGGTTGACAAACAGATATAGAGGTGTTCCGTATTTTCTTATCACCTGTTTTCGAGTAAAAATATG
TAGAACTGAAAACTTCTCTTGATACCTCCTACAGTGTATGTAGATTTCATTTTATTAATATTTTTGTTTTCATCTTTCCAG

>CD_box_snoRNA_SNORD45.1
LOCUS: chr = 1,strand = 1,start = 78589001, end = 78590223
GTAAGTGTGAGTCAACCGCAGCTGTCCTGATGGGTTGGTAGCAGACAGGGTAGGGTAGGGTAGGGTTGAGTAGGGTTAAGCACACT
GGTCACCTTAGGATTGGTTTCCTGGTGCTGGAGAATGATTAAGACACAGGCCTTGAAGATTTTTTGAGTGTGAAATATTACTCAGC
ATTTTTTGCAGACCTCGCGGGCAATGCCGCTTCTGATTTTATCCAGGCCTTCTGTAGGGAGGGCCTGTTAGAGTTGAGCAGCCCGA
TTTCTTAAACCCTCTAAAAAGCTGGCGCTGATTGGTGGCTTTTTTTTTTTTTTTTGGAGGGGGGATGTCAAAAATTTCTTTAAAAT
CGTTAGTGATGTGGTCTCGCTTTAAGATTTTTAGTGGGATCAGAGGAAAATTCGGTACTTAATCACTTCCGGTAATCTTCATGACC
TATTATTATGACTACTGTGTACTCTCAATGAGGAAGATAAAGATACGAAAAGAACTAGAGCCACATCACATGGGGACTTATGCAAA
TACAGAGACTCGGATTAAAGGTGGAGAAGATGGAGCTAAAGGAACTACTTATTTAATACATTTGAACAACTTTTGGGGTATTTAGA
AGGTGCTTTGAAACCTGCATTTGATAAGCAAAAATTCGCTTGCAAATTAAGGGCCAGTTAGATAAAAAAATATGTATATATGCTCT
CTGCCACATGTAAACCGTAAATTGTCCTGAAATGATAAGCTTTGATATTCTATGGTGTTTATTTTTTACTTACCTTTTTGAATGAA
AAGTGAACAACAAGCAATGCTGGTGGTAATTTTTTGGGTCAATGATGAGCTGGCATGTATTCTGAATCTAAAGTTGATTATTAATA
CTTTAGCTCTAGAATTACTCTGAGACCTGAAAATTACCTGAATCGTGACTAAGATGGAGCTCGAATGACTTAAGTTCTTTTTTGTT
AGATAGAACTTTTGTTTGTTTGTTTTGTTTTTGAGTCAGGGTCTCCCGTCGCCCAGGATGGAGTGCAGTAGTACGACCTCAGTTCA
CTGCAGCCTTAATTTCCTGGCCTCAAGCGATCTTCTACCTCAGCCTCCCTAGTTACAATTTTTAAATGGAACATTTTCCTATTACA
AGATTAAACGAAGTGAAAATTGAGATGTATGTTGAATCTGTGCTGTGGGGCAGAAGAACATTGTAGAGGTAAACATTGATAGATTA
CTGCTTTTTTATTTTGAAA

>CD_box_snoRNA_SNORD46.1
LOCUS: chr = 1,strand = 1,start = 47707377, end = 47707907
GTGGGTGCGAGCGTGGGCCTGTCCGCCCGGGAGGTCGCCTTCCCCGGCTCTCCAGCGTGCTCGGGCCTCTCCGTGTGACAGTTGTG
CGTTCTTTCTTGGGCTCTGATTTCTCTGTAATAGGGCCTGGGTGTCCTTCCCCCTTTACCTGCTGTATAAGTAAGTACCAAAGAGG
GAATGCTCCCTCAGGCCCCCAACCCCGGAGCTTAGGGTCTCAGAAGGATACTGTGTGGGGGACTTGAGCTTCTGGAGAGGGTGGCA
CCTCGGCTAATAGAATCCTAGTCTTTTGGGTTTTTTTTAACAGAGGCTTAATTTTTAGCATTTGGGGTCAGGCTTTCCTCTTGGAG
GCAAGTAAGGTGATGAAAAAGAATCCTTAGGCGTGGTTGTGGCCGTCTTGGTCACCTGTGTGCCACTTGCCAATGCAAGGACTTGT

CATAGTTACACTGACTGTTGCCTCCTTCCTGCCGCGGTTCCTCTCCCTTTCTTGCCTTGCTCTCCTTGGTAACCTAGTTCCTGTAA
CCTTGTGTTTTCCAG

>CD_box_snoRNA_SNORD67.1
LOCUS: chr = 14, strand = 1, start = 25356127, end = 25356559
GTGAAGCAAATAGTTTAAACTGAGATGTGAATCTCTGCGGCAGTAGCCTATAGAAGAAATAGTTTATAGATGAACAAATAAATTTC
TCCAATGCCTATGTAGTTGATTCCAACCTCCTGATTTAGACTGTGAGAGTGATGAGTTGCACACTGGTGGAGCCATGGTATCAGGT
GATACAGGCACCACTCAGTATCACCCTGGTGACAAAATCAAGTGCACAGGGGCCATCTGATTCACAGGCATCTCTTCCTTTCAGGA
GCACTTGCTGAAATACCATTTTTTAACTGATAAAAGGCAGTAAACAAAAAATCCTAGGCAGAGCCAGAGTCAGATTTAAGTCCTTC
ATATATGCCAAGGGAAATTTCTCTAGTAAGGATAAGTCTCTATCCCAAATATGTGTTGTTTCATTAATCTAGCCTTTCTCCCCCAT
CAG
>HACA_box_snoRNA_SCARNA11.1
LOCUS: chr = 11, strand = -1, start = 6732901, end = 6733161
GTAAGTCTGCCTGTCCTATCATTTTCTGTCCCTCTCTTTTGTCCTGGCCTATTTTTCTGCTCCCCTGTGCTCAGTTCTAACAGGG
TAGTCTGGCAGGACACACAGTAATTTCCCTCTCAGTTTAGGAGGGCCGTCCTAAGAATGGGGCTGGCTCTTAAAGGCACGAGAGGA
CAGTTTAAGATGAGACAAACTGGGAAGAAATGACCTCCTCTCTGATGTAGGACCTTCTTCCTAATTGATTGTCTCTTATTGTTCTG
CAG

>HACA_box_snoRNA_SCARNA4.1
LOCUS: chr = 1, strand = -1, start = 134500141, end = 134500981
GTATGTTTTGGGGAGGGATCATGAATAGCTTTATCATTTCTATAAAAATGATATGTGCCGTCCAGGTGCAGTGGCTCATTCCTGTA
ATCCCAGCACTTTGGGAGGCCGAGGTGGGTGGATCATTTGAGGTCAGGAACTCGAGACCAGCCTGACCAGCATGGTGAAACCCCAT
CTCTACTAAAAATACAAACATTACCTGGGCATGGTGGCAGGCGCCTGTAACCCCAGCTATTCGGGAGGCTCAGGCAGGAGAATTGG
TTGAACCTGGGAGGTGGAGGTTGCGGTGAGCCGAGACCATGCCACTGCACTCCAGCCTGGGCCACAGAGCAAGACTCCATCTCAAA
AAAAAAAAAAAAAAAAAAAAAAAAAGATGTGCCAAGTAATACATATAAAGAAGAAGAAAAAATCATTAAATTCCAATCCCGAAAGT
TACCTGCTATAGGTATTCTGTTAGTGTGTTATCTAGCTCTCCACAATATAATGAACACGTGAGCACATACCTCTTCATATTGATAT
GTTCTACAAAGGAGATTGATAGAGCAGAAGGTATTGTACATTTTGAAGTTAGTGCTTTTAGTTTATTTGTAAACTTGATAACTGGA
GGACTAAGAAGGCTGAGTCTGATGAAGCAAGACTTTGCTGATACATTCCTCCTAGAAAAAAGGGTTGGAGAGAGCAGCCTTCACTG
AAGAGTATCACAGGGCTGACTGTACTACCCAACACTCTATCCCAGTTTTACAGGAGACTGTCTTTGCTATAATTTCTTATTGACTT
ATTTCAAAGATTGAGGATTTAATTATATATTCGCTGTTTAATACGAAGTATTCTTTTCTTCCTACAG

>HACA_box_snoRNA_SNORA20.1
LOCUS: chr = 4, strand = -1, start = 156768797, end = 156769149
GTAAGAGCTACTTTCTAAAATTGTAAAGTTATACAGATTTTGTTAAGTAAAAGTAGGTTTTCTTCCCATTTATTTGCTGCTTATAG
TCTCACAGTGATATGAGCAGTTATACGCGTGGGATAAAATAACATTGGGTCACTGTGAATTGAGATGAAGTAACCATTTTCATCTC
TTCTGCATAGACTAGACATTGTTTCTGTATAGTGTATCTAAATATCTTGTGTAGGCAGGACTTAAGGGCACTTTATGTAGACTTAT
TAAAATGCCATTTTTTGGAGAAATTGATGGTGTTGGAAAAAAAAAAACATTTAAAAAATGCTATTACAAGAATTTCACTTTTTTTT
TTTGAACAG

>HACA_box_snoRNA_SNORA27.1
LOCUS: chr = 17, strand = 1, start = 7007747, end = 7008568
GTAAGTAGTGTTGTAGTTCTTTGCAGCCAACCGGTATTCCCTCATACCCCCTTTTCACTTTGCCAATTGGACTTATGTCTTTATTG
GTCATTCAAGTGGGGCAAAGGAAATAATCCTTTTAAAACTCAGGCAAACTGAGTGTTTGTCTTGTATCCTGTCAGAGGAAACAAAT
TGAAACAGATGTATTGGAAAGTCTTAACACAGTTTGTTACCAAGCAGTTTTTTTTGTTGTTTGTTTTGAGACAGAGTCTTGCTCTG
TCACCCTGGCTGGAGTGCAGTGACGGGATCTCTGCTCACTGCAAGCTCCACCTCCTGGGTTCATGCCATTCTCCTGCCTCAGCCTC
GCGAGTAGCCAGGACTACAGGCACCCACCACCATGCCCGGCTAATTTTTTTGTATTTTTAGTGGAGATGGGGTTTCACTGTGTCAA
CCAGAATGGTCTTGATCTCCTGACCTCGTGATCGCCTGCCTCGGCCTCCCAAAGTGCTGGGATTACAGGCGTGAGCCACCGCGCCC
GGCCCAGCAGTTTTTGTAGAATAAAAAGAGAAAATTTTAAATTTTATTAGCAATCTGGTTTGGGTTAGATTACTAGAGTTTAAGAG

GCTGTCATCTCATCAAATAGAGTTAAAAGTAGGGATGTTCTCTGCAAGGCTTCTGATATGATTAAATAATTGATTGTAAAGTAATC
AAGCCATACTTTTTTGATTTGTCGTATCTGGATAAAAGGTTCATGGTTTGTTTAATAAATGAAACTGCAAAAAGTTTTCTATATTT
CTGTTACATTTCTGATAAAGCATTTACAAGAATTTCTTCTCTGTTCAG

>HACA_box_snoRNA_SNORA31.1
LOCUS: chr = 17, strand = -1, start = 24256587, end = 24257861
ACAAGGGGGAGTGGGTTAAAATCAGTAATGTAAAAAAAAATGAAGATATTTTAGATGTGATTTCCAATTGTTTTGTGACACAGAAT
GAGTTTTACAGCATTCTGAAACATGGATTTAGTTTTCTCTTTGGGGATCAAGAGAATTGTGTTTCATATGTAAAAGATTCTTAGGG
TATAAAAAGGCTTAGGACCTTGTTTGTGGAAAACGTTGAGTGCAGATGGAGCATAATAAAGTACAGTTTAGGCTGGGTGTGGTGGC
TCACACCTGTAATTTCAGCACTTTGGGAGGCAAAGGTGGGTGGATCACCTGAGGTTAGGAGTTCGAGACAAGCCTGGCCAATATGG
CAAAACCCTGTCTCTGCTAAAAATACAAAAATTAGCCAGGCATGGCGTTGGGCACTTGTAATGCCAGCTATTCTAGGGGCTGAGGC
AGGAAAATCACTTAAACCTGGGACGGGGGTTGCAGTGAGCCGAGATCCCACCACTGCACTCTAGCCTGGGCGACAGTGAGGCGCTG
TCTCAAAAAAATAAAGTACAGTTTGAATGCTGAAGGAGATCACAGAACACCATTGATCTTCCTCTAGATATGGCCTCGCTTTCACT
TCATAATCATGTTTTGTTGTATACATATGGATCAGTATCAGTGTTTTTCACTTTGGTTTGCTGATAATTGGCAGCTGATCATTGAA
AGCTTAGTGCAGTACTAGCTTTTAAGTAAATAGAGCTGACTGCTGAACTGGTATGCAAATTGCTTTACTAATATATATAACTCAGT
GTCTCCTATGGAAGGAACTGCTGGAAGCTGTATAGAAACAAAGGCATTCTTTATCCTAGCTTTCATAGGCTTCTGTATAAATTAGG
AAGAAGATAAACATAAGGCTATACTGAACAAGCAAGATCGAGAGTCAACAATAGACAGATTACTTAGAACAGTGTAAGATGACTTA
CCAAAGGGGTTATTCAGAGAGTATCTGAGGTTTTTGTTGGTAGAGCAGGGTGTGGGTGGTACATGCCACAGCCTTCTGGAAAATGA
ACTACCGCTGATTTGGTAAGGGTATTCTGCATCCACTGATAGACCTTGAACAATTTACTGTTGTTCTTTTGGTTTGCACTAGGATG
CAAAAGAAAAATCCCTGCGCTTTCTGTCTGTCTTTGTGGCGGCCCAGATTGAATTGGGGAATACATCTATAGCCTAGAAATATAGG
CTATGTTAATGGTAATGTAATTTTTGCAATGTAACGTTTGAAAAACATTAATATAGTTTTTGTTTTTACAG

>HACA_box_snoRNA_SNORA36.1
LOCUS: chr = X, strand = 1, start = 152763355, end = 152763763
GTATGTGTTACGGGGCTAGAAGTTTTAGAGCTTGTTCTTAACATCTGCCAGCCTTTGGTGGCACTCCACTTGCTTCATGTTGTGGG
AAAGGCGCTTTCCAAAGTGTTGAGTTCAGTCCAGGGCAGCTTCCCTGTTCTGTTAATTAAACTTTGGGACATTAAAATGGACTAAG
GGAGATGATTGGGTAGGAAGTATTATTCTATTCATTTGCCTCCCAGCCTACAAAAATGCCTGCTTGGGATCTAATACTTCAGTGGT
TAAAGATGCCTGGAAGAGCCAGGACTGAAGAGGCTGAAGACATAATGAGCTTGGACTAGTAGACAAGTTACATTCAGTTTTAGGAA
GTTTGGGATCTGATGGGCCGAGATGCAAAAAAACTGAATTATTTTCATACATGGCTGCTTTTCAG

>HACA_box_snoRNA_SNORA4.1
LOCUS: chr = 2, strand = 1, start = 179145112, end = 179145331
GTAAGTCTCTTAATGCTTTTTAAAAATCTACCAAAAGTTAGCTTTTTGGGGGGAAGGTTTTTAAGTAACCTTTGCCAGCTTGGGCT
ATTTGGAAGAGTAAAAGGACCACACTCCACAGTGGGCTATACCACTTAGTATAGTTCGCTACTATTTTGTGGCCTACATGACAGGT
ACCATGTTTTTTGAATTAGAATTTTTAACATATGCCATTGTGTTTCAG

>HACA_box_snoRNA_SNORA49.1
LOCUS: chr = 11, strand = 1, start = 133338045, end = 133339836
GTGAGAAGGACATTGCTTGCCTTGTCCCTGATGGGTCAGCCACCGGTGTGTAAGGCCTGAGTGCTGTGTAAATTCAGTGGTCTCAT
GGCCCTTCTAAGTGAAAGGAGAAGGACTTAATGAGCCTTTGAACTTCCTTAAGTCTCAATCCATCCTTTGATATTTTTTATTGAAA
GCCATGTTGTTAGGTAAATGCAGTCTTGATGTCTCAGAATAAACACTGGTGACCTGCAGTCATCGTTAAGACAATACTGGTGTACT
TCAAGAGGTAACTACGGGGTAAAAATATGAAAACAATTCCAATCAATTTAGAATTAAAGGAAATACAAGTTAAGATAATATGCCCG
TTGTTTACTTACTAGATTTGCCAAGGCTGTTTTGATTCATCAGGCACAGTGAGTCAGACACTCTCACTGTATAGCCTGCCGTGGAG
GCATAAACTAGTATAGTTCTAGAAGGCAGCCTGGCAGTATGGATTTGAACCTTAGTGGTAATGTATTTAGAACCCTTTACTTACAT
AACAACAAACACACCACGTGAGATCTGTCTGCAGAGTCGTCGTTGAAATGTTGATGGTAATCCACAGCTGGGAACAGTCAGTGTTC

AGCAGAAGAGTGAGTGCATAAGGGGTTGTCCCTGGGCCCCGCGCCCCGGCAGCTTCATGAGCAGAAACCGTGCCCAGCATTCCATG
TGGTGCGCTAGGCCCTTGTCAGCAGCATTTCAGAGCCAGCTTCAGTAGCATGGCCCCGTGTCCCCCAGATGCTGCCCTGTAAGTCA
CGCGACCTGCTTTATCTCACACACATGCTCATGCACACGCTGAAGAGGGCTAGAACGAAATAGAGCAAAAGGTGGTTTAACAAGTT
TTCTTGGTGATAGTCTAACAAATTACAGGCAGAACGCTTTGCAGGAAGAGAGATGTTCAGTGGTTAGGTTTCGTTTCCGGCACAGA
AATAGTTATGGAAGAGAAGTATTCAAGTCATCTCCTTTCATGGTCCTCAGTGAGCTTAGAGGAATGTCCTTCACGGTTTTATTTGT
CCTGCTTCCTCAGCCTTACTCCAGGGGCTTTTTGTTGCCTGTAAAGTGCCCTGGCATTGCCTGAGGATAGATGAGAAAGCACATGT
CCCTCCCCAGTAAGACGCTGTTTTCTTTTGGGGCCTACAAGGTGAGCTGACAGTAAATGCAAGGTGGAGTCTAACTTTTTCTTGTC
ACTCATGGCTGGTGTTATTTAAACCGTGGGTATGAGGGTGTTGGAGATGGGTGAGTGTGAGATGATGGTGGCTGCACGGAAGAGCC
ATAATACAGTGCAGGGACCTTGGTGTTGCTTTAACATGAGCCTTGTGTTGAATGGAATACAAACCAAGCAGTTTTGCTATGCGATA
ATTTCTTTTTCTTTCTTTCTTTTTTTTTTTTTTTTTTTGAGACAGAGTCTCGCCCTGTTGCCCAGGCTGGAGTGCAGTGGCGCAGT
CTCGGCTCACTGCAAGCTCTGCCTCCCGGGTTCACCCCATTCTCCTGCTTCAGCCTCCCAAGTAGCTGGGACTACAGGCATCCGCC
ACCTCGCACGGATAATTTTTGTATTTTTAGTAGAGACGGGGTTTCACCATGTTAACCAGGATGGTTTATATTTCCTACCTTGTGA
TCCGCCCGCCTCGGCCTCCCAAAGTGCTGGGATTACAGGCGTGAGCCAGCACACCTGGCCTGCTATGGGATAATTTCTAATTGATA
AATGGTGAAATGTGTCAAAGGTTTCCCAACATTTTCCAGGAATACTGACTCCAGTGTTTTGTTTCATCTTAG

>HACA_box_snoRNA_SNORA5.1
LOCUS: chr = 3, strand = 1, start = 81243012, end = 81243290
GTGAGCCCTTTGGACAGAACAGTGGAGAGAGGGGAGCTCTGGCCCTGCAGATACCTGCAGCCATGTCAAATTCAGTGCCTGTCCTA
TGCATGGTAGGCACTGGCCCAGAAGGCTGCCACAGAAACACTGTGACTCATGGGCCCTGTTCCTGGGTCCCAGGCTCAGGGATAAA
TTTGGTTACAGACATCAAGCAGTGAGACCTTGAGGCTGAGGTGGGGTGAGGCTGGTGGGATTGCTGACCCTCCGCAGCTAAACCCT
GTGATCTGCTCTGTCCCGAAG

>HACA_box_snoRNA_SNORA53.1
LOCUS: chr = 11, strand = 1, start = 99784304, end = 99785235
GTAAGTAAACACTTAAAAATTTATATTATGAAAGTACTTATTTTAAGTGAACTTCATTTTTTTTGAGACAGAGTTTTGCCCTTGTT
GTCCAGGCTGGAGTGCAGTGGCACTGTCTGGGCTCACCGCAACCTCTGCCCCCCCAGATTCAAGTGATGCTCCTGCCTCAGCCTCC
TGAATAGCTGGGATTACAGGCATGCACCACCACGCCTGGCTAATTTTAGAGACTCCATGTTGGTCAGGTTGATGTCAAACTCCTGA
CCTCAGGTGATCCACCCACTTCGGCCTCCCAAAGTACTGGGATTACAGGCGTGAGCCACTGCGTCCAGCTGTGAACTTCATTTTTT
AGTATTAAGTCTACCAGACTTTTTAAAAAGACTATTATAACATTTAATGTCTGTTTACCTGTATGTAGAACATAAACTTGAGAGGT
AAAGATAAGTTAATACATGGTTTGACCCCTGGACTGGGGTTTTTTTTTTTTCCCCCCCTAAAGTAAGGACCATTTGTCATTCTGCA
AATGACTTGATATTTGGCCTAAACATTTACTATTAACTTTTAGGGAGGGCTTGTTAGTATCCTTGTGATCTATTCACAGTTAAGAA
TTTCCATGACTACAACATGCTTCCTTAGATCCACCTTTGTGGATGAATCTTGAACTGAGTTCCACTTGTAAACTTCTTGTTTCTTG
TGGTTCCAGTCAAAGAAACATCCAGCAACTTTTTTGGTTGTATAGTCAAAGGTGCTTGAGTCATTGGCATGTAAGAGAAATATACC
TGCATGTTAGTCTAACGTTCTGATAGAAATGACATGCATTTTTGCTGCCATTTGTTACTATCAGGACTCGACTCGTGTGCGGACAC
TTGTGTTAATAATGACAATCTCTGATAGATGAGTATATGCAACTCTGTAAAACAAGTCTCTTGATTTCCTAG

>HACA_box_snoRNA_SNORA58.1
LOCUS: chr = 1, strand = 1, start = 132787209, end = 132788238
GTGAGTATGTGGGATAGAGCTTTGGAAAATAAAGTAGTCTTAAAATTAGTGTTGGGAGAATTACCTCAGTCACAGACATAGCTCTT
TCCCATCCCAGCCCTCCATACACTCTGATCTAAGGCATATTAGAATATCGGGATTTTGGGGAGGGTTTCCAATAGAAGATAACTTT
TTTTTTTTTTTAAAGACAGAGTCTCACTCNNNNNNNNNNNNNNNNNNNNNNNNNNNNNNNNNNNNNNNNNNNNNNNNNNNNNNNNN
NNNNNNNNNNNNNNNNNNNNNNNNNNNNNNNNNNNNNNNNNNNNNNNNNNNNNNNNNNNNNNNNNNNNNNNNNNNNNNNNNNNNNN
NNNNNNNNNNNNNNNNNNNNNNNNNNNNNNNNNNNNNNNNNNNNNNNNNNNNNNNNNNNNNNNNNNNNNNNNNNNNNNNNNNNNNN
NNNNNNNNNNNNNNNNNNNNNNNNNNNNNNNNNNNNNNNNNNNNNNNNNNNNNCAGGCTGGTCTTGAATTCCTGACCTTGTGAT
CCACCTGCCTCTGCCTCCCTAAGTGCTGAGCTTACAGGCGTGAGCCGCCGCACCCAGCCTTTTTTCTTCTTTTTTTAAATCAGAGT
TGTGGCATCTTAGTAAATGCCATTTAACCTAATCTATAGCCTAGTAAAGTAATCTGGAGCACTCAGGGTGCTTTTTGTTTGTCCCA
GTCACTTTAAATTACAGAATGATCTGTTTTCTCAGTAGAATAAGCTTGGTATAAACTGATAGGGATGACATCTAAAGTGCTGAAAA
TATTGGTCCTCATGGACTTTGACATATTCTCAAGTGGGCATACCCGTAGACCTTGCCTGACTGTGCTCATGGCCAGGCAAGGGGGA
CAGTGTATGCAAGAGTAATGTGGAGTTTGTGCTAACTCTAGCCAGCTTAATTAATGACTGGATAAATTGCACAACTCTCACATTCT

TCTGTTCTTTCCTCTGTGGAAATAATACTCATGCTGGGGAATGAGTGACATTGAATGTCTGCTCTTTTTTTCCCTCTTTTCTAG

>HACA_box_snoRNA_SNORA62.1
LOCUS: chr = 2, strand = -1, start = 97131619, end = 97132444
GTTAGTCATTGCTTTAATTTTTTGTTACTCCAGCTGTAAGTACAGATTTTGAGCTTGCTTATTCTCGTGGTTAGTTCTGGGTAATT
TCTTTGTATCATCTCTTCCTCAAATGAAACCAGACTCCTAGGTTGAAAACATACTTTAAATAAATGTTCTGATTGTTATTGGGAGA
AAAGATTCCTGCTCCAGGGGAAGAATACATCAGTTGCCCGCGTTATGGGACTTGGGTTGGGTTGTGAACTTGAGCAAGTTCATGAG
CTGAGTTTCTAATAGCTCCCATAGTGCCTTCAACTTCATAGTTACTTACACAGGCTGTTGAACTGAATTTTGAGTGGAAAGTGGGG
CAGGAGGTGGGGATAAACAATATAGCTTGGAAGAACCAGAGGAGGGAAGGCATTAAGTTGGCTAAGGCACTTATTTTAGAGATAAA
GCTACCAAGGACTTCGGAGTGACATGCTGTGAAGGAGATTTAGGATGAATCTTGCATCAGTGAAGATGATAAAAGGGAAAGAGTGG
CAGAAAGTCAGGAAGTGGATTCTTAAGAACCACTGATGGACAGGAAGTACAGGGAAGGAGAAAGGAGAGATGGATTTAGCCAACTG
AATCTCGGCTGCACACTATTAAAGCTCAGGGTGGAGGCCAGTCTTGGCTCATGAACTTCTGAGTGTCGGAAGTGTGCTACATTAAT
GGCAGGATTTTCGCTAACACCAGTAGAGCTTGCCTCTATGACTGGAGTTTGGTAGTACTCGCTGCCACATACACTGAACTTGCAAA
GAATGATTGCACAGCCAGGTCAAGTGTTACAAATCCTTTTGCCTTCGCTTAG

>HACA_box_snoRNA_SNORA64.1.
LOCUS: chr = 13, strand = -1, start = 57045323, end = 57045548
GTAGGTGGTCCAGGAATGGGGCATAGCCATGGTCTCTCAGTTCCGCTTAACCACACGGGTCCGGTGTGTGCTTGGCGTGTTTTCAC
GGAGGCAGAGAAAGGCTCTCCTAATACACGACAGACCCGCTCAGAATGGCCTCTCTGTTCCTAGGAGTGCGACAATTTTGGGGTTG
GGAGACTTGCCTCAAGCACACCACTGACCCTCCTGGGGTTCTTTTTTTGTGCAG

>HACA_box_snoRNA_SNORA7.1
LOCUS: chr = 2, strand = 1, start = 48204399, end = 48205614
GTAAGTCTTAATCCGTGGCCATCTGCATTCCTGCGGGATTCATCTGGCCCCATTGCCCAGTGGTGCGGGGGCCTCCCCTTCGGCGC
GGTAGTGGCTGTGGGTATTGTTATTGTCAGCTTACTGGGGCGTGTACAGGAGCAGAACGAAGCAGCCCGGGTTGATGGGGCTTTGC
GTCCCAGAGCCTCCTGCTCTCCGCTTGTAGTCAGAGCTGCGGGCTGCTTGTTTGTTCCTTGGCGGTGGAGGGTGCTAGTTGAGCCC
AGACTGCGGGGTCTCCTGTGGGCCGTGGGACGACCAGGGGTGTCCCAGCTTGACAGCCGTCAGCTGGGATCTGTGGATCCCAGCGC
TCACCGATGTCGGCCCACGTGTATTCGTTCATTGCTATAGCCCGCTTCTTCGCTGCAGTCTCGGGCCCTAGGGGTGCTGCGGCTGC
TGGTTCAGTTGCAGGCAGAAATTCTGGTAGTATCTCTGGAAATAAGATGCAACTGCCCCCACCTTGCCTTCGAGGATATCATGGGC
CAGAAGGCAAAGTCGTTTTGAATACGTGGTTCACTGATTACCCACTCTGTGCCAGTTGATGGCTGCGAACAGAAGGGGTGCTGCTC
TAGGAAATAAATGAATGGCTCTGAAGACCACACTGAGGAAGGTGTGAGTTGATACTGCAAGATCTCCAGGTTTGAGGCATCCTTAG
ACGGATATGATGGTTTTGTGTGTGCTGAGGGTGTGGTAGCGCAGCAGCTCCCTAGGGAATTAGAAGGTTTTCTTTATTAAACGTTT
ACCCTGTGACAGGCACTGCAGGCATTCAGCGTGCAGTGTCATCTCTATTCTACAGGTGAGGAAACTGAGACTCGGGTCCAAGTAGT
TGGTCAAGGCCATCTTGGGGTTCGGACACTGGCGAGGTGGGATTTGCTGCCCCTTGCAAATTGAGTGTCTTGGGGTCGGTTTTGAT
TTGCTCAGCTGTTGGCATTCTTTGGGCTCTGAGTGGTTGAGTTGACCTTTGACCTCCTGGGATCGCGTCTGGAGAGTGCCTAGTAT
TCTGCCAGCTTCGGAAAGGGAGGGAAAGCAAGCCTGGCAGAGGCACCCATTCCATTCCCAGCTTGCTCCGTAGCTGGCGATTGGAA
GACACTCTGCGACAGTGTTCAATCCCTGGGTGGGAAAACCTCCTTCCAAGCCGCCTTCCAGGATTTTTCCTCACCTGGGGCTGCTT
CTTTCCCAGAAG

>HACA_box_snoRNA_SNORA72.1
LOCUS: chr = 8, strand = -1, start = 100581879, end = 100582981
GTATGCTTTTTAAATCATAAAGTCTTTTTTTTTTTTTTTTTTTTTTTTGCCTTTCTAATGTAATGTCTGGACAAGTATTTCTTACT
ACGCAAATGTCTTTGGTTCCTAAATTCAGATAGAATATGGATATAGCAAGGCAAATCCTGTAGTTGTCTTTTGTAGCAAATTGGTT
ATGAATGTCAACACATTATAATTGGCTTTATTATAAATTAAAATTTTTACTGTCAGATAATAAATTTCATTTGATAAAGTACCTGT
GCGATAATGACTTAGAGATTTGGTAAAAGGTGAAATTATAAGGGGAGAATAATCTGGCAAAACAGAAGAAAGTAATTCTTGCTTCC
AGAGAATAGACATGTAAAATTAGCTTGAGGAAAAAAAAAAGTGCCAAAAAATCTACATCATTTTGTTTTCCAATTGTGAATCTTTG

ATTCATAGAATACTGCGAATATTCTCGCTGTTCTGATTTTGTAATAGTCAGGACAGGCTAAACATTCGCTATATTAAGACCATGCG
TGTGTCCCCAGACCTAGTTCTTTCCCTAGGTCTGGTTTTATAAATGCTGGTGATAAACCAATTTTATTATATGATCGTTTTGTGTG
ATCATATAATGGCTATGATTCTGGGTTATCTTTGATTAACCTTAAGAAAAATGACAGTACAGGTAATGTGATAACTTTTGCAAAAA
ATCAATGCTGGCCGGGCGCGGTGGCTCACGCTTGTAATCCCAGCACTTTGGGAGGCCGAGGTGGGCGGATCACGAGGTCAGGAGAT
CGAGACCACGGTGAAACCCCGTCTCTACTAAAAATACAAAAAATTAGCCGGGCGCAGTGACAGGCGCCTGTGGTCCCAGCTACTCG
GGAGGCTGAGGCAGGAGAATGGTGTGAACCCGGGAGGCGGAGTTTGCAGTGAGCCAAGGTCGAGATCGCGCCACTGCACTCCAGCC
TGGGCTACAGAGCTAGACTCTGTCTCAAAAAAAAAAAAAAAAAATCAGTGCTAATTTGCAGACCATTGTCTACAGAAGGGATAACCA
AAGGTAGAGAAATTTTATGGTACCAGTTGCTATTAAATAAATTGTAAAATTACTGTATTTTTATTTTTTAG

>HACA_box_snoRNA_SNORA74.2.nc128.c_1.sno.flank.
LOCUS: chr = 6, strand = 1, start = 135689748, end = 135690384
GTAAATATCCCTGTTCATTCCAACATTGACTACCTCCTCTGTGCCAGGCCTGTTGGCTAGTTGGTATCTATCAGGAAACAATTGAG
AGGTAAATTAACCTATATTTGTAAGATCCATGAAAGAAAGGACAGTATATCTTGTTTTATTCACTGCTAGATCCCAATCACAGTGC
TTGGCACATGGCAGGAACTCCCTGAATGTTGGATAAAACAACTGAAAACATGGAAGGGGATTCCTGTTAAAGAGTTTGACAACAGG
GTCTGCAATCAGTGCTTCCATGCCTGTTTTGCAGCCAACATTCGACTGATAGGTCTTCTACCTGGAGTGCCTAGGTATCTGAAACA
ATGAGAAAGCTGTTTCAGGAATCATCCAGCGGTTGTCAGCTATCCAGGCTTGTGTGGTGCCTGTGATGGTGTTACACTGTTGGAAG
AGCAAGCACTGTCTTTATTGAGGTTTGGCTCCAAGCACTGTTTTGGTGTTGTAGCTGAGTACCTTTGGGCAGTGTTTTGCACCTCT
GAGAGTGGAATGACTCCTCCTGTGGAGTTGGTCCTAGTCTGGGTGCAAACAATTTTTTTCTGTTAGAAAAAGGACCCTGTCTTAGG
GACAGCCTGTCATTCTTTTCCTCTTTGAATCCTAG

>HACA_box_snoRNA_SNORA8.1
LOCUS: chr = 14, strand = -1, start = 92294655, end = 92295444
GTAAGTAAAATATTTAACTTTTAATAATCAAGGACTCAAAAGATGAAAAATAGAAATTAAGTACACCATCCCAGTATTTCAGGTAT
AACACAGAATTAGTAAGATACTGGCAAAAATGTTACTATATATTTGTATAGAGAAGGAAAATGAAGAGACTGCATGTCTAGACCTA
CCAAATGAAATTACCTGTGTTCTTTGCATTGTTATTGAACTGGCAGTTACACATACTTCATCCTAAAGTCACGTAAACCTGTATGG
ATATGTTGAATCAATAGGGATATGAATTACATTAAAAGAGGTTTGTGTAACTGGCAGTTTGAGCAACAATGAGCTTGACATTTGTA
GTCTGCTTGATCTGTTAAGTTACATGTATATATTCCATTTGTAAGTTATATGGAGACTGGGCAAAGATTAGGAAAAGACTAGAGTT
GTAGCTTAAAATACAGTTAATGCAGCTTATTACAGTGACAATTAGGAAAAAAGGTCAAATTATTGTACTTGTTAAAGGAACTTGAA
TTTGAGAGGTTTGTGAAAGCTTTTCTCAGTAGAAACATTGCACATTTGGGAATACGAATATGAATATGAATGTGACAGTTCAGTCA
TAATTGCACTGCATGGTATCTGCACTCAGCAGTTTACTCCTGCTAGGGTGTTCAAAGGTCAGTGCTATAGAAATTCAGTATCTGGC
ATCGTTGGTTTTCTTGGCTTTGTGCTTGTTAAACCTGGTATTTCTATTGATACAGTATTTGTATAGGTTCCTCAATGTTCAGTTTG
ATATACTCTAAAATAG

>HACA_box_snoRNA_SNORA81
LOCUS: chr = 2, strand = 1, start = 179144197, end = 179144657
GTAACTGATTGTTAGACATTATTTTACCTTCTTGTATAAGCACTGTGCTAAAATTGCAGACACTAGGACTATGTCTTGGTTTTTGC
AATAATGCTAGCAGAGTACACACAAGAAGAAAAGTAACAGCACTAGATTGTAAAGACTGGGATGGACCTCTTTCTTAATGTCCAAT
GTCCTTTGTCTTAAGATTTGGTGCAATATCTCATTAGACCTGATAGAATGAAGTTAATTTTTATAATTTCTACGTTTTGAGACTGG
CTAAGAGGGGCCAATTTTGGTATTTTGGGTAGAGACAGGGTTTCACTATCTGGAAACTGGTTGGAACTCCTGGGCTCAAGTGTTGG
GATCCCGAAGTGCTGGGATTATGGGCATAAGCACTGTGCCTGGCCCAGAATGTATTTTTAACTGAAGAAAGTTGGAAAAGCTAAAT
GACTGTTAACTTTCTCTTCTAAACATTATAG
