# Supplementary material for: Systematic identification and evolutionary features of rhesus monkey small nucleolar RNAs
Source: BMC Genomics. 2010 Jan 25;11:61. doi: 10.1186/1471-2164-11-61 (PMC2832892; doi:10.1186/1471-2164-11-61)
Supplement: Additional file 9 — All oligonucleotide sequences used in this study. All oligonucleotide sequences used in this study were shown, which included the sequences for adapters, primers and probes. [file 1471-2164-11-61-S9.pdf]

# Additional file 9 All oligo sequences used in this study

(Underlineds: RNA; *Italisc*: Restriction Endonuclease site; p.: 5’ phosphate; -x: 3’-DMT.)

>5AD: 5’ adaptor

GGAGUA*GCAUGC*GUGACGAAAA

>3AD: 3’ adaptor

p.UUUUGACCAC*GAGCTC*ACAGGG-x

>5CD: 5’ PCR primer

GGAGTA*GCATGC*GTGACGAAA

>3RT : 3’ reverse transcption & PCR primer

CCCTGT*GAGCTC*GTGGTCAA

>5S-1pA: probe used for removal of 5S rRNA

AAAAAAAAAAAAAAAAAAAACGAGATCAGACGAGATCG

>5S-2pA: probe used for removal of 5S rRNA

AAAAAAAAAAAAAAAAAAAAGTCTCCCATCCAAGTACTAAC

>5.8S-1pA: probe used for removal of 5.8S rRNA

5’-AAAAAAAAAAAAAAAAAAAAGAGTGATCCACCGCTAAG

>5.8S-2pA: probe used for removal of 5.8S rRNA

AAAAAAAAAAAAAAAAAAAAACCCTCAGACAGGCGTAG

>U1-1pA: probe used for removal of U1 snRNA

AAAAAAAAAAAAAAAAAAAACACCTGCCTGATCATGGT

>U1-2pA: probe used for removal of U1 snRNA

AAAAAAAAAAAAAAAAAAAACTACCACAAATTATGCAGTCG

>U2-1pA: probe used for removal of U2 snRNA

AAAAAAAAAAAAAAAAAAAACAGATACTACACTTGATCT

>U2-2pA: probe used for removal of U2 snRNA

AAAAAAAAAAAAAAAAAAAAGGAGCGGAGGGAGCAAGCT

>U4-1pA: probe used for removal of U4 snRNA

AAAAAAAAAAAAAAAAAAAAGGGGTATTGGGAAAAGTTTTC

>U4-2pA: probe used for removal of U4 snRNA

AAAAAAAAAAAAAAAAAAAAGCCGACTATATTGCAAGTCG

>U5-1pA: probe used for removal of U5 snRNA
AAAAAAAAAAAAAAAAAAAAGCCTCAAAAAATTCCACGAC

>U5-2pA: probe used for removal of U5 snRNA

AAAAAAAAAAAAAAAAAAAAGGCGAAAGATTTATACGATC
